# Efficient DNA sequence compression with neural networks

**DOI:** 10.1093/gigascience/giaa119

**Published:** 2020-11-11

**Authors:** Milton Silva, Diogo Pratas, Armando J Pinho

**Affiliations:** Institute of Electronics and Informatics Engineering of Aveiro, University of Aveiro, Campus Universitário de Santiago, 3810-193 Aveiro, Portugal; Department of Electronics Telecommunications and Informatics, University of Aveiro, Campus Universitário de Santiago, 3810-193 Aveiro, Portugal; Institute of Electronics and Informatics Engineering of Aveiro, University of Aveiro, Campus Universitário de Santiago, 3810-193 Aveiro, Portugal; Department of Electronics Telecommunications and Informatics, University of Aveiro, Campus Universitário de Santiago, 3810-193 Aveiro, Portugal; Department of Virology, University of Helsinki, Haartmaninkatu 3, 00014 Helsinki, Finland; Institute of Electronics and Informatics Engineering of Aveiro, University of Aveiro, Campus Universitário de Santiago, 3810-193 Aveiro, Portugal; Department of Electronics Telecommunications and Informatics, University of Aveiro, Campus Universitário de Santiago, 3810-193 Aveiro, Portugal

**Keywords:** lossless data compression, DNA sequence compression, context mixing, neural networks, mixture of experts

## Abstract

**Background:**

The increasing production of genomic data has led to an intensified need for models that can cope efficiently with the lossless compression of DNA sequences. Important applications include long-term storage and compression-based data analysis. In the literature, only a few recent articles propose the use of neural networks for DNA sequence compression. However, they fall short when compared with specific DNA compression tools, such as GeCo2. This limitation is due to the absence of models specifically designed for DNA sequences. In this work, we combine the power of neural networks with specific DNA models. For this purpose, we created GeCo3, a new genomic sequence compressor that uses neural networks for mixing multiple context and substitution-tolerant context models.

**Findings:**

We benchmark GeCo3 as a reference-free DNA compressor in 5 datasets, including a balanced and comprehensive dataset of DNA sequences, the Y-chromosome and human mitogenome, 2 compilations of archaeal and virus genomes, 4 whole genomes, and 2 collections of FASTQ data of a human virome and ancient DNA. GeCo3 achieves a solid improvement in compression over the previous version (GeCo2) of $2.4\%$, $7.1\%$, $6.1\%$, $5.8\%$, and $6.0\%$, respectively. To test its performance as a reference-based DNA compressor, we benchmark GeCo3 in 4 datasets constituted by the pairwise compression of the chromosomes of the genomes of several primates. GeCo3 improves the compression in $12.4\%$, $11.7\%$, $10.8\%$, and $10.1\%$ over the state of the art. The cost of this compression improvement is some additional computational time (1.7–3 times slower than GeCo2). The RAM use is constant, and the tool scales efficiently, independently of the sequence size. Overall, these values outperform the state of the art.

**Conclusions:**

GeCo3 is a genomic sequence compressor with a neural network mixing approach that provides additional gains over top specific genomic compressors. The proposed mixing method is portable, requiring only the probabilities of the models as inputs, providing easy adaptation to other data compressors or compression-based data analysis tools. GeCo3 is released under GPLv3 and is available for free download at https://github.com/cobilab/geco3.

## Introduction

The DNA sequencing rate is increasing exponentially, stretching genomics storage requirements to unprecedented dimensions. Several projections show that by the year 2025, between 2 and 40 EB of additional storage will be needed per year [1]. Discarding a substantial fraction of the data is not a feasible alternative, given its high importance in many contexts, e.g., in biomedical (e.g., personalized medicine) and anthropological fields.

The representation of genomic data usually consists of DNA sequences accompanied by additional channels, such as headers, quality scores, and variant positions, among others, that vary from type and purpose. Different file formats store the sequence with subsets of these metadata, but the core remains the DNA sequences. The compression of these sequences has been widely approached with general- and specific-purpose compressors; the latter are now coming into frequent use given their substantial compression gains.

Specialized DNA compressors achieve substantially higher compression than general-purpose because most of these compressors use various models that take into account specific properties of DNA, such as inverted repeats and high level of substitutions [2, 3]. However, the efficient combination of multiple models for DNA sequence compression is not a trivial problem. The complexity associated with the development of improved algorithms to combine those predictions [4] and the specificities of the genomic data, namely, heterogeneity and non-stationarity, delivers a highly demanding task.

In this article, we address the problem of combining the predictions of different models to produce an improved predictive model and, by consequence, improve the compression of DNA sequences. Accordingly, we take the specific DNA models from GeCo2 [3], namely, the context and substitution-tolerant context models [5], and implement a mixture of these models with a neural network.

Therefore, instead of combining only the models’ predictions with the algebraic combiner of GeCo2, where weights are attributed to each model and updated on the basis of the model performance with a particular forgetting factor, we improve the mixture of experts using ensemble methods [6].

Specifically, we use a stacked generalization approach [7], namely, applying a neural network metamodel that takes as inputs the outputs of other models and is trained to learn the mapping between the models’ outputs and the actual correct outputs. To implement the stack generalization, we use a multilayer perceptron. This network takes as inputs the probabilities of each model, as well as derived features [8] that represent past model performance, while it outputs the probabilities for each symbol, which are redirected to an arithmetic encoder.

For evaluation, we created a new DNA compression tool (GeCo3) and benchmark it for both reference-free and referential compression. Nine datasets are used for reference-free and reference-based compression benchmarks, containing different sequence nature, lengths, and redundancy levels.

The results show a consistent improvement in the compression ratio of GeCo3 over state-of-the-art DNA compressors, in both reference-free and reference-based approaches, enabling the use of GeCo3 as a long-term storage tool.

Although data compression is the natural approach for decreasing the storage of DNA sequences losslessly [9], it can also be efficiently applied to sequence analysis and prediction using special-purpose compressors [10–12]. Therefore, this improvement also enables increasing the precision of DNA sequence compression–based analysis tools. To facilitate the export of the mixing method to other data compression or data analysis tools, we provide the reusable and modular mixer code and instructions on how to integrate it easily.

In the following subsection, we provide background on reference-free and reference-based DNA sequence compression. Then, we describe GeCo3 in detail and, finally, we provide the benchmark results and some discussion.

### DNA sequence compression

Genomes are found in the most diverse places, e.g., in extreme environments such as uranium mines [13], in soft and hard tissues [14, 15], ancient cadavers [16], marine environments [17], or deep subterranean habitats [18]. The environment and species interactions are a key for genome adaptation, providing a wide diversity in characteristics, namely, high copy number, high heterogeneity, high level of substitution mutations, or multiple rearrangements, such as fissions, fusions, translocations, or inverted repeats [19, 20]. Additionally, because genomic (DNA) sequences are an output of biochemical and computational methods, these sequences may have other alteration sources, e.g., contamination [21], environmental factors [22, 23], pathogenic species included in the samples [24, 25], and unknown sources [26]. Therefore, representing genomic sequences requires the ability to model heterogeneous, dynamic, incomplete, and imperfect information [27].

The above specific characteristics led to the development of the field of the study and construction of specific genomic data compressors [28, 29]. This field, now 27 years old, started with Biocompress [30]. Subsequently, several algorithms emerged, mostly modeling the existence of exact or approximate repeated and inverted repeated regions, through the use of simple bit encoding, context modeling, or dictionary approaches [2, 3, 31–52, 53–64].

The development of the FASTA format permitted the standardization of the co-existence of DNA sequences (in a visible horizontal range) along with annotations (headers). Usually, the DNA sequence is substantially the most abundant part of these data, and, hence, multiple tools use specialized DNA compression algorithms combined with simple header coding, namely, Deliminate [65], MFCompress [66], and NAF [67]. Notwithstanding, for purposes of comparison with DNA sequence compressors, setting a minimal header, asymptotically, increases its irrelevance relative to the DNA sequence according to its size.

From all the previous algorithms, the most efficient according to compression ratio in the wide diversity of DNA sequences are XM [43], GeCo2 [3], and Jarvis [64]. These compressors apply statistical and algorithmic model mixtures combined with arithmetic encoding. Specifically, the XM algorithm [43] combines 3 types of experts, namely, repeat models, a low-order context model, and a short memory context model of 512 B. The GeCo2 algorithm [3] uses soft-blending cooperation between context models and substitution-tolerant context models [5] with a specific forgetting factor for each model. The Jarvis compressor [64] uses a competitive prediction model to estimate, for each symbol, the best class of models to be used; there are 2 classes of models: weighted context models and weighted stochastic repeat models, where both classes of models use specific sub-programs to handle inverted repeats efficiently.

Some compressors use a reference genome as an additional input. This approach is called referential compression, and it started to gain momentum in 2009 [68, 69]. Referential compressors attained substantially higher compression ratios compared with reference-free compressors. The resulting compressed lengths can be hundreds or thousands of times smaller than the original file [70, 71]. As an example, an entire human genome of ∼3 GB can be compressed to 4 MB by referential compression; on the other hand, a reference-free compressor minimizes the data to ∼580 MB. The majority of reference-based compression algorithms use dictionaries, repeat models, or context models [3, 54, 68–80]. From the previous compressors, the most productive, according to compression ratio, are HiRGC [78], GeCo2 [3], iDoComp [70], GDC2 [71], and HRCM [80]. HiRGC [78] is based on a 2-bit encoding scheme and an advanced greedy-matching search on a hash table. GeCo2 [3] is described above. iDoComp [70] uses a suffix array for loading the reference and later applies a greedy parsing of the target that benefits the substitutional single-nucleotide polymorphisms that occur in higher number. GDC2 [71] performs a Ziv-Lempel factoring, combined with a second-level factoring and followed by arithmetic coding. HRCM [80] explores sequence information extraction, followed by sequence information matching and further encoding.

The use of neural networks to compress DNA sequences is seen in DeepDNA [62]. DeepDNA is a special-purpose DNA compressor without specialized models. It uses a hybrid approach with a convolutional layer to capture the genome’s local features and a recurrent layer to model long-term dependencies.

In general-purpose sequence compressors, the idea of using neural networks to mix probabilities is seen in Mahoney [4]. In this case, it is called logistic mixing. Logistic mixing can be viewed as using a neural network without hidden layers and a simpler update rule than backpropagation. Other general-purpose compressors followed the same line, namely, Cmix [81] and DeepZip [82]. Cmix [81] uses recurrent neural networks trained with stochastic gradient descent for context mixing. DeepZip [82] also uses recurrent neural networks, both as predictors (models) and as mixers.

Although the best general-purpose compressors use complex computational models, namely, based on neural networks, it has been shown that they still have lower compression capabilities (5–10%) using substantially higher computational time according to the most efficient specific compressors [82]. The discrepancy in precision is higher when the method is designed for fast computations [83]. The main reason that the best general-purpose algorithms (using neural networks) are not so efficient is that they do not use specific DNA models that take into account the algorithmic nature of genomic sequences, which harms the model sensitivity.

In this article, we combine the sensitivity of specific DNA models, namely, the use of multiple context models combined with DNA-specific algorithmic models, with the power of neural networks for context mixing.

## Methods

In this section, we present the methods that describe the proposed compressor (GeCo3). GeCo3 uses a combination of multiple context models and substitution-tolerant context models of several order-depths. The neural network provides an efficient combination of these models. Therefore, we describe the new method with the main focus on the neural network, including the inputs, updates, outputs, and training process.

### Neural network structure

**Figure 1:**
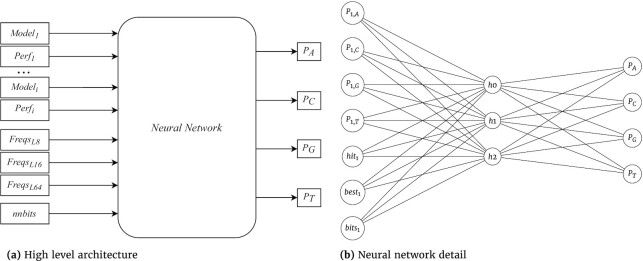
Mixer architecture: (a) High-level overview of inputs to the neural network (mixer) used in GeCo3. Model_1_ through Model_*i*_ represent the GeCo2 model outputs (probabilities for A, C, T, G). Perf represents the performance metrics (hit, best, bits) for each model. Freqs are the frequencies for the last 8, 16, and 64 symbols. nnbits is a moving average of the approximate number of bits that the neural network is producing. The network outputs represent the non-normalized probabilities for each DNA symbol. (b) A fully connected neural network with 1 hidden layer. For illustration purposes, this neural network only has the inputs corresponding to 1 model and the 3 features that evaluate the model performance. The frequencies of the last 8, 16, and 64 symbols, as well as the nnbits and the bias neurons, are omitted.

The model mixing is constructed using a feed-forward artificial neural network trained with stochastic gradient descent [84]. This choice is motivated by implementation simplicity and competitive performance compared with more complex neural networks [85]. The activation function for this network is the sigmoid, and the loss function is the mean squared error. The network structure is fully connected with 1 hidden layer, as seen in Fig. 1b. One bias neuron is used for the input and hidden layer, while the weights respect the Xavier initialization according to Glorot and Bengio [86]. Although we empirically tested different activation functions (ReLu, TanH) and a higher number of hidden layers, the most efficient structure was obtained with the previous description.

We introduced 2 parameters for the GeCo3 compression tool in order to control the number of nodes of the hidden layer and the learning rate. These parameters are written in the compressed file header to ensure a lossless decompression.

### Neural network inputs

The stretched probabilities of each symbol are used as inputs to the network. These are given by

(1) \begin{equation*} p_{i, j} = \mathrm{stretch}\left(\frac{1 + f_{i, j}}{\sum \nolimits _{m \in \Theta } 1 + f_{i, m}}\right) - \mathrm{stretch}\left(\mathrm{mean}_p\right), \end{equation*}

where *f_i, j_* is the frequency of symbol *j* for model *i* with Θ as the set of all symbol and mean_*p*_ is the mean probability of each symbol.

We stretch the probabilities according to the work of Mahoney [4]. The effects of stretching can be seen in Supplementary Section 1 (Stretching function plot). The inputs are normalized for forcing the average to be close to zero by subtracting the stretched mean probability, which, for the case of DNA, we assume to be 0.25. The normalization and its motivation are explained in LeCun et al. [87]. Stretching the probabilities has the effect of scaling them in a non-linear way, which increases the weights of probabilities near 0 and 1.

The context models, substitution-tolerant context models, and the mixed probabilities of GeCo2 are used as input models. This inclusion means that the mixing done in GeCo2 is not discarded but is used as an additional input to the neural network.

We extract features from the context (the last *n* symbols) and also calculate model and network performance indicators to improve the network predictions. These are used as inputs to the neural network. Three performance indicators are derived for each mode according to the names “hit," “best," and “bits." These features correspond to 3 input nodes per model, as seen in Fig. 1b.

To measure how precise model *i* is voting, we use

(2) \begin{equation*} \mathrm{hit}_{i, n}= \left\lbrace \begin{array}{@{}l@{\quad }l@{}}\mathrm{hit}_{i, n-1}, & \text{if}\ \forall x, y \in \Theta : p_{i, x} = p_{i, y} \\ \mathrm{hit}_{i, n-1} + 0.1, & \text{if}\ \forall x \in \Theta : p_{i, \mathrm{sym}} > p_{i, x} \\ \mathrm{hit}_{i, n-1} - 0.1, & \text{otherwise}. \end{array}\right. \end{equation*}

The symbol with the highest probability is considered the vote of the model. Each time the model votes correctly, hit is increased. If the model abstains (probabilities of each symbol are equal), then hit remains the same; otherwise, it decreases.

For each model, we also measure whether it has assigned the highest probability to the correct symbol, compared to all other models. This is given by

(3) \begin{equation*} \mathrm{best}_{i, n}= \left\lbrace \begin{array}{@{}l@{\quad }l@{}}\mathrm{best}_{i, n-1}, & \text{if}\ \forall x, y \in \Theta : p_{i, x} = p_{i, y} \\ \mathrm{best}_{i, n-1} + 0.1, & \text{if}\ p_{i, \mathrm{sym}} \ge p_{k, \mathrm{sym}} \\ \mathrm{best}_{i, n-1} - 0.1, & \text{otherwise}. \end{array}\right. \end{equation*}

The update rules for best are similar to those for hit, and both have a domain of [−1, 1].

As an approximation to the average number of bits the model would output, we use an exponential moving average

(4) \begin{equation*} \mathrm{bits}_{i, n} = \alpha _1 \cdot \left[-\log _2(p_{i, \mathrm{sym}}) + \log _2(\mathrm{mean}_p)\right] + (1 - \alpha _1) \cdot \mathrm{bits}_{i, n-1}, \end{equation*}

with α_1_ = 0.15. This input is also normalized such that the average value is close to zero.

In equations (2), (3), and (4), *p*_*i*, sym_ is the probability assigned by model *i* to the actual symbol in the sequence. To reach these features and their constants, we tested each with a couple of files from 1 dataset and adjusted until we found a value that produced satisfactory results.

The features extracted from the context are the probabilities of each symbol for the last 8, 16, and 64 symbols. These represent a total of 12 input nodes. In Fig. 1a, these nodes are represented by Freqs_L8_, Freqs_L16_, and Freqs_L64_. For example, to obtain the probabilities for the last 8 symbols with the sequence ACAGTAAA, the number of A’s is divided by the number of total symbols, so the frequency of symbol A is 5/8 and for the other symbols is 1/8. These probabilities are then scaled to fit between −1 and 1.

In Fig. 1a, nnbits represents the exponential moving average of the approximate number of bits and is given by

(5) \begin{eqnarray*}
\mathrm{nnbits}_{n} = \alpha _2 \cdot \left[-\log _2(p_{\mathrm{sym}}) + \log _2(\mathrm{mean}_p)\right] + (1 - \alpha _2) \cdot \mathrm{nnbits}_{n-1}, \nonumber \\ \end{eqnarray*}

with *p*_sym_ as the probability the network assigned to the correct symbol and α_2_ = 0.5.

### Updating model performance features

As an example of how to update the features, consider 2 symbols and 3 models, and assume that all features start equal to zero. Model 1 assigns the probabilities [0.5, 0.5], meaning that the model abstains and, as such, no change is made to hit or best. Also, bits_1_ would be equal to zero. The probabilities for model 2 and 3 are [0.7, 0.3] and [0.8, 0.2], respectively. Assuming that the models voted correctly, then hit is now 0 + 0.1 = 0.1 for both. Because model 3 assigned the highest probability to the correct symbol, then best_3_ is now 0 + 0.1 = 0.1 and best_2_ becomes −0.1. Moreover, bits_2_ would become bits_2_ = 0.15 · [−log_2_(0.7) + log_2_(0.5)] and bits_3_ = 0.15 · [− log_2_(0.8) + log_2_(0.5)].

### Neural network outputs and training

One node per symbol is used as output from the network. After the result is transferred to the encoder, the network is trained with the current symbol using the learning rate specified within the program input.

When compared to GeCo2, the results of the new mixing contain 2 main differences. First, the sum of output nodes is different from 1. This outcome is corrected by dividing the node’s output by the sum of all nodes. The second difference is that the new approach outputs probabilities in the range [0, 1], while in GeCo2, the mixing always yielded probabilities inside the range of the models.

## Results

In this section, we benchmark GeCo3 against state-of-the-art tools in both reference-free and referential compression approaches. In the following subsection, we describe the datasets and materials used for the benchmark, followed by the comparison with GeCo2 using different characteristics, number of models, and data redundancy. Finally, we provide the full benchmark for the 9 datasets.

### Datasets and materials

The benchmark includes 9 datasets. Five datasets are selected for reference-free compression, including

1. **DS1**: 2 compilations of FASTQ data, namely, a human virome (Virome) [88] and ancient DNA from a Denisova individual (Denisova) [89];
2. **DS2**: 4 whole genomes: human (HoSaC), chimpanzee (PaTrC), gorilla (GoGoC), and the Norway spruce (PiAbC);
3. **DS3**: 2 compilations of archaeal (Archaea) and viral genomes (Virus);
4. **DS4**: highly repetitive DNA with the human Y-chromosome (HoSaY) and a human mitogenome collection (Mito) (proposed in [90]);
5. **DS5**: a comprehensive-balanced dataset (proposed in [91]), containing the following sequences:
  1. HoSa: chromosome 4 of the reference human genome
  2. GaGa: chromosome 2 of *Gallus gallus*;
  3. DaRe: chromosome 3 of *Danio rerio*;
  4. OrSa: chromosome 1 of *Oryza sativa Japonica*;
  5. DrMe: chromosome 2 of *Drosophila miranda*;
  6. EnIn: genome of *Entamoeba invadens*;
  7. ScPo: genome of *Schizosaccharomyces pomb*;
  8. PlFa: genome of *Plasmodium falciparum*;
  9. EsCo: genome of *Escherichia coli*;
  10. HaHi: genome of *Haloarcula hispanica*;
  11. AeCa: genome of *Aeropyrum camini*;
  12. HePy: genome of *Helicobacter pylori*;
  13. YeMi: genome of Yellowstone Lake mimivirus;
  14. AgPh: genome of *Aggregatibacter* phage S1249;
  15. BuEb: genome of *Bundibugyo ebolavirus*.

On the other hand, to benchmark the reference-based approach, we use the complete genomes of 4 primates (human, gorilla, chimpanzee, and orangutan) with a pairwise chromosomal compression. Non-human chromosomes are concatenated to match the human chromosomal fusion [92]. For each chromosomal pair, the following compression was performed:

1. **DSR1**: chimpanzee (PT) using human (HS) as a reference;
2. **DSR2**: orangutan (PA) using human (HS) as a reference;
3. **DSR3**: gorilla (GG) using human (HS) as a reference;
4. **DSR4**: human (HS) using gorilla (GG) as a reference.

All the materials to replicate the results, including the sequence identifiers, URL, filtering applications, and associated commands, can be found in Supplementary Section 8 (Reproducibility).

### Neural network mixing compression

To assess the performance of the neural network mixing, we compare GeCo2 with GeCo3. To ensure a fair comparison, the compression modes, including the models and parameters, are kept identical for both programs.

In Table 1, GeCo2 and GeCo3 are compared using the compression modes published by Pratas et al. [3]. The overall compression improves by $1.93\%$, and the mean improvement is $1.06\%$. The larger sequences (larger than ScPo) have mean improvements of $2.04\%$, while the remaining have modest improvements of $0.4\%$. Only the 2 smallest sequences show negative improvement, given the absence of enough time to train the network. Additionally, the 8 B that are used to transmit the 2 network parameters to the decompressor are a significant percentage of the total size, unlike in larger sequences. Overall, GeCo3 improves the compression of the whole dataset by >$1.9\%$.

**Table 1:** Number of bytes needed to represent each DNA sequence for GeCo2 and GeCo3 compressors

ID	GeCo2 bytes	GeCo3 bytes	GeCo2 secs	GeCo3 secs	Mode	Learning rate	Hidden nodes
HoSa	38,845,642	**37,891,143**	223	598	12	0.03	64
GaGa	33,877,671	**33,411,628**	160	424	11	0.03	64
DaRe	11,488,819	**11,189,716**	64	189	10	0.03	64
OrSa	8,646,543	**8,434,878**	44	133	10	0.03	64
DrMe	7,481,093	**7,379,992**	33	99	10	0.03	64
EnIn	5,170,889	**5,066,670**	26	75	9	0.05	64
ScPo	2,518,963	**2,511,054**	11	24	8	0.03	40
PlFa	1,925,726	**1,906,919**	10	22	7	0.03	40
EsCo	1,098,552	**1,094,298**	2	8	6	0.03	40
HaHi	902,831	**896,037**	2	6	5	0.04	40
AeCa	380,115	**377,343**	1	2	5	0.04	16
HePy	375,481	**373,583**	1	3	4	0.04	40
YeMi	16,798	**16,793**	0	0	3	0.09	24
AgPh	**10,708**	10,715	0	0	2	0.06	16
BuEb	**4,686**	**4,686**	0	0	1	0.06	8
Total	112,744,517	**110,565,455**	577	1,583			

The column mode applies to both compression methods, while the learning rate and the number of hidden nodes only apply to the latter. Bold indicates the best compression.

### Neural network mixing computational resources

Regarding computational resources, the mixing modification is 2.7 times slower, as reported in Table 1. The computation was performed on an Intel^(R)^ Core^TM^ i7-6700 CPU at 3.40 GHz running Linux 5.4.0 with the scaling governor set to performance and 32 GB of RAM. The new mixing approach is always slower because GeCo2’s mixing is still used, not as a result of the encoder, but rather as an input to the network. The difference in RAM use of both approaches is <1 MB, which corresponds to the size of the neural network and the derived features for each model.

The number of hidden nodes is chosen to fit in the vector registers in order to take full advantage of the vectorized instructions. Accordingly, we set the number of hidden nodes as a multiple of 8, where floating points of 4 B represent the nodes and 32 B represent the vector registers.

### Effects of the hidden layer size on mixing

**Figure 2:**
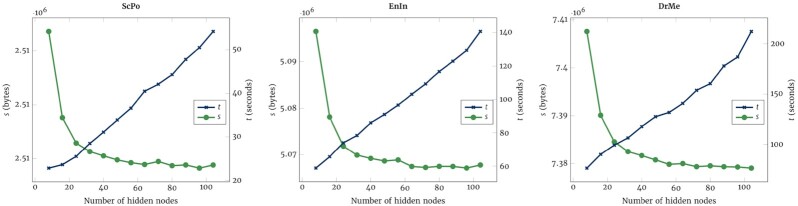
Number of bytes (*s*) and time (*t*) according to the number of hidden nodes for the reference-free compression of ScPo, EnIn, and DrMe sequence genomes.

Increasing or decreasing the number of hidden nodes affects the number of weights, and it also affects compression, as can be seen in Fig. 2. Increasing the number of nodes increases the compression up to a point. This point varies from sequence to sequence; however, the most abrupt gains in compression generally occur until 24 hidden nodes. As expected, increasing the number of hidden nodes leads to an increase in execution time and a progressive decline of compression gain. These results are also consistent in referential compression as seen in Supplementary Section 6 (Referential hidden nodes effect).

### The importance of derived features on mixing

We removed the derived features from the inputs to the network to assess its effect on the mixing performance. The results are presented in Table 2.

**Table 2:** Number of bytes needed to represent each DNA sequence using the GeCo3 compressor with specific conditions.

ID	Models	Models + GeCo2	Models + derived
HoSa	38,556,039	38,153,358	**37,943,933**
GaGa	33,758,606	33,548,929	**33,444,816**
DaRe	11,615,937	11,280,688	**11,251,390**
OrSa	8,694,790	8,517,947	**8,471,715**
DrMe	7,475,341	7,414,919	**7,392,290**
EnIn	5,183,237	5,095,391	**5,087,359**
ScPo	2,524,818	2,514,188	**2,513,085**
PlFa	1,928,282	1,912,745	**1,912,176**
EsCo	1,104,646	1,095,589	**1,096,255**
HaHi	903,019	898,280	**898,145**
AeCa	378,226	377,857	**377,696**
HePy	379,285	**374,364**	374,975
YeMi	16,901	**16,827**	16,882
AgPh	10,744	**10,727**	10,731
BuEb	**4,694**	4,696	4,698
Total	112,534,565	111,216,505	110,796,146

For the column named “Models," only the context models and tolerant context models of GeCo2 were used as network inputs. For “Models + GeCo2,” the result of GeCo2 mixing was also used as input. With “Models + Derived” the inputs for the network were the same as “Models” with the derived features added. The compression modes are the same as in Table 1. Bold indicates the best compression.

When using just the models’ probabilities as inputs, the compression is more efficient than GeCo2 by a small margin ($0.18\%$), while, in the majority of the sequences, there is no improvement. By adding the result of the GeCo2 mixing as an input, the improvement increases to $1.36\%$. The gain escalates, having an improvement of $1.73\%$, when using the context models and tolerant context models as inputs and the derived features.

### Scaling the number of models

GeCo2 and GeCo3 contain several modes (compression levels), which are parameterized combinations of models with diverse neural network characteristics. To see how the compression of the new approach scales with more models, we introduced mode 16 with a total of 21 models. This new mode was used to compress the sequences of HoSa to HePy (by size order). For the remaining sequences, the same models were used as in Table 1. We used this approach because increasing the number of models was incapable of improving the compression of GeCo3 and GeCo2, given the smaller dimensions of these sequences. The number of hidden nodes was also adjusted until no tangible improvements in compression were observed.

The results in Table 3 show that the distance between the approaches increases from $1.93\%$ to $2.43\%$. The time difference reduces from 2.7 to 2.0 times. This reduction is due to the increased percentage of time spent by the higher-order context models. These results show that neural network mixing can scale with the number of models. The forgetting factors for this new mode were not tuned, due to the use of a large number of models. Therefore, with this tuning, additional gains can be observed. Nevertheless, this shows another advantage of this new mixing, which is that there are only 2 parameters that need tuning regardless of the number of models. As the sequence size and the number of models increase, there is almost no tuning required, with the optimal values being ∼0.03 for the learning rate and 64 hidden nodes.

**Table 3:** Size and time needed to represent a DNA sequence for NAF, XM, Jarvis, GeCo2, and GeCo3

DS	ID	NAF		XM		Jarvis		GeCo2		GeCo3	
		Size	Time	Size	Time	Size	Time	Size	Time	Size	Time
1	Denisova	25.36 GB	25h22m	*	*	/	/	20.61 GB	23h18m	**19.55 GB**	71h19m
	Virome	4.72 GB	6h01m	*	*	/	/	3.17 GB	8h45m	**2.79 GB**	24h32m
	Total	30.08 GB	31h23m	*	*	/	/	23.78 GB	32h04m	**22.34 GB**	95h51m
2	PiAbC	2.29 GB	2h45m	*	*	/	/	1.86 GB	4h02m	**1.71 GB**	9h21m
	HoSaC	634.07 MB	38m	*	*	/	/	579.66 MB	53m12s	**560.88 MB**	2h14m
	PaTrC	619.48 MB	37m	*	*	/	/	569.40 MB	51m40s	**551.54 MB**	2h08m
	GoGoC	603.39 MB	36m	*	*	/	/	556.54 MB	49m57s	**539.30 MB**	2h04m
	Total	4.15 GB	4h36m	*	*	/	/	3.57 GB	6h37m	**3.36 GB**	15h49m
3	Archaea	128.09 MB	7m	103.01 MB?	1h41m	**96.66 MB**	57m	103.70 MB	30m	97.87 MB	55m
	Virus	85.51 MB	6m	63.93 MB?	1h35m	**61.19 MB**	1h35m	65.63 MB	29m	61.19 MB	55m
	Total	213.60 MB	14m	166.93 MB?	3h16m	**157.84 MB**	2h32m	169.34 MB	1h00m	159.07 MB	1h51m
4	Mito	35.93 MB	2m32s	28.12 MB?	47m11s	**27.11 MB**	16m1s	30.40 MB	11m26s	28.17 MB	21m31s
	HoSaY	5.17 MB	11s	3.88 MB?	3m25s	3.93 MB	1m45s	4.08 MB	1m15s	**3.85 MB**	2m21s
	Total	41.10 MB	2m43s	32.01 MB?	50m36s	**31.04 MB**	17m46s	34.48 MB	12m41s	32.03 MB	23m52s
5	HoSa	41.73 MB	2m06s	38.66 MB?	29m26s	38.66 MB	4m33s	38.79 MB	11m17s	**37.56 MB**	22m39s
	GaGa	35.57 MB	1m38s	33.83 MB?	22m20s	33.70 MB	2m38s	33.75 MB	8m43s	**33.26 MB**	17m38s
	DaRe	12.83 MB	32s	11.17 MB?	8m59s	11.17 MB	1m32s	11.44 MB	3m40s	**10.97 MB**	7m32s
	OrSa	9.53 MB	21s	8.48 MB?	6m39s	8.45 MB	1m14s	8.60 MB	2m37s	**8.34 MB**	5m17s
	DrMe	7.85 MB	15s	7.53 MB?	5m01s	7.49 MB	22s	7.47 MB	1m57s	**7.36 MB**	3m50s
	EnIn	5.87 MB	12s	5.12 MB?	3m19s	5.09 MB	36s	5.14 MB	1m37s	**5.02 MB**	3m12s
	ScPo	2.59 MB	4s	2.53 MB	55s	2.52 MB	11s	2.52 MB	44s	**2.51 MB**	1m21s
	PlFa	2.02 MB	4s	1.92 MB	59s	1.92 MB	10s	1.93 MB	37s	**1.90 MB**	1m09s
	EsCo	1.15 MB	2s	1.11 MB	13s	1.10 MB	4s	1.10 MB	24s	**1.09 MB**	39s
	HaHi	948.69 kB	2s	914.87 kB	16s	899.47 kB	2s	899.17 kB	21s	**889.51 kB**	34s
	AeCa	396.82 kB	1s	387.00 kB	3s	380.51 kB	1s	381.29 kB	13s	**376.97 kB**	18s
	HePy	404.55 kB	1s	384.30 kB	4s	374.37 kB	1s	375.66 kB	13s	**371.62 kB**	19s
	YeMi	17.35 kB	1s	16.84 kB	0s	16.87 kB	0s	16.80 kB	0s	**16.79 kB**	0s
	AgPh	11.02 kB	1s	10.71 kB	0s	10.75 kB	0s	**10.71 kB**	0s	10.72 kB	0s
	BuEb	4.81 kB	1s	**4.64 kB**	0s	4.70 kB	0s	4.69 kB	0s	4.69 KB	0s
	Total	120.94 MB	5m22s	112.07 MB	1h18m14s	111.79 MB	11m24s	112.42 MB	32m23s	**109.68 MB**	1h04m28s

For DNA Sequence 5 (DS5), Jarvis uses the same configuration as in [64]; for DS4 and DS3 it uses Level 7. XM uses the default configuration. NAF uses the highest compression level (22). GeCo2 and GeCo3 use Mode 16 for DS5, except for BuEb, AgPh, and YeMi, which use the configurations of Table 1. For DS4 and DS3 the models are “-tm 3:1:1:1:0.8/0:0:0 -tm 6:1:1:1:0.85/0:0:0 -tm 9:1:1:1:0.85/0:0:0 -tm 12:10:0:1:0.85/0:0:0 -tm 15:200:1:10:0.85/2:1:0.85 -tm 17:200:1:10:0.85/2:1:0.85 -tm 20:500:1:40:0.85/5:20:0.85”, DS2 uses “-tm 3:1:1:1:0.70/0:0:0 -tm 8:1:1:1:0.85/0:0:0 -tm 13:10:0:1:0.85/0:0:0 -tm 19:500:1:40:0.85/5:20:0.85”, and Virome uses “-tm 7:1:1:1:0.8/0:0:0 -tm 13:10:0:1:0.95/0:0:0 -tm 19:500:1:40:0.95/5:20:0.95”. Denisova uses the same models as Virome but with inversions turned off. GeCo3 uses a learning rate of 0.03 and 64 hidden nodes for all sequences. *Sequence was not compressed due to an error; /sequence was not compressed due to out of memory; question mark indicate results where the decompression produces different results than the input file. Bold indicates the best compression and underline the fastest.

### Compressing highly repetitive and large sequences

In this subsection, we show how the reference-free compression scales with the new mixing using highly repetitive and extensive sequences, namely, in the gigabyte scale. Four datasets are selected, and the results presented in Table 3.

According to the results from Table 3, GeCo3 compresses the highly repetitive sequences (DS3 and DS4) with an average of $6.6\%$ compared to GeCo2 using 1.9 times more time. For the larger sequences of DS1 and DS2, GeCo3 has a mean compression improvement of $3.2\%$ in the primates, $8.2\%$ in the spruce (PiAbC), $11.8\%$ for the Virome, and $5.2\%$ for Denisova, with a 2.6 times mean slower execution time. These results show that the compression of longer repetitive sequences presents higher compression gains.

### Reference-free sequence compression benchmark

In this subsection, we compare GeCo3 with other specialized reference-free compressors, namely, XM (v3.0) [43], GeCo2 (previously compared), Jarvis [64], and NAF [67]. As presented in Table 3, GeCo3 achieves the best total size in 3 of 5 datasets. In DS3 and DS4, GeCo3 was unable to achieve the best compression, which was delivered by Jarvis. These types of datasets justify this performance. Specifically, DS3 and DS4 contain a high number of identical sequences. These are collections of mitogenomes, archeal and virus, where the variability is very low, which gives an advantage to models of extremely repetitive nature. Such models, also known as weighted stochastic repeat models, are present in Jarvis, unlike in GeCo3. The reason why we excluded these models from GeCo is that they fail in scalability because the RAM increases according to the sequence length. For the larger datasets, DS1 and DS2, Jarvis was unable to compress the sequences even with 32 GB of RAM. On the other hand, GeCo3 has constant RAM, which is not affected by the sequence length but rather only by the mode used.

Comparing GeCo3 against the second-best compressor for each dataset, the compression gain is $6\%$ (vs GeCo2), $5.8\%$ (vs GeCo2), $-0.8\%$ (vs Jarvis), $-3.2\%$ (vs Jarvis), and $1.9\%$ (vs Jarvis) for DS1, DS2, DS3, DS4, and DS5, respectively. For the individual sequences in the datasets, GeCo3 compresses more than the other compressors, except for AgPh, BuEb, Mito, Virus, and Archaea. Tiny sequences compose the AgPh and BuEb dataset, and the neural network does not have enough time to learn, while Mito, Virus, and Archaea have already been mentioned above.

Regarding computational time, GeCo3 is faster than XM per dataset, spending on the average only 0.6 times the time. Against GeCo2, it is slower by 2.1 times on average, and compared to Jarvis, it is 1.1 times slower. NAF is the fastest compressor in the benchmark. Compared to NAF, GeCo3 is between 12 times slower for DS5 and 3 times for DS1.

Regarding computational memory, the maximum amount of RAM used for GeCo2 and GeCo3 was 12.6 GB, Jarvis peaked at 32 GB, XM at 8 GB, and NAF used at most 0.7 GB. Jarvis could not complete the compression for DS1 and DS2 owing to a lack of memory. This issue is a limitation that was mentioned earlier. We also note that XM was unable to decompress some of the sequences. In these cases, the decompressed file has the correct size, but the sequence does not fully match the original file. NAF, GeCo2, and GeCo3 were the only compressors that have been able to compress all the sequences losslessly, independently from the size. The overall results of these compressors show that GeCo3 provides a total compression improvement of $25\%$ and $6\%$ over NAF and GeCo2, respectively.

Compared with general-purpose compressors that achieve the best compression ratios, such as CMIX and DeepZip, GeCo3 is ∼100 times faster. GeCo3 also has better total compression ratio compared to CMIX ($7.7\%$). We could not obtain enough results with DeepZip to make a meaningful comparison. The table with the results can be seen in Supplementary Section 3 (Results for general purpose compressors).

### Reference-based sequence compression benchmark

In this subsection, we benchmark GeCo3 with state-of-the-art referential compressors. The comparison is done between the genomes of different species and not for re-sequenced genomes. Re-sequencing is applied to the same species and, in a general case, limits the domain of applications; e.g., phylogenomic, phylogenetic, or evolutionary analysis.

To run the experiments, we used 4 complete genomes of closely related species: *Homo sapiens* (HS), *Pan troglodytes* (PT), *Gorilla gorilla* (GG) and *Pongo abelii* (PA). The compression for PT, GG, and PA was done using HS as the reference. HS was compressed using GG as a reference. Each chromosome was paired with the corresponding one of the other species. Due to the unavailability of chromosome Y for GG and PA, comparisons that involved these chromosomes were not made. The compressors used in this benchmark are GeCo3, GeCo2, iDoComp [70], GDC2 [71], and HRCM [80]. The FASTA files were filtered such that the resulting file only contained the symbols {*A, C, G, T*}, and a tiny header line. HRCM needs the line size to be limited; therefore, line breaks were added for the files under its compression. However, this approach prevents a direct comparison of total compressed size and time, which we solved using the compression ratio percentage (output_size ÷ input_size × 100) and the speed in kilobytes per second (input_size ÷ 1,000 ÷ seconds_spent). For GeCo2 and GeCo3, 2 approaches of referential compression are considered. One approach is based on conditional compression, where a hybrid of both reference and target models is used. The other approach, called the relative approach, uses exclusively models loaded from the reference sequence. Both types of compression assume causality, which means that with the respective reference sequence, the decompressor is able to decompress without loss. The reason why we benchmark these 2 approaches is that there are many sequence analysis applications for both approaches.

The results are presented in Table 4, showing the total compression ratio and speed for the 4 comparisons. The total compression ratio is the total_output_size ÷ total_input_size × 100 and the total speed is total_input_size ÷ 1,000 ÷ total_seconds_spent. The results show GeCo3 achieving the best compression ratio, in both relative and conditional compression. The latter shows improved compression capabilities, with mean improvements of $11\%$, $35\%$, $38\%$, and $50\%$ over GeCo2, iDoComp, GDC2, and HRCM, respectively. This comes at a cost of being the slowest. The mean increase in time over GeCo2, iDoComp, GDC2, and HRCM is 1.7, 9.8, 2.6, and 7.3 times, respectively. Compared with GeCo2, the total improvement for PT, PA, GG, and HS is $12.4\%$, $11.7\%$, $10.8\%$, and $10.1\%$. The total improvements are similar to the mean improvement per chromosome. The computational RAM of GeCo3 is similar to GeCo2. The complete results per chromosome are shown in Supplementary Section 4 (Complete results for referential compression). These show that in the majority of pairs GeCo3 offers better compression.

**Table 4:** Total referential compression ratio and speed in kB/s

DSR	ID	HRCM		GDC2		iDoComp		GeCo2-r		GeCo3-r		GeCo2-h		GeCo3-h	
		Ratio	Speed	Ratio	Speed	Ratio	Speed	Ratio	Speed	Ratio	Speed	Ratio	Speed	Ratio	Speed
1	HSxPT	6.29	2,006	5.01	841	4.78	2,430	4.16	527	3.65	296	4.02	374	**3.52**	224
2	HSxPA	15.27	1,260	12.24	382	11.31	1,891	7.51	513	6,57	294	7.26	367	**6.41**	222
3	HSxGG	8.80	1,691	7.06	588	6.70	2,201	5.58	516	4.96	293	5.43	369	**4.84**	222
4	GGxHS	9.48	1,773	8.11	712	7.80	2,332	6.43	558	5.81	301	5.77	389	**5.19**	230
	Total	9.96	1,635	8.11	580	7.66	2,195	5.92	529	5.26	296	5.62	375	**4.99**	225

GeCo3 uses 64 hidden nodes and has 0.03 learning rate. The configuration for GeCo2-r and GeCo3-r (relative approach) is “-rm 20:500:1:35:0.95/3:100:0.95 -rm 13:200:1:1:0.95/0:0:0 -rm 10:10:0:0:0.95/0:0:0”. For GeCo2-h and GeCo3-h (conditional approach) the following models were added: “-tm 4:1:0:1:0.9/0:0:0 -tm 17:100:1:10:0.95/2:20:0.95”. iDoComp, GDC2, and HRCM use the default configuration. Bold indicates the best compression and underline the fastest.

In Table S7 of Supplementary Section 4, we show the results for compression of a resequenced genome. In this dataset HRCM achieves the best results, with GeCo3 trailing in both speed (42 times) and ratio ($-363\%$). While these results show that GeCo2 and GeCo3 are not suitable for compressing this type of dataset, the substantial improvement over GeCo2 ($20\%$) hints at the possibility that the new mixer might be useful when integrated into a different type of compressor.

### Estimating the cost for long-term storage

To estimate the cost of long-term storage, we developed a model with the following simplifying assumptions: ≥2 copies are stored; compression is done once and the result is copied to the different backup media; 1 CPU core is at 100% utilization during compression; the cooling and transfer costs are ignored; the computing platform is idle when not compressing;

and no human operator is waiting for the operations to terminate.

Given the assumptions we now show the cost model:

\begin{equation*} \mathrm{Total}_{\mathrm{cost}} = \mathrm{Processing}_{\mathrm{cost}} + \mathrm{Storage}_{\mathrm{cost}} \\ \mathrm{Processing}_{\mathrm{cost}} = \mathrm{Processing}_{\mathrm{time}} \times \mathrm{Power} \times \mathrm{Energy}_{\mathrm{price}} \\ \mathrm{Storage}_{\mathrm{cost}} = N_{\mathrm{copies}} \times \mathrm{Size} \times \mathrm{Size}_{\mathrm{price}}, \end{equation*}

where Processing_time_ is the total time to compress and decompress the sequence.

From [93], we use the single thread load subtracted by the idle value to calculate the power (watts) that a system uses during processing. The mean result for all systems is 34 W. The mean cost of electricity in the world is €0.12 per kWh [94]. The mean storage cost per GB for hard disk drives is €0.04 [95] and for solid-state drives is €0.13 [96].

**Figure 3:**
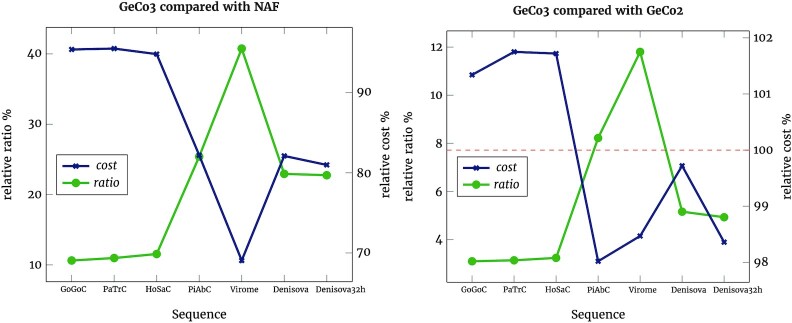
Relative ratio and cost of GeCo3 compared with NAF and GeCo2 for sequences in DS1 and DS2. Higher relative ratios represent greater compression improvements by GeCo3. The cost is calculated assuming €0.13 per GB and the storage of 3 copies. The red dashed line shows the cost threshold. Cost points above the line indicate that GeCo3 is more expensive. Denisova32h represents the results of running the Denisova sequence with 32 instead of 64 hidden nodes.

Assuming €0.13 per GB and 3 copies, the costs for DS1 are €11.86, €9.54, and €9.5 for NAF, GeCo2, and GeCo3, respectively. Using €0.04 per GB and 3 copies, GeCo2 is more cost-effective at €3.12, followed by GeCo3 (€3.46) and NAF (€3.74). In Fig. 3, we show the costs of storing each sequence in DS1 and DS2 with GeCo3 relative to NAF and GeCo2. As hinted by Fig. 2, we also show that the cost of compressing the Denisova sequence is improved when using 32 instead of 64 hidden nodes. The reduction of hidden nodes leads to a negligible decrease in compression ratio ($5.2\%$ to $4.9\%$ vs GeCo2) but a substantial time decrease (3.1 to 2.4 times vs GeCo2).

These results use the average costs, though given the variability of electricity prices, CPU power efficiency and storage costs, the analysis would need to be done for each specific case.

## Discussion

In essence, this article considers the GeCo2 as a base, collecting its specific DNA models, and augments the mixture of models by using a neural network. The primary outcome is a new efficient tool, GeCo3. The results show a compression improvement at the cost of longer execution times and equivalent RAM.

For the evaluated datasets, this approach delivers the best results for the most significant and the highest repetitive sequences. One of the reasons for this is that for small sequences, the network spends a significant percentage of time adjusting. Moreover, we show the importance of selecting and deriving the appropriate network inputs as well as the influence of the number of hidden nodes. These can be used to increase compression at the cost of higher execution times.

Compared to other state-of-the-art compressors, this approach is typically slower but achieves better compression ratios in both reference-free and referential compression. Nevertheless, the compression times can be reduced by decreasing the number of hidden nodes while still improving the ratio.

The GeCo3 reference-free results show an improvement of $25\%$ and $6\%$ over NAF and GeCo2, respectively. In reference-based compression, GeCo3 is able to provide compression gains of $11\%$, $35\%$, $38\%$, and $50\%$ over GeCo2, iDoComp, GDC2, and HRCM, respectively.

The time trade-off and the symmetry of compression-decompression establish GeCo3 as an inappropriate tool for on-the-fly decompression. Tools such as NAF [67] are efficient for this purpose because the computational decompression speed is very high, which for industrial use is mandatory. The purposes of tools such as GeCo3 are in another domain, namely, long-term storage and data analysis.

In particular, the results suggest that long-term storage of extensive databases, e.g., as proposed in [97], would be a good fit for GeCo3.

The steady rise of analysis tools based on DNA sequence compression is showing its potential, with increasing applications and surprising results. Some of the applications are the estimation of the Kolmogorov complexity of genomes [98], rearrangement detection [99], sequence clustering [100], measurement of distances and phylogenetic tree computation [101], and metagenomics [12].

The main advantage of using efficient (lossless) compression-based data analysis is avoidance of overestimation. Many analysis algorithms include multiple thresholds that use a consensus value for what is considered balanced and consistent, leaving space for overestimation. The problem is that using a consensus or average parameter for a specific analysis may overtake the limit of the estimation balance. Because data compression needs the appropriate decompressor to ensure the full recovery of the data, the compressor acts under a boundary that ensures that the limit is never surpassed (Kolmogorov complexity). This property is critical in data analysis because the data in use may be vital and sensitive, mainly when multiple models are used. Without a channel information limit and an efficient mixing model, the information that is embedded in the probability estimation of each model transits to the model choice.

The mixing method used to achieve these results assumes only that probabilities for the symbols are available. Because of this, it can be easily exported to other compressors or compressed-based data analysis tools that use multiple models. GeCo3 shows what compression improvements and execution times can be expected when using neural networks for the mixture of experts in DNA sequence compression.

This article highlights the importance of expert mixing. Mixing has applications in all areas where outcomes have uncertainty and many expert opinions are available. This ranges from compression to climate modeling and, in the future, possibly the creation of legislation. While more traditional methods, such as weighted majority voting, are more efficient and can achieve accurate results, neural networks show promising results. With the development of specialized hardware instructions and data types to be included in general-purpose CPUs [102, 103], neural networks should become an even more attractive option for expert mixing.

**Figure 4:**
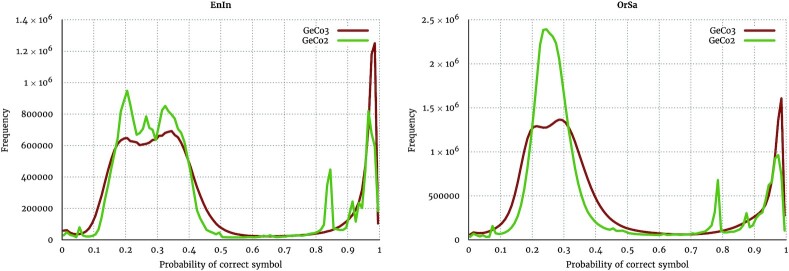
Comparison of histograms using the EnIn (*Entamoeba invadens*) and OrSa (*Oryza sativa*) genome sequences and GeCo2 and GeCo3 as data compressors.

One of the possible reasons this approach has higher compression than GeCo2 is due to the mixing output not being constrained by the inputs. By comparing the histograms in Fig. 4 for the sequences EnIn and OrSa (2 of the sequences with higher gains), we can verify that GeCo3 appears to correct the models’ probabilities >0.8 to probabilities closer to 0.99. Therefore, in some way, it is betting more if ≥4 in 5 chances are accomplished. Referential histograms are presented in Supplementary Section 7 (Referential histograms); these are similar to the ones presented here.

Another improvement is due to the higher percentage of symbols inferred correctly. For dataset 5 (DS5), GeCo3 has a mean improvement of $1.5\%$ in the number of symbols inferred correctly, where only the smallest sequence has a lower hit rate than GeCo2. Supplementary Section 2 (Percentage of symbols guessed correctly) presents the table of hit rate per sequence.

**Figure 5:**
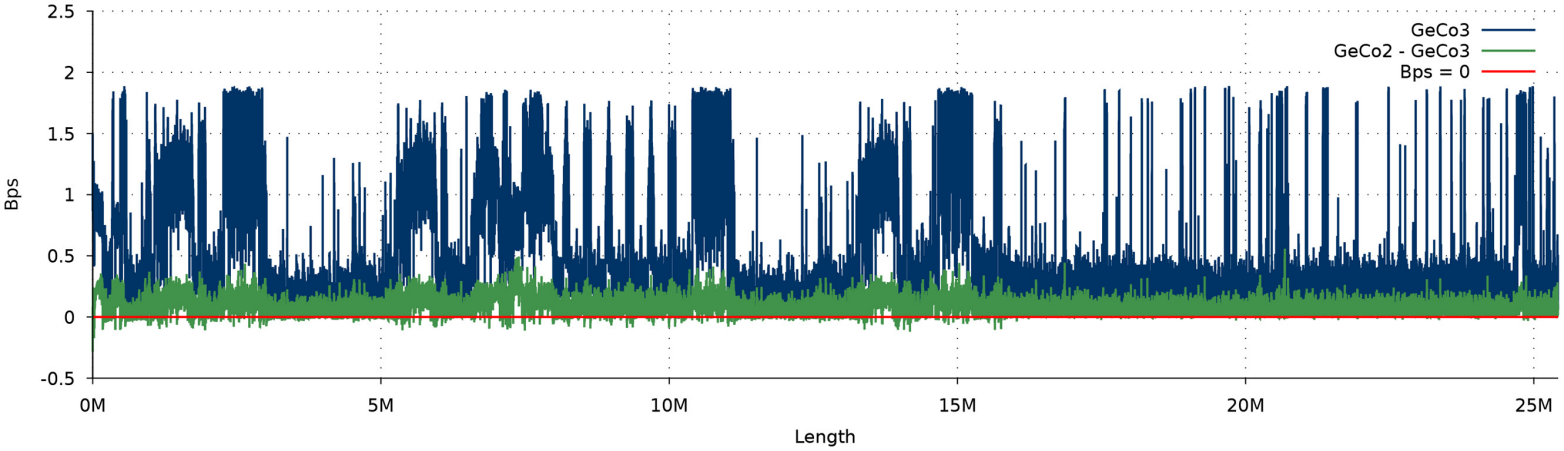
Complexity profile using the smoothed number of bits per symbol (Bps) of GeCo2 subtracted by GeCo3 Bps. The Bps were obtained by referential compression of PT_Y (Chromosome Y from *Pan troglodytes*) with the corresponding *Homo sapiens* chromosome, with the same parameters as in Table 4. Regions where the line rises above zero indicate that GeCo3 compresses more than GeCo2.

For referential compression, we show a complexity profile in Fig. 5. This profile reveals that GeCo3 consistently outputs a lower number of bits per symbol. The gains appear to be larger in places of higher sequence complexity, i.e., in the higher bits per symbol (Bps) regions. These regions are typically where rapid switching between smaller models should occur, suggesting that the neural network mixer can adapt faster than the approach used in GeCo2. Supplementary Section 5 (Referential complexity profiles) presents 2 additional complexity profiles with similar nature.

Finally, the training is maintained during the entire sequence because we found that stopping early leads to worse outcomes. This characteristic might be due to the advantages of over-fitting for non-stationary time series reported by Kim et al. [104].

Additional improvements on the compression of large FASTQ data, e.g., from the Virome and Denisova datasets, can be achieved with complementary techniques based on reordering or metagenomic composition identification. Specifically, the reads of these datasets can be split according to their composition using fast assembly-free and alignment-free methods, namely, extensions of Read-SpaM [105], to take advantage of the similar read proximity to improve the compression substantially.

Whichever the technology and application, the core method that we provide here, namely, for combining specific DNA models with neural networks, enables a substantial improvement in the precision of DNA sequence compression–based data analysis tools and provides a significant reduction of storage requirements associated with DNA sequences.

## Availability of Source Code and Requirements

Project name:  GeCo3

Project home page:  http://github.com/cobilab/geco3

RRID:SCR_018877

biotools: geco3

Operating system(s):  Platform independent

Programming language:  C

Other requirements:  C compiler (e.g., gcc)

License:  GNU GPL

## Availability of Supporting Data and Materials

The supplementary material includes the information to install the benchmark compressors and download and compress the data.

Additional supporting data and materials are available at the *GigaScience* database (GigaDB) [106].

## Additional Files

Supplementary Section 1. Stretching function plot

Supplementary Figure S1. Stretching function applied to the models' probabilities.

Supplementary Section 2. Percentage of symbols guessed correctly

Supplementary Table S1. Percentage of symbols guessed correctly by GeCo2 and GeCo3 for all sequences in dataset four (DS4). The improvement percentage of GeCo3 over GeCo2 is the diff.

Supplementary Section 3. Results for general purpose compressors

Supplementary Table S2. Number of bytes and time needed to represent a DNA sequence for CMIX, DeepZip and ZPAQ. CMIX and DeepZip were run with the default configuration and ZPAQ was run with level 5. Some tests were not run (NR) due to time constraints and DeepZip forced the computer to reboot (SF) with some sequences.

Supplementary Section 4. Complete results for referential compression

Supplementary Table S3. Pairwise referential compression ratio and speed in kB/s for PT sequence using HS as reference. GeCo3 uses 64 hidden nodes and has 0.03 learning rate. The configuration for GeCo2-r and GeCo3-r is “-rm 20:500:1:35:0.95/3:100:0.95 -rm 13:200:1:1:0.95/0:0:0 -rm 10:10:0:0:0.95/0:0:0". For GeCo2-h and GeCo3-h the following models where added “-tm 4:1:0:1:0.9/0:0:0 -tm 17:100:1:10:0.95/2:20:0.95". iDoComp, GDC2 and HRCM use the default configuration.

Supplementary Table S4. Pairwise referential compression ratio and speed in kB/s for PA sequence using HS as reference. Same configurations as in Table S3.

Supplementary Table S5. Pairwise referential compression ratio and speed in kB/s for GG sequence using HS as reference. Same configurations as in Table S3.

Supplementary Table S6. Pairwise referential compression ratio and speed in kB/s for HS sequence using GG as reference. Same configurations as in Table S3.

Supplementary Table S7. Total referential compression ratio and speed in kB/s for a re-sequenced Korean human genome. GeCo3 uses 64 hidden nodes and has 0.03 learning rate. The configuration for GeCo2-r and GeCo3-r (relative approach) is “-rm 20:500:1:35:0.95/3:100:0.95 -rm 13:200:1:1:0.95/0:0:0 -rm 10:10:0:0:0.95/0:0:0". For GeCo2-h and GeCo3-h (conditional approach) the following models where added “-tm 4:1:0:1:0.9/0:0:0 -tm 17:100:1:10:0.95/2:20:0.95". iDoComp, GDC2 and HRCM use the default configuration.

Supplementary Section 5. Referential complexity profiles

Supplementary Figure S2. Smoothed number of bits per symbol (Bps) of GeCo2 subtracted by GeCo3 Bps. The Bps were obtained by referential compression of PT_21 and GG_22, with the same parameters as in Table S3. Places where the line rises above zero indicate that GeCo3 has better compression than GeCo2.

Supplementary Section 6. Referential hidden nodes effect

Supplementary Figure S3. Effect of the number of hidden nodes in reference compressed sequence size and time.

Supplementary Section 7. Referential histograms

Supplementary Figure S4. Histograms for GeCo2 and GeCo3 with the vertical axis in a log 10 scale.

Supplementary Section 8. Reproducibility

## Abbreviations

Bps: bits per symbol; CPU: central processing unit; RAM: random access memory; ReLu: rectified linear unit.

## Competing Interests

The authors declare that they have no competing interests.

## Funding

This work is partially funded by the Portuguese national funds through the FCT in the context of the project UIDB/00127/2020. D.P. is funded by national funds through FCT - Fundação para a Ciência e a Tecnologia, I.P., under the Scientific Employment Stimulus - Institutional Call - CI-CTTI-94-ARH/2019.

## Authors' Contributions

All authors conceived and designed the experiments; M.S. implemented the algorithm and performed the experiments; and all authors analyzed the data and wrote the manuscript.

## Supplementary Material

giaa119_GIGA-D-20-00154_Original_Submission

giaa119_GIGA-D-20-00154_Revision_1

giaa119_GIGA-D-20-00154_Revision_2

giaa119_Response_to_Reviewer_Comments_Original_Submission

giaa119_Response_to_Reviewer_Comments_Revision_1

giaa119_Reviewer_1_Report_Original_Submission Kirill Kryukov, Ph.D. -- 6/13/2020 Reviewed

giaa119_Reviewer_1_Report_Revision_1 Kirill Kryukov, Ph.D. -- 8/30/2020 Reviewed

giaa119_Reviewer_2_Report_Original_Submission Mikel Hernaez -- 7/3/2020 Reviewed

giaa119_Reviewer_2_Report_Revision_1 Mikel Hernaez -- 9/21/2020 Reviewed

giaa119_Supplemental_File
