# Supplementary material for: Efficient DNA sequence compression with neural networks
Source: Gigascience. 2020 Nov 11;9(11):giaa119. doi: 10.1093/gigascience/giaa119 (PMC7657843; doi:10.1093/gigascience/giaa119)
Supplement: giaa119_GIGA-D-20-00154_Original_Submission [file giaa119_giga-d-20-00154_original_submission.pdf]

# GigaScience

## Efficient DNA sequence compression with neural networks

--Manuscript Draft--

| | |
|---|---|
| **Manuscript Number:** | GIGA-D-20-00154 |
| **Full Title:** | Efficient DNA sequence compression with neural networks |
| **Article Type:** | Technical Note |
| **Funding Information:** | Fundação para a Ciência e a Tecnologia (PT) (CI-CTTI-94-ARH/2019) — Mr Diogo Pratas |
| **Abstract:** | Background: The increasing production of genomic data has led to an intensified need for models that can cope efficiently with the lossless compression of DNA sequences. Important applications include long-term storage and compression-based data analysis.<br>In the literature, only a few recent articles propose the use of neural networks for DNA sequence compression. However, they fall short when compared with specific DNA compression tools, such as GeCo2. This limitation is due to the absence of models specifically designed for DNA sequences.<br>In this work, we combine the power of neural networks with specific DNA models. For this purpose, we created GeCo3, a new genomic sequence compressor that uses neural networks for mixing multiple context and substitution tolerant context models.<br><br>Findings: We benchmark GeCo3 as a reference-free DNA compressor in four datasets, including a balanced and comprehensive dataset of DNA sequences, the Y-chromosome, the whole reference human genome, and a compilation of archaeal genomes. GeCo3 achieves a solid improvement in compression over the previous version (GeCo2) of 2.4%, 6%, 4%, and 5.9%, respectively.<br>As a reference-based DNA compressor, we benchmark GeCo3 in four datasets constituted by the pairwise compression of the chromosomes of the genomes of several primates. GeCo3 improves the compression in 12.4%, 11.7%, 10.8% and 10.1% over the state-of-the-art. The cost of this compression improvement is some additional computational time (2.4× slower that GeCo2). The RAM is constant, and the tool scales efficiently, independently from the sequence size. Overall, these values outperform the state-of-the-art.<br><br>Conclusions: GeCo3 is a genomic sequence compressor with a neural network mixing approach, that provides additional gains over top specific genomic compressors. The proposed mixing method is portable, requiring only the probabilities of the models as inputs, providing easy adaptation to other data compressors or compression-based data analysis tools. GeCo3 is released under GPLv3 and is available for free download at https://github.com/cobilab/geco3. |
| **Corresponding Author:** | Milton Silva<br>Universidade de Aveiro Departamento de Electronica Telecomunicacoes e Informatica<br>Aveiro, PORTUGAL |
| **Corresponding Author Secondary Information:** | |
| **Corresponding Author's Institution:** | Universidade de Aveiro Departamento de Electronica Telecomunicacoes e Informatica |
| **Corresponding Author's Secondary Institution:** | |
| **First Author:** | Milton Silva |
| **First Author Secondary Information:** | |
| **Order of Authors:** | Milton Silva<br>Diogo Pratas<br>Armando Pinho |

Powered by Editorial Manager® and ProduXion Manager® from Aries Systems Corporation

| Order of Authors Secondary Information: | |
|---|---|
| Additional Information: | |
| Question | Response |
| Are you submitting this manuscript to a special series or article collection? | No |
| **Experimental design and statistics**<br><br>Full details of the experimental design and statistical methods used should be given in the Methods section, as detailed in our Minimum Standards Reporting Checklist. Information essential to interpreting the data presented should be made available in the figure legends.<br><br>Have you included all the information requested in your manuscript? | Yes |
| **Resources**<br><br>A description of all resources used, including antibodies, cell lines, animals and software tools, with enough information to allow them to be uniquely identified, should be included in the Methods section. Authors are strongly encouraged to cite Research Resource Identifiers (RRIDs) for antibodies, model organisms and tools, where possible.<br><br>Have you included the information requested as detailed in our Minimum Standards Reporting Checklist? | Yes |
| **Availability of data and materials**<br><br>All datasets and code on which the conclusions of the paper rely must be either included in your submission or deposited in publicly available repositories (where available and ethically appropriate), referencing such data using a unique identifier in the references and in the "Availability of Data and Materials" section of your manuscript. | Yes |

*Powered by Editorial Manager® and ProduXion Manager® from Aries Systems Corporation*

| | |
|---|---|
| Have you have met the above requirement as detailed in our Minimum Standards Reporting Checklist? | |

Manuscript

Click here to download Manuscript Efficient_DNA_sequence_compression_with_Neural_Networks.

*GigaScience*, 2017, 1–12

doi: xx.xxxx/xxxx
**Manuscript in Preparation**
**Technical Note**

TECHNICAL NOTE

# Efficient DNA sequence compression with neural networks

Milton Silva[1,2,*], Diogo Pratas[1,2,3,*] and Armando J. Pinho[1,2,*]

[1]Institute of Electronics and Informatics Engineering of Aveiro, University of Aveiro, Portugal and [2]Department of Electronics Telecommunications and Informatics, University of Aveiro, Portugal and [3]Department of Virology, University of Helsinki, Finland

[*]teixeirasilva@ua.pt; pratas@ua.pt; ap@ua.pt

## Abstract

**Background:** The increasing production of genomic data has led to an intensified need for models that can cope efficiently with the lossless compression of DNA sequences. Important applications include long-term storage and compression-based data analysis. In the literature, only a few recent articles propose the use of neural networks for DNA sequence compression. However, they fall short when compared with specific DNA compression tools, such as GeCo2. This limitation is due to the absence of models specifically designed for DNA sequences. In this work, we combine the power of neural networks with specific DNA models. For this purpose, we created GeCo3, a new genomic sequence compressor that uses neural networks for mixing multiple context and substitution tolerant context models. **Findings:** We benchmark GeCo3 as a reference-free DNA compressor in four datasets, including a balanced and comprehensive dataset of DNA sequences, the Y-chromosome, the whole reference human genome, and a compilation of archaeal genomes. GeCo3 achieves a solid improvement in compression over the previous version (GeCo2) of 2.4%, 6%, 4%, and 5.9%, respectively. As a reference-based DNA compressor, we benchmark GeCo3 in four datasets constituted by the pairwise compression of the chromosomes of the genomes of several primates. GeCo3 improves the compression in 12.4%, 11.7%, 10.8% and 10.1% over the state-of-the-art. The cost of this compression improvement is some additional computational time (2.4× slower that GeCo2). The RAM is constant, and the tool scales efficiently, independently from the sequence size. Overall, these values outperform the state-of-the-art. **Conclusions:** GeCo3 is a genomic sequence compressor with a neural network mixing approach, that provides additional gains over top specific genomic compressors. The proposed mixing method is portable, requiring only the probabilities of the models as inputs, providing easy adaptation to other data compressors or compression-based data analysis tools. GeCo3 is released under GPLv3 and is available for free download at https://github.com/cobilab/geco3.

**Key words**: Lossless data compression, DNA sequence compression, Context mixing, Neural networks, Mixture of experts

## Introduction

When using multiple models for prediction, a key problem is how to combine those predictions. An area where this type of problem occurs is lossless data compression [1], with an increasing focus on genomics, given its continuous production and significance.

DNA sequencing rate is increasing exponentially, stretching the genomics storage to unprecedented dimensions. In particular, the projections expose that by the year 2025, between 2 to 40 exabytes per year of additional storage will be needed [2]. Discarding a fraction of the data is not an alternative, given its high importance in many contexts, specifically in biomedical (e.g., personalized medicine) and anthropological fields.

The representation of genomic data usually consists of DNA sequences accompanied by additional channels, such as headers, quality-scores, variant positions, among others, that vary from type and purpose. Despite their conjoint presence, which

**Compiled on:** May 26, 2020.
Draft manuscript prepared by the author.

produces many file types and their respective data compression tools, the core of the data are the DNA sequences.

Although data compression is the natural approach for decreasing the storage of DNA sequences losslessly [3], it can also be efficiently applied to sequence analysis and prediction using specialized-purpose compressors [4, 5, 6]. Specialized DNA compressors achieve substantially higher compression than general-purpose [7]. The majority of these compressors use various models to predict the next symbol and combine their predictions in order to achieve efficient results [7, 8].

However, the efficient combination of multiple models for DNA sequence compression is not a trivial problem given the heterogeneity and non-stationarity of the data. In this paper, we address the problem of combining the predictions of different models to produce an improved predictive model and, by consequence, improve the compression of DNA sequences. To solve the problem, we take GeCo2 [8], a specialized genomic compressor that uses a mixture of context and substitution tolerant context models [9]. GeCo2 combines the predictions of the models, using an algebraic combiner, where weights are attributed to each model and updated based on the model performance, with a specific forgetting factor for each.

In machine learning, the area that deals with these types of problems is designated as ensemble methods, also known as a mixture of experts [10]. Ensemble methods concern themselves both by building appropriate models and combining their predictions. The main idea presented in this paper is to substantially improve the mixing of experts of a previous method (GeCo2) with a stacked generalization approach [11]. Stacked generalization uses a meta-model that takes as inputs the outputs of other models and is trained to learn the mapping between the models' outputs and the actual correct outputs. To implement stack generalization, we use a multilayer perceptron. The neural network takes as inputs the probabilities of each model as well as the derived features [12] that represent past model performance. The network then outputs the probabilities for each symbol.

To evaluate this approach, we created a new DNA compression tool, GeCo3, and benchmark it both in reference-free and referential compression. Four datasets are selected for reference-free compression benchmark containing different sequence nature, lengths, and redundancy levels. For benchmarking the reference-based approach, another four datasets were chosen that include the pairwise compression of the whole chromosome sequences of several primates.

The results of the benchmarks show a consistent improvement in the compression ratio of GeCo3 over state-of-the-art DNA compressors, both in reference-free and reference approaches, enabling the use of GeCo3 as a long-term storage tool and to substantially increase the precision and accuracy of compression-based data analysis tools. Additionally, we provide an easy-to-use exportable code to integrate into other data compression or data analysis tools.

In the following subsection, we provide a background on the DNA sequence compression area, both at reference-free and reference-based compression. Then, we describe GeCo3 in detail, and, finally, we provide the benchmark results.

## DNA sequence compression

The collection of DNA diversity has shown the existence and co-existence of micro and macro genomes in the most diverse places, for example, in extreme environments as uranium mines [13], in soft and hard tissues [14, 15], ancient cadavers [16], marine environments [17], or deep subterranean habitats [18]. The environment and species interactions are a key for genome adaptation, providing a wide diversity in genome characteristics, namely high copy number, high heterogeneity, high level of substitution mutations, or multiple rearrangements, such as fissions, fusions, translocations, or inverted repeats [19, 20]. Additionally, since genomic (DNA) sequences are an output of biochemical and computational methods, these sequences may have other alteration sources, for example contamination [21], environmental factors [22, 23], pathogenic species included in the samples [24, 25], and unknown sources [26]. Therefore, representing genomic sequences requires the ability to model heterogeneous, dynamic, incomplete, and imperfect information [27].

The above specific characteristics led to the development of the field which studies and constructs specific genomic data compressors [28, 29]. This field has now 27 years and started with Biocompress [30]. After, several algorithms emerged, mostly modeling the existence of exact or approximate repeated and inverted repeated regions, through the usage of simple bit encoding, context modeling, or dictionary approaches [31, 32, 33, 34, 35, 36, 37, 38, 39, 40, 41, 42, 43, 44, 45, 46, 47, 48, 49, 50, 51, 52, 53, 54, 53, 54, 55, 56, 57, 58, 59, 7, 60, 61, 62, 8, 63, 64, 65].

The FASTA format development permitted to standardize the co-existence of DNA sequences (in a visible horizontal range) along with annotations (headers). Usually, the DNA sequence is substantially the most abundant part of this data and, hence, multiple tools use specialized DNA compression algorithms combined with simple header coding, namely Deliminate [66], MFCompress [67], and NAF [68]. Notwithstanding, for comparison purposes with DNA sequence compressors, setting a minimal header, asymptotically, increases its irrelevance relative to the DNA sequence according to its size.

From all the previous algorithms, the most efficient according to compression ratio in the wide diversity of DNA sequences are XM [44], GeCo2 [8], and Jarvis [65]. These compressors apply statistical and algorithmic models mixtures combined with further arithmetic encoding. Specifically, the XM algorithm [44] combines three types of experts, namely repeat models, a low-order context model, and a short memory context model of 512 bytes. The GeCo2 algorithm [8] uses soft-blending cooperation between context models and substitution tolerant context models [9] with a specific forgetting factor for each model. The Jarvis compressor [65] uses a competitive prediction model to estimate, for each symbol, the best class of models to be used; there are two classes of models: weighted context models and weighted stochastic repeat models, where both classes of models use specific sub-programs to handle inverted repeats efficiently.

Some compressors use a reference genome as an additional input. This approach is called referential compression, and it started to gain momentum in 2009 [69, 70]. Referential compressors attained substantially higher compression ratios compared to reference-free compressors. The resulting compressed lengths can be hundreds or thousands of times smaller than the original file [71, 72]. As an example, an entire human genome of about 3GB can be compressed to 4MB by referential compression; on the other hand, a reference-free compressor minimizes the data to 580MB, approximately. The majority of reference-based compression algorithms use dictionaries, repeats models, or context models [69, 70, 73, 74, 75, 76, 77, 55, 78, 71, 72, 79, 80, 8, 81]. From the previous compressors, the most productive, according to compression ratio, are HiRGC [79], GeCo2 [8], iDoComp [71], GDC2 [72] and HRCM [81]. The HiRGC [79] is based on a 2-bit encoding scheme and an advanced greedy-matching search on a hash table. The GeCo2 [8] is described above. The iDoComp [71] uses a suffix array for loading the reference and later applies a greedy parsing of the target that benefits the substitutional single nucleotide polymorphisms that occur in higher number. The GDC2 [72] per-

forms a Ziv-Lempel factoring combined with a second-level factoring and followed by arithmetic coding. The HRCM [81] explores sequence information extraction, followed by sequence information matching and further encoding.

As a general-purpose sequence compressor, the idea of using neural networks to mix probabilities is seen in [1]. In this case, it is called logistic mixing. Logistic mixing can be viewed as using a neural network without hidden layers and a simpler update rule than backpropagation. Other general-purpose followed the same line, namely Cmix [82], DeepZip [83], and DeepDNA [63]. The Cmix [82] uses recurrent neural networks trained with stochastic gradient descent for context mixing. The DeepZip [83] also uses recurrent neural networks, both as predictors (models) and as mixers. The DeepDNA [63] uses a hybrid approach with a convolutional layer to capture the genome's local features and a recurrent layer to model long-term dependencies.

Although the best general-purpose compressors use complex computational models, namely based on neural networks, it has been shown that they still have lower compression capabilities (5-10%) using substantially higher computational time according to the most efficient specific compressors [83]. The discrepancy of precision is higher when the method is designed for fast computations [84]. The main reason that the best general-purpose algorithms (using neural networks) are not so efficient is that they do not use specific DNA models that take into account the algorithmic nature of genomic sequences, harming the model sensitivity.

In this paper, we combine the sensitivity of specific DNA models, namely the usage of multiple context models combined with DNA specific algorithmic models, with the power of neural networks for context mixing.

## Methods

In this section, we present the methods that describe the proposed compressor (GeCo3). GeCo3 uses a combination of multiple context models and substitution tolerant context models of several order-depths. The neural network provides an efficient combination of these models. Therefore, we describe the new method with the main focus on the neural network, including the inputs, updates, outputs, and training process.

### Neural network structure

The model mixing is constructed using a feed-forward artificial neural network trained with stochastic gradient descent [85]. This choice is motivated by implementation simplicity and competitive performance compared to more complex neural networks [86]. The activation function for this network is the sigmoid, and the loss function is the mean squared error. The network structure is fully connected with one hidden layer, as seen in Fig. 1b. One bias neuron is used for the input and hidden layer, while the weights respect the Xavier initialization according to [87]. Although we empirically tested different activation functions (ReLu, TanH) and a higher number of hidden layers, the most efficient structure was obtained with the previous description.

We introduced two parameters for the GeCo3 compression tool in order to control the number of nodes of the hidden layer and the learning rate. These parameters are written in the compressed file header to ensure a lossless decompression.

### Neural network inputs

The stretched probabilities of each symbol are used as inputs to the network. These are given by

$$p_{i,j} = stretch\left(\frac{1+f_{i,j}}{\sum_{m\in\Theta} 1+f_{i,m}}\right) - stretch\left(mean_p\right), \tag{1}$$

where $f_{i,j}$ is the frequency of symbol $j$ for model $i$ with $\Theta$ as the set of all symbol and $mean_p$ is the mean probability of each symbol.

We stretch the probabilities according to the work of Mahoney [1]. The effects of stretching can be seen in Supplementary Section 1 (Stretching function plot). The inputs are normalized for forcing the average to be close to zero by subtracting the stretched mean probability, which, for the case of DNA, we assume to be 0.25. The normalization and its motivation are explained in [88]. Stretching the probabilities has the effect of scaling them in a non-linear way, which increases the weights of probabilities near zero and one.

The context models, substitution tolerant context models, and the mixed probabilities of GeCo2 are used as input models. This inclusion means that the mixing done in GeCo2 is not discarded, but are used as an additional input to the neural network.

We extract features from the context (the last $n$ symbols) and also calculate model and network performance indicators to improve the network predictions. These are used as inputs to the neural network. Three performance indicators are derived for each mode according to the names *hit*, *best*, and *bits*. These features correspond to three input nodes per model, as seen in Fig. 1b.

To measure how precise model $i$ is voting, we use

$$hit_{i,n} = \begin{cases} hit_{i,n-1}, & \text{if } \forall x,y \in \Theta : p_{i,x} = p_{i,y} \\ hit_{i,n-1} + 0.1, & \text{if } \forall x \in \Theta : p_{i,sym} > p_{i,x} \\ hit_{i,n-1} - 0.1, & \text{otherwise.} \end{cases} \tag{2}$$

The symbol with the highest probability is considered the vote of the model. Each time the model votes correctly, *hit* is increased. If the model abstains (probabilities of each symbol are equal), then *hit* remains the same; otherwise, it decreases.

For each model, we also measure if it has assigned the highest probability to the correct symbol, compared to all other models. This is given by

$$best_{i,n} = \begin{cases} best_{i,n-1}, & \text{if } \forall x,y \in \Theta : p_{i,x} = p_{i,y} \\ best_{i,n-1} + 0.1, & \text{if } p_{i,sym} \geq p_{k,sym} \\ best_{i,n-1} - 0.1, & \text{otherwise.} \end{cases} \tag{3}$$

The update rules for *best* are similar to *hit* and both have a domain of $[-1, 1]$.

As an approximation to the average number of bits the model would output, we use an exponential moving average

$$bits_{i,n} = \alpha_1 \cdot (-\log_2(p_{i,sym}) + \log_2(mean_p)) + (1-\alpha_1)\cdot bits_{i,n-1}, \tag{4}$$

with $\alpha_1 = 0.15$. This input is also normalized such that the average value is close to zero.

In Eqs. (2), (3) and (4), $p_{i,sym}$ is the probability assigned by model $i$ to the actual symbol in the sequence. To reach these features and their constants, we tested each with a couple of files from one dataset and adjusted until finding a value that produced satisfactory results.

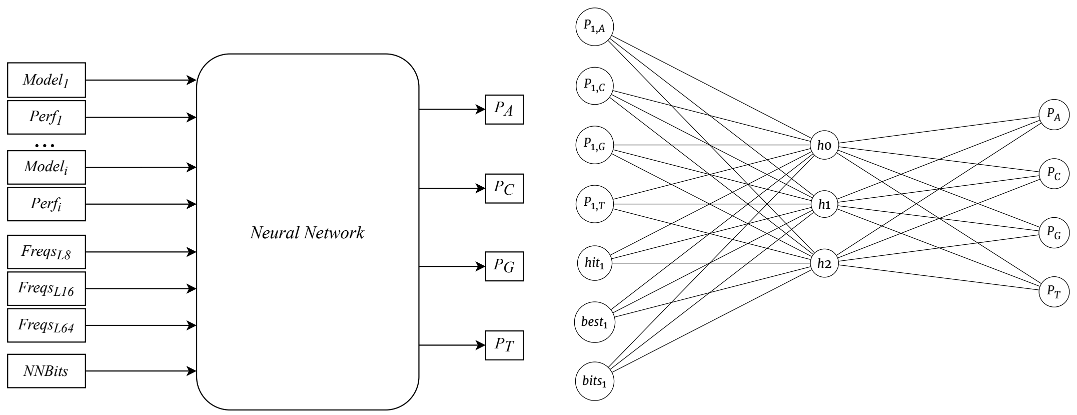


**(a)** High level architecture

**(b)** Neural network detail

**Figure 1.** Mixer architecture: (a) High level overview of inputs to the neural network (mixer) used in GeCo3. *Model*$_1$ through *Model*$_i$ represent the GeCo2 model outputs (probabilities for A, C, T, G). *Perf* represents the performance metrics (*hit*, *best*, *bits*) for each model. *Freqs* are the frequencies for the last 8, 16, and 64 symbols. *NNBits* is a moving average of the approximate number of bits that the neural network is producing. The network outputs represent the non-normalized probabilities for each DNA symbol. (b) A fully connected neural network with one hidden layer. For illustration purposes, this neural network only has the inputs corresponding to one model and the three features that evaluate the model performance. The frequencies of the last 8, 16, and 64 symbols, as well as the *NNBits* and the bias neurons, are omitted.

The features extracted from the context are the probabilities of each symbol for the last 8, 16, and 64 symbols. These represent a total of twelve input nodes. In Fig. 1a, these nodes are represented by $Freqs_{L8}$, $Freqs_{L16}$ and $Freqs_{L64}$. For example, to obtain the probabilities for the last eight symbols with the sequence ACAGTAAA, the number of A's is divided by the number of total symbols, so the frequency of symbol A is 5/8 and for the other symbols is 1/8. These probabilities are then scaled to fit between -1 and 1.

In Fig. 1a, *NNBits* represents the exponential moving average of the approximate number of bits and is given by

$$nnbits_n = \alpha_2 \cdot (-\log_2(p_{sym}) + \log_2(mean_p)) + (1 - \alpha_2) \cdot nnbits_{n-1}, \quad (5)$$

with $p_{sym}$ as the probability the network assigned to the correct symbol and $\alpha_2 = 0.5$.

## Updating model performance features

As an example of how to update the features, consider two symbols and three models, and assume all features start equal to zero. Model 1 assigns the probabilities $[0.5, 0.5]$, meaning that the model abstains and, as such, no change is made to *hit* or *best*. Also, $bits_1$ would be equal to zero. The probabilities for model 2 and 3 are $[0.7, 0.3]$ and $[0.8, 0.2]$, respectively. Assuming the models voted correctly, then *hit* is now $0 + 0.1 = 0.1$ for both. Because model 3 assigned the highest probability to the correct symbol then $best_3$ is now $0+0.1 = 0.1$ and $best_2$ remains 0. Moreover, $bits_2$ would become $bits_2 = 0.15 \cdot (-\log_2(0.7) + log_2(0.5))$ and $bits_3 = 0.15 \cdot (-\log_2(0.8) + log_2(0.5))$.

## Neural network outputs and training

One node per symbol is used as output from the network. After the result is transferred to the encoder, the network is trained with the current symbol using the learning rate specified within the program input.

When compared to GeCo2, the results of the new mixing contain two main differences. First, the sum of output nodes is different from one. This outcome is corrected by dividing the node's output by the sum of all nodes. The second difference is that the new approach outputs probabilities in the range $]0, 1[$, while in GeCo2, the mixing always yielded probabilities inside the range of the models.

# Results

In this section, we benchmark GeCo3 against state-of-the-art tools in both reference-free and referential compression approaches. In the following subsection, we describe the datasets and materials used for the benchmark, followed by the comparison with GeCo2 using different characteristics, number of models, and data redundancy. Finally, we provide the full benchmark for the eight datasets.

## Datasets and materials

The benchmark includes eight datasets. Four datasets are selected for reference-free compression, including

- **DS1**: a comprehensive-balanced dataset (proposed in [89]);
- **DS2**: highly repetitive DNA with the human Y-chromosome and a human mitogenome collection (proposed in [90]);
- **DS3**: two compilations of archaeal and viral genomes;
- **DS4**: four whole genomes, including three from primates (human, chimpanzee, and gorilla) and one from the Norway spruce.

On the other hand, to benchmark the reference-based approach, we use the complete genomes of four primates (human, gorilla, chimpanzee, and orangutan) with a pairwise chromosomal compression. Non-human chromosomes are concatenated to match the human chromosomal fusion [91]. For each chromosomal pair, the following compression was performed

- **DSR1**: chimpanzee (PT) using human (HS) as a reference;
- **DSR2**: orangutan (PA) using human (HS) as a reference;
- **DSR3**: gorilla (GG) using human (HS) as a reference;
- **DSR4**: Human (HS) using gorilla (GG) as a reference.

All the materials to replicate the results, including the sequence identifiers, URL, filtering applications, and associated commands, can be found at the Supplementary Section 7 (Reproducibility).

## Neural network mixing compression

In order to assess the performance of the neural network mixing, we compare GeCo2 with GeCo3. To ensure a fairness comparison, the compression modes, including the models and parameters, are kept identical for both programs.

In Table 1, GeCo2 and GeCo3 are compared using the compression modes published in [8]. The overall compression improves by 1.93%, and the average improvement is 1.06%. The larger sequences (larger than ScPo) have average improvements of 2.04%, while the remaining have modest improvements of 0.4%. Only the two smallest sequences show negative improvement, given the absence of enough time to train the network. Additional, the eight bytes that are used to transmit the two network parameters to the decompressor are a significant percentage of the total size, unlike in larger sequences. Overall, GeCo3 improves the compression of the whole dataset to more than 1.9%.

## Neural network mixing computational resources

Regarding computational resources, the mixing modification is 3.4× slower, as shown in Table 1. The computation was performed on an Intel(R) Core(TM) i5-8265U CPU @ 1.60GHz running Linux(5.5.8) with the scaling governor set to performance. The new mixing approach is always slower because GeCo2's mixing is still used, not as a result of the encoder, but rather as an input to the network. The difference in RAM usage of both approaches is less than 1 MB, which corresponds to the size of the neural network and the derived features for each model.

The number of hidden nodes is chosen to fit in the vector registers in order to take full advantage of the vectorized instructions. Accordingly, we set the number of hidden nodes as a multiple of eight, where floating points of four bytes represent the nodes and 32 bytes represent the vector registers.

## Effects of the hidden layer size on mixing

Increasing or decreasing the number of hidden nodes affects the number of weights, and it also affects compression, as can be seen in Fig. 2. Increasing the number of nodes increases the compression up to a point. This point varies from sequence to sequence; however, the abruptest gains in compression generally occur until 24 hidden nodes. As expected, increasing the number of hidden nodes leads to an increase in execution time and a progressive decline of compression gain. These results are also consistent in referential compression as seen in Supplementary Section 5 (Referential hidden nodes effect).

## The importance of derived features on mixing

We removed the derived features from the inputs to the network to assess its impact on the mixing performance. The results are present in Table 2.

When using just the models' probabilities as inputs, the compression is efficient than GeCo2 by a marginal precision (0.18%), while, in the majority of the sequences, there is no improvement. By adding the result of the GeCo2 mixing as an input, the improvement increases to 1.36%. The gain escalates, having an improvement of 1.73%, when using the context models and tolerant context models as inputs and the derived features.

## Scaling the number of models

GeCo2 and GeCo3 contain several modes (compression levels), which are parameterized combinations of models with diverse neural network characteristics. To see how the compression of the new approach scales with more models, we introduced mode 16 with a total of 21 models. This new mode was used to compress the sequences of HoSa to HePy (by size order). For the remaining sequences, the same models were used as in Table 1. We used this approach because increasing the number of models was incapable of improving the compression of GeCo3 and GeCo2, given the smaller dimensions of these datasets. The number of hidden nodes was also adjusted until no tangible improvements in compression were observed.

The results in Table 3 show that the distance between the approaches increases from 1.93% to 2.43%. The time difference reduces from 3.4× to 2.4×. This reduction is due to the increased percentage of time spent by the higher-order con-

**Table 1.** Number of bytes needed to represent each DNA sequence for GeCo2 and GeCo3 compressors. The column mode applies to both compression methods, while the learning rate and the number of hidden nodes only apply to the latter.

| ID | GeCo2 bytes | GeCo3 bytes | GeCo2 secs | GeCo3 secs | Mode | L.Rate | H.Nodes |
|---|---|---|---|---|---|---|---|
| HoSa | 38,845,642 | **37,892,426** | 188.8 | 628.4 | 12 | 0.03 | 64 |
| GaGa | 33,877,671 | **33,411,627** | 135.0 | 448.2 | 11 | 0.03 | 64 |
| DaRe | 11,488,819 | **11,189,707** | 53.0 | 200.7 | 10 | 0.03 | 64 |
| OrSa | 8,646,543 | **8,434,883** | 37.5 | 138.2 | 10 | 0.03 | 64 |
| DrMe | 7,481,093 | **7,379,990** | 28.1 | 102.9 | 10 | 0.03 | 64 |
| EnIn | 5,170,889 | **5,066,676** | 22.8 | 77.5 | 9 | 0.05 | 64 |
| ScPo | 2,518,963 | **2,511,054** | 10.1 | 26.3 | 8 | 0.03 | 40 |
| PlFa | 1,925,726 | **1,906,919** | 9.3 | 24.1 | 7 | 0.03 | 40 |
| EsCo | 1,098,552 | **1,094,298** | 1.9 | 8.6 | 6 | 0.03 | 40 |
| HaHi | 902,831 | **896,037** | 1.6 | 7.0 | 5 | 0.04 | 40 |
| AeCa | 380,115 | **377,343** | 0.8 | 2.2 | 5 | 0.04 | 16 |
| HePy | 375,481 | **373,583** | 0.8 | 3.0 | 4 | 0.04 | 40 |
| YeMi | 16,798 | **16,793** | 0.1 | 0.1 | 3 | 0.09 | 24 |
| AgPh | **10,708** | 10,715 | 0.0 | 0.1 | 2 | 0.06 | 16 |
| BuEb | **4,686** | **4,686** | 0.0 | 0.0 | 1 | 0.06 | 8 |
| Total | 112,744,517 | **110,566,737** | 489.7 | 1667.3 | | | |

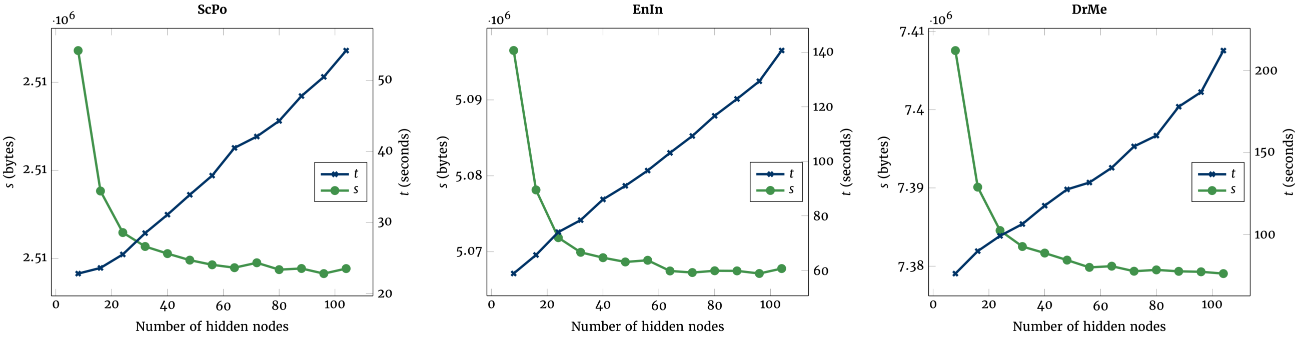


**Figure 2.** Number of bytes (*s*) and time (*t*) according to the number of hidden nodes for the reference-free compression of ScPo, EnIn, and DrMe sequence genomes.

**Table 2.** Number of bytes needed to represent each DNA sequence using the GeCo3 compressor with specific conditions. For the column named Models, only the context models and tolerant context models of GeCo2 were used as network inputs. For "Models + GeCo2", the result of GeCo2 mixing was also used as input. With "Models + Derived" the inputs for the network were the same as "Models" with the derived features added. The compression modes are the same as in Table 1.

| ID | Models | Models + GeCo2 | Models + Derived |
|---|---|---|---|
| HoSa | 38,556,039 | 38,153,358 | **37,943,933** |
| GaGa | 33,758,606 | 33,548,929 | **33,444,816** |
| DaRe | 11,615,937 | 11,280,688 | **11,251,390** |
| OrSa | 8,694,790 | 8,517,947 | **8,471,715** |
| DrMe | 7,475,341 | 7,414,919 | **7,392,290** |
| EnIn | 5,183,237 | 5,095,391 | **5,087,359** |
| ScPo | 2,524,818 | 2,514,188 | **2,513,085** |
| PlFa | 1,928,282 | 1,912,745 | **1,912,176** |
| EsCo | 1,104,646 | 1,095,589 | **1,096,255** |
| HaHi | 903,019 | 898,280 | **898,145** |
| AeCa | 378,226 | 377,857 | **377,696** |
| HePy | 379,285 | **374,364** | 374,975 |
| YeMi | 16,901 | **16,827** | 16,882 |
| AgPh | 10,744 | **10,727** | 10,731 |
| BuEb | **4,694** | 4,696 | 4,698 |
| Total | 112,534,565 | 111,216,505 | 110,796,146 |

text models. These results show that neural network mixing can scale with the number of models. The forgetting factors for this new mode were not tuned, due to the use of a large number of models. Therefore, with this tuning, additional gains can be observed. Nevertheless, this shows another advantage of this new mixing, which is that there are only two parameters that need tuning regardless of the number of models. As the sequence size and the number of models increases, there is almost no tuning required, with the optimal values being around 0.03 for the learning rate and 64 hidden nodes.

## Compressing highly repetitive and large sequences

In this subsection, we show how the reference-free compression scales with the new mixing using highly repetitive and extensive sequences, namely in the gigabyte scale. Three datasets are selected, and the results shown in Table 3.

According to the results from Table 3, GeCo3 compresses the highly repetitive sequences (DS2 and DS3) with an average of 6.4% compared to GeCo2 using more 2.2× time. For the larger sequences of DS1, GeCo3 has an average compression improvement of 3.2% in the primates, and 8.2% in the spruce (PiAbC), with a 2.8× slower execution time. The spruce (PiAbC) is the longest sequence of the dataset and exactly where GeCo3 presents the highest gain. These results suggest that the compression of longer repetitive sequences may present even higher compression gains.

## Reference-free sequence compression bechmark

In this subsection, we compare GeCo3 with other specialized reference-free compressors, namely XM (v3.0) [44], GeCo2 (previously compared), Jarvis [65], and NAF [68]. As presented in Table 3, GeCo3 achieves the best total size in three out of four datasets. Only in one dataset (DS3), GeCo3 was unable to achieve the best compression, delivered by Jarvis. The type of dataset justifies this performance. Specifically, DS3 contains a high number of identical sequences with a length near 16.5k. This dataset is a collection of mitogenomes where the variability is very low, which gives an advantage to models of extremely repetitive nature. Such models, also known as weighted stochastic repeat models, are present in Jarvis, unlike in GeCo3. The reason why we excluded the inclusion of these models in GeCo is that they fail in scalability because the RAM increases according to the sequence length. For larger datasets than DS3, namely DS1 and DS2, Jarvis was unable to compress given RAM substantially higher than 15 GB. On the other hand, GeCo3 has constant RAM, which is not affected by the sequence length but rather only in the mode used.

Comparing GeCo3 against the second best compressor for each dataset, the compression gain is 1.9% (vs Jarvis), −3.2% (vs Jarvis), 4.7% (vs XM) and 5.9% (vs GeCo2) for DS1, DS2, DS3 and DS4, respectively. For the individual sequences in the datasets, GeCo3 compresses more than the other compressors, except for the AgPh, BuEb, and Mito. Tiny sequences compose the AgPh and BuEb dataset, and the neural network has not enough time to learn, while Mito has already been mentioned above.

Regarding computational time, GeCo3 is faster than XM per dataset, spending on the average only half the time. Against GeCo2, it is slower 2.4× on average, and compared to Jarvis, it is 5.5× slower on DS4, but only 1.1× in DS3. NAF is the fastest compressor in the benchmark. Compared to NAF, GeCo3 is between 14.2× slower for DS4 and 4× for DS1.

Regarding computational memory, the maximum amount of RAM used for GeCo2 and GeCo3 was 12.6GB, Jarvis peaked at 15GB, XM at 8GB, and NAF used at most 0.7GB. Jarvis could not complete the compression for DS1 and DS2 due to a lack of memory. This issue is a limitation that was earlier mentioned above. We also note that the XM is unable to decompress some of the sequences. In these cases, the decompressed file has the correct size, but the sequence does not fully match the original file. NAF, GeCo2, and GeCo3 were the only compressors

**Table 3.** Number of bytes and time needed to represent a DNA sequence for NAF, XM, Jarvis, GeCo2, and GeCo3. For the sequences HoSa to BuEb, Jarvis uses the same configuration as in [65], for DS3 it uses level 7. XM uses the default configuration. NAF uses the highest compression level (22). GeCo2 and GeCo3 use mode 16 for DS4 except for the three smaller files(YeMi, AgPh, BuEb), which use the same parameters as in Table 1. For DS1 the parameters are "-tm 3:1:1:1:0.70/0:0:0 -tm 8:1:1:1:0.85/0:0:0 -tm 13:10:0:1:0.85/0:0:0 -tm 19:500:1:40:0.85/5:20:0.85". For DS2 and DS3 "-tm 3:1:1:1:0.8/0:0:0 -tm 6:1:1:1:0.85/0:0:0 -tm 9:1:1:1:0.85/0:0:0 -tm 12:10:0:1:0.85/0:0:0 -tm 15:200:1:10:0.85/2:1:0.85 -tm 17:200:1:10:0.85/2:1:0.85 -tm 20:500:1:40:0.85/5:20:0.85". GeCo3 uses a learning rate of 0.03 and 64 hidden nodes for all but YeMi, AgPh and BuEb. The character '*' indicates the sequence was not compressed due to an error and '/' in due to out of memory. Results where the decompression produces different results than the input file are appended by the character '?'.

| | | NAF | | XM | | Jarvis | | GeCo2 | | GeCo3 | |
|---|---|---|---|---|---|---|---|---|---|---|---|
| DS | ID | size | time | size | time | size | time | size | time | size | time |
| 1 | PiAbC | 2.28 GB | 2h16m08s | * | * | / | / | 1.86 GB | 3h22m54s | **1.71 GB** | 9h16m57s |
| | HoSaC | 635.39 MB | 33m46s | * | * | / | / | 580.40 MB | 47m25s | **561.54 MB** | 2h19m41s |
| | PaTrC | 619.50 MB | 33m47s | * | * | / | / | 569.40 MB | 45m40s | **551.51 MB** | 2h15m41s |
| | GoGoC | 603.37 MB | 33m34s | * | * | / | / | 556.54 MB | 44m26s | **539.31 MB** | 2h02m06s |
| | Total | 4.14 GB | 3h57m17s | * | * | / | / | 3.57 GB | 5h40m27s | **3.36 GB** | 15h54m26s |
| 2 | Archaea | 128.09 MB | 6m22s | 103.01 MB? | 1h56m01s | / | / | 103.70 MB | 24m58s | **97.86 MB** | 53m08s |
| | Virus | 85.51 MB | 6m04s | 63.93 MB? | 1h54m14s | / | / | 65.63 MB | 24m34s | **61.23 MB** | 53m26s |
| | Total | 213.60 MB | 12m27s | 166.93 MB? | 3h50m15s | / | / | 169.34 MB | 49m32s | **159.09 MB** | 1h46m35s |
| 3 | Mito | 35.93 MB | 2m13s | 28.12 MB? | 52m49s | **27.11 MB** | 18m15s | 30.40 MB | 9m04s | 28.17 MB | 20m06s |
| | HoSaY | 5.17 MB | 10s | 3.88 MB? | 3m47s | 3.93 MB | 2m13s | 4.08 MB | 1m03s | **3.85 MB** | 2m16s |
| | Total | 41.10 MB | 2m23s | 32.01 MB? | 56m36s | **31.04 MB** | 20m29s | 34.48 MB | 10m07s | 32.02 MB | 22m23s |
| 4 | HoSa | 41.73 MB | 1m48s | 38.66 MB? | 33m01s | 38.66 MB | 4m52s | 38.79 MB | 9m33s | **37.56 MB** | 23m30s |
| | GaGa | 35.57 MB | 1m23s | 33.83 MB? | 26m22s | 33.70 MB | 2m35s | 33.75 MB | 7m16s | **33.26 MB** | 18m08s |
| | DaRe | 12.83 MB | 28s | 11.17 MB? | 10m22s | 11.17 MB | 1m44s | 11.44 MB | 3m05s | **10.97 MB** | 7m43s |
| | OrSa | 9.53 MB | 19s | 8.48 MB? | 8m03s | 8.45 MB | 1m26s | 8.60 MB | 2m09s | **8.34 MB** | 5m19s |
| | DrMe | 7.85 MB | 13s | 7.53 MB? | 5m50s | 7.49 MB | 23s | 7.47 MB | 1m43s | **7.36 MB** | 4m12s |
| | EnIn | 5.87 MB | 11s | 5.12 MB? | 3m57s | 5.09 MB | 41s | 5.14 MB | 1m21s | **5.02 MB** | 3m17s |
| | ScPo | 2.59 MB | 4s | 2.53 MB | 1m05s | 2.52 MB | 10s | 2.52 MB | 38s | **2.51 MB** | 1m23s |
| | PlFa | 2.02 MB | 3s | 1.92 MB | 1m10s | 1.92 MB | 9s | 1.93 MB | 34s | **1.90 MB** | 1m11s |
| | EsCo | 1.15 MB | 2s | 1.11 MB | 20s | 1.10 MB | 4s | 1.10 MB | 21s | **1.09 MB** | 41s |
| | HaHi | 948.69 KB | 1s | 914.87 KB | 20s | 899.46 KB | 2s | 899.17 KB | 19s | **889.51 KB** | 35s |
| | AeCa | 396.82 KB | 1s | 387.00 KB | 3s | 380.51 KB | 0s | 381.29 KB | 13s | **376.97 KB** | 19s |
| | HePy | 404.55 KB | 1s | 384.30 KB | 5s | 374.36 KB | 0s | 375.66 KB | 13s | **371.62 KB** | 20s |
| | YeMi | 17.35 KB | 1s | 16.84 KB | 0s | 16.86 KB | 0s | 16.80 KB | 0s | **16.79 KB** | 0s |
| | AgPh | 11.02 KB | 1s | 10.71 KB | 0s | 10.75 KB | 0s | **10.71 KB** | 0s | 10.72 KB | 0s |
| | BuEb | 4.81 KB | 1s | **4.64 KB** | 0s | 4.69 KB | 0s | 4.69 KB | 0s | 4.69 KB | 0s |
| | Total | 120.94 MB | 4m42s | 112.07 MB | 1h30m44s | 111.79 MB | 12m13s | 112.42 MB | 27m31s | **109.68 MB** | 1h06m42s |

that have been able to compress all the sequences losslessly, independently from the size. The overall results of these compressors show and improvement of the compression of GeCo3 of 18, 9% and 5.8% over NAF and GeCo2, respectively.

## Reference-based sequence compression bechmark

In this subsection, we benchmark GeCo3 with the state-of-the-art referential compressors. The comparison benefits the compression between the genomes of different species and not re-sequencing. Resequencing is applied to the same species and, in a general case, limits the domain of applications; for example, phylogenomic, phylogenetic, or evolutionary analysis.

To run the experiments, we used four complete genomes of closely related species: *Homo sapiens* (HS), *Pan troglodytes* (PT), *Gorilla gorilla* (GG) and *Pongo abelii* (PA). The compression for PT, GG, and PA was done using HS as the reference. HS was compressed using GG as a reference. Each chromosome was paired with the corresponding one of the other species. Due to the unavailability of chromosome Y for GG and PA, comparisons that involved these chromosomes were not made. The compressors used in this benchmark are GeCo3, GeCo2, iDoComp [71], GDC2 [72], and HRCM [81]. The FASTA files were filtered such that the resulting file only contained the symbols $\{A, C, G, T\}$, and a tiny header line. HRCM needs the line size to be limited; therefore, line breaks were added for the files under its compression. However, this approach prevents a direct comparison of total compressed size and time, which we solved using the compression ratio percentage ($output\_size \div input\_size \times 100$) and the speed in kilobytes per seconds ($input\_size \div 1000 \div seconds\_spent$). For GeCo2 and GeCo3, two approaches of referential compression are considered. One approach is based on conditional compression, where a hybrid of both reference and target models are used. The other approach, called relative approach, uses exclusively models loaded from the reference sequence. Both types of compression assume causality, which means that with the respective reference sequence, the decompressor is able to decompress without loss. The reason why we benchmark these two approaches is that there are many sequence analysis applications for both approaches.

The comparison is presented in Table 4, showing the

**Table 4.** Total referential compression ratio and speed in kB/s. GeCo3 uses 64 hidden nodes and has 0.03 learning rate. The configuration for GeCo2-r and GeCo3-r (relative approach) is "-rm 20:500:1:35:0.95/3:100:0.95 -rm 13:200:1:1:0.95/0:0:0 -rm 10:10:0:0:0.95/0:0:0". For GeCo2-h and GeCo3-h (conditional approach) the following models where added "-tm 4:1:0:1:0.9/0:0:0 -tm 17:100:1:10:0.95/2:20:0.95". iDoComp, GDC2 and HRCM use the default configuration.

| | | HRCM | | GDC2 | | iDoComp | | GeCo2-r | | GeCo3-r | | GeCo2-h | | GeCo3-h | |
|---|---|---|---|---|---|---|---|---|---|---|---|---|---|---|---|
| DSR | ID | ratio | speed | ratio | speed | ratio | speed | ratio | speed | ratio | speed | ratio | speed | ratio | speed |
| 1 | HSxPT | 6.29 | 2,003 | 5.01 | 931 | 4.78 | 3,941 | 4.16 | 674 | 3.65 | 334 | 4.02 | 462 | **3.52** | 240 |
| 2 | HSxPA | 7.41 | 1,510 | 5.81 | 462 | 5.35 | 6,138 | 4.01 | 666 | 3.50 | 326 | 3.87 | 462 | **3.42** | 238 |
| 3 | HSxGG | 8.80 | 2,040 | 7.06 | 691 | 6.70 | 3,529 | 5.58 | 669 | 4.96 | 329 | 5.43 | 466 | **4.84** | 241 |
| 4 | GGxHS | 8.56 | 2,178 | 7.29 | 756 | 7.00 | 3,570 | 5.68 | 711 | 5.11 | 353 | 5.07 | 469 | **4.53** | 247 |
| | Total | 7.76 | 1,897 | 6.29 | 666 | 5.96 | 4,022 | 4.86 | 680 | 4.31 | 335 | 4.59 | 465 | **4.07** | 242 |

total compression ratio and speed for the four comparisons. The total compression ratio is the $total\_output\_size \div total\_input\_size \times 100$ and the total speed is $total\_input\_size \div 1000 \div total\_seconds\_spent$. The results show GeCo3 achieving the best compression ratio, both in relative and conditional compression. The latter shows improved compression capabilities, with average improvements of 11%, 29%, 35% and 57% over GeCo2, iDoComp, GDC2 and HRCM, respectively. This comes at a cost of being the slowest. The average increase in time over GeCo2, iDoComp, GDC2 and HRCM is 1.9×, 16.6×, 2.8× and 7.8×, respectively. Compared with GeCo2, the total improvement for PT, PA, GG, and HS is 12.4%, 11.7%, 10.8% and 10.1%. The total improvements are similar to the average improvement per chromosome. The computational RAM of GeCo3 is similar to GeCo2. The complete results per chromosome are shown in Supplementary Section 3 (Complete results for referential compression). These show that in the majority of pairs GeCo3 offers better compression, while the remaining by iDoComp.

## Discussion

In essence, this article considers the GeCo2 as a base, collecting its specific DNA models, and augments the mixture of models by using a neural network. The primary outcome is a new efficient tool, GeCo3. The results show a compression improvement at the cost of longer execution times and equivalent RAM.

For the evaluated datasets, this approach delivers the best results for the most significant and the highest repetitive sequences. One of the reasons for this is that for small sequences, the network spends a significant percentage of time adjusting. Moreover, we show the importance of selecting and deriving the appropriate network inputs as well as the influence of the number of hidden nodes. These can be used to increase compression at the cost of higher execution times.

Compared to other state-of-the-art compressors, this approach is typically slower but achieves better compression ratios both in reference-free and referential compression. Nevertheless, the compression times are progressively approximated according to the increase of the sequence size.

Specifically, the overall results show an improvement of the reference-free compression of GeCo3 in 18, 9%, and 5.8% over NAF and GeCo2, respectively. In reference-based compression, GeCo3 is able to provide compression gains of 11%, 29%, 35%, and 57% over GeCo2, iDoComp, GDC2, and HRCM, respectively.

The time trade-off and the symmetry of compression-decompression establish GeCo3 as a non-appropriate tool for on-the-fly decompression. Tools such as NAF [68] are efficient for this purpose, namely because the computational decompression speed is very high, which for industrial usage is mandatory. The purposes of tools such as GeCo3 are in another domain, namely long-term storage and data analysis.

The steady rise of analysis tools based on DNA sequence compression is showing its potential, with increasing applications and surprising results. Some of the applications are the estimation of the Kolmogorov complexity of genomes [92], rearrangements detection [93], sequence clustering [94], measurement of distances and phylogenetic trees computation [95], and metagenomics [6].

The main advantage of using efficient (lossless) compression-based data analysis is non-overestimation. Many analysis algorithms include multiple thresholds that use a consensus value for what is considered balanced and consistent, leaving space for overestimation. The problem is that using a consensus or average parameter for a specific analysis may overtake the limit of the estimation balance. Since data compression needs the appropriate decompressor to ensure the full recovery of the data, the compressor acts under a boundary that ensures that the limit is never overpassed (Kolmogorov complexity). This property is critical in data analysis because the data in use may be vital and sensitive, mainly when multiple models are used. Without a channel information limit and an efficient mixing model, the information that is embedded in the probabilities estimation of each model transits to the model choice.

The mixing method used to achieve these results assumes only that probabilities for the symbols are available. Because of this, it permits to be easily exported to other compressors or compressed-based data analysis tools that use multiple models. GeCo3 shows what compression improvements and execution times can be expected when using neural networks for the mixture of experts in DNA sequence compression.

This paper highlights the importance of expert mixing. Mixing has applications in all areas where there is the uncertainty of outcomes, and many expert opinions are available. This ranges from compression to climate modeling and, in the future, possibly the creation of legislation. While more traditional methods, such as weighted majority voting, are more efficient and can achieve accurate results, neural networks show promising results. With the development of specialized hardware instructions and data-types to be included in general-purpose CPUs, neural networks should become an even more attractive option for expert mixing.

One of the possible reasons this approach has higher compression than GeCo2 is due to the mixing output not being constrained by the inputs. By comparing the histograms in Fig. 3 for the sequence EnIn and OrSa (two of the sequences with higher gains), we can verify that GeCo3 appears to correct the models' probabilities greater than 0.8 to probabilities closer to 0.99. Therefore, in some way, it is betting more if at least four in five chances are accomplished. Referential histograms are presented in Supplementary Section 6 (Referential histograms); these are similar to the ones presented here.

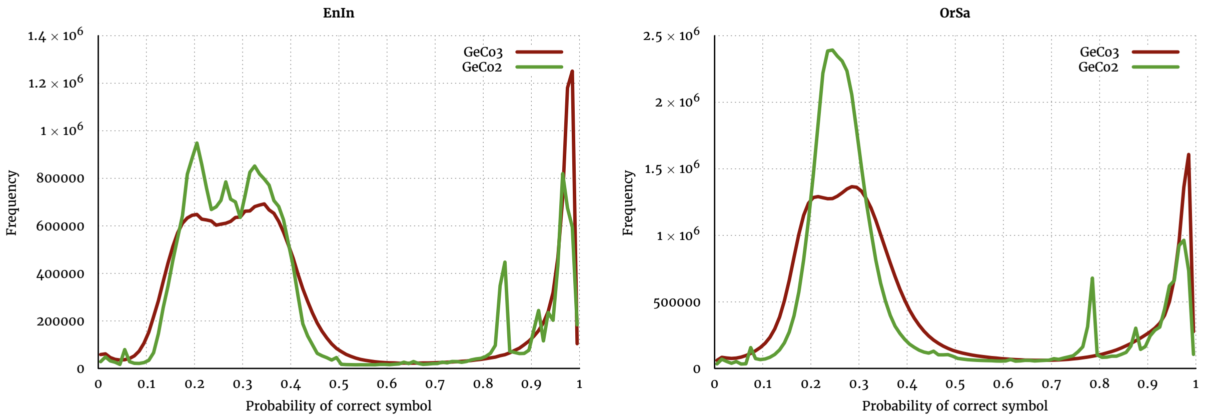


**Figure 3.** Comparison of histograms using the EnIn (*Entamoeba invadens*) and OrSa (*Oryza sativa*) genome sequences and the GeCo2 and GeCo3 as data compressors.

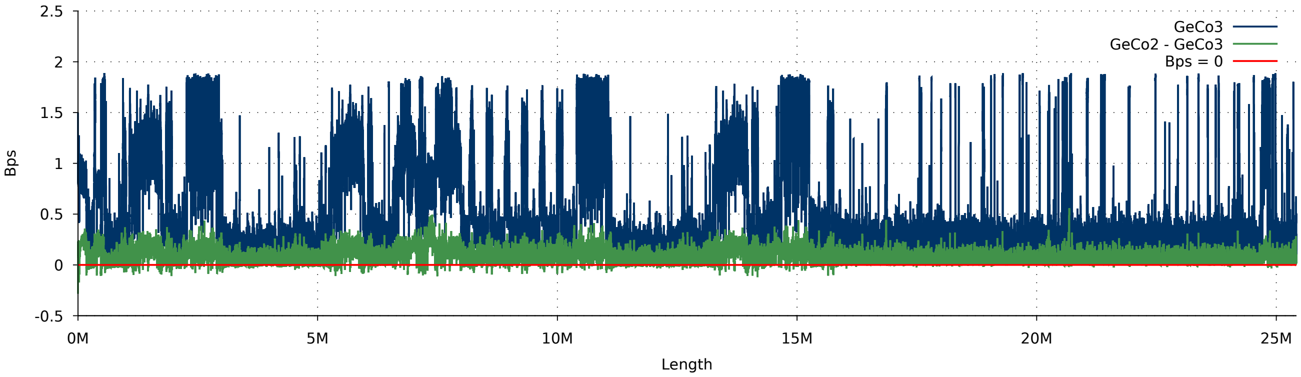


**Figure 4.** Complexity profile using the smoothed number of bits per symbol (Bps) of GeCo2 subtracted by GeCo3 Bps. The Bps were obtained by referential compression of PT_Y (Chromosome Y from *Pan troglodytes*) with the corresponding *Homo sapiens* chromosome, with the same parameters as in Table 4. Regions where the line rises above zero indicate that GeCo3 compresses more than GeCo2.

Another improvement is due to the higher percentage of symbols inferred correctly. For dataset four (DS4), GeCo3 has an average improvement of 1.5% in the number of symbols inferred correctly, where only the smallest sequence has a lower hit rate than GeCo2. Supplementary Section 2 (Percentage of symbols guessed correctly), presents the table of hit rate per sequence.

For referential compression, we show a complexity profile in Fig. 4. This profile reveals that GeCo3 outputs consistently a lower number of bits per symbol. The gains appear to be larger in places of higher sequence complexity, namely in the higher Bps regions. These regions are typically where rapid switching between smaller models should occur, suggesting that the neural network mixer can adapt faster than the approach used in GeCo2. Supplementary Section 4 (Referential complexity profiles), presents two additional complexity profiles with similar nature.

Finally, the training is maintained during the entire sequence. Because, we found out that doing early stopping leads to worse outcomes. This characteristic might be due to the advantages of over-fitting for non-stationary time series reported in [96].

Whichever the technology and application, the core method that we provide here, namely for combining the specific DNA models with neural networks, enables a substantial improvement in the precision of DNA sequence compression-based data analysis tools and in a substantial reduction of storage associated with DNA diversity.

## Availability of source code and requirements

- Project name: GeCo3
- Project home page: http://github.com/cobilab/geco3
- Operating system(s): Platform independent
- Programming language: C
- Other requirements: C compiler(e.g. gcc)
- License: GNU GPL

## Availability of supporting data and materials

Supplementary material includes all the information to install the benchmark compressors, download and compress the data.

### Competing Interests

The author(s) declare that they have no competing interests

### Funding

This work is partially funded by the national funds through the FCT in the context of the project UIDB/00127/2020. D.P. is funded by national funds through FCT - Fundação para a Ciência e a Tecnologia, I.P., under the Scientific Employment Stimulus - Institutional Call - CI-CTTI-94-ARH/2019.

## Author's Contributions

M.S., D.P., A.J.P. conceived and designed the experiments; M.S. implemented the algorithm; M.S. performed the experiments; M.S., D.P., A.J.P. analyzed the data; M.S., D.P., A.J.P. wrote the paper.

## References


1. Mahoney M, Data Compression Explained;. http://mattmahoney.net/dc/dce.html.
2. Stephens ZD, Lee SY, Faghri F, Campbell RH, Zhai C, Efron MJ, et al. Big data: astronomical or genomical? PLoS biology 2015;13(7):e1002195.
3. Fritz MHY, Leinonen R, Cochrane G, Birney E. Efficient storage of high throughput DNA sequencing data using reference-based compression. Genome research 2011;21(5):734–740.
4. Giancarlo R, Scaturro D, Utro F. Textual data compression in computational biology: a synopsis. Bioinformatics 2009;25(13):1575–1586.
5. Pratas D, Silva RM, Pinho AJ, Ferreira PJ. An alignment-free method to find and visualise rearrangements between pairs of DNA sequences. Scientific reports 2015;5(1):1–9.
6. Pratas D, Pinho AJ. Metagenomic composition analysis of sedimentary ancient DNA from the Isle of Wight. In: 2018 26th European Signal Processing Conference (EUSIPCO) IEEE; 2018. p. 1177–1181.
7. Pratas D, Pinho AJ, Ferreira PJ. Efficient compression of genomic sequences. In: 2016 Data Compression Conference (DCC) IEEE; 2016. p. 231–240.
8. Pratas D, Hosseini M, Pinho AJ. GeCo2: An Optimized Tool for Lossless Compression and Analysis of DNA Sequences. In: International Conference on Practical Applications of Computational Biology & Bioinformatics Springer; 2019. p. 137–145.
9. Pratas D, Hosseini M, Pinho AJ. Substitutional tolerant Markov models for relative compression of DNA sequences. In: International Conference on Practical Applications of Computational Biology & Bioinformatics Springer; 2017. p. 265–272.
10. Polikar R. Ensemble based systems in decision making. IEEE Circuits and systems magazine 2006;6(3):21–45.
11. Wolpert DH. Stacked generalization. Neural networks 1992;5(2):241–259.
12. Khalid S, Khalil T, Nasreen S. A survey of feature selection and feature extraction techniques in machine learning. In: 2014 Science and Information Conference IEEE; 2014. p. 372–378.
13. Covas C, Caetano T, Cruz A, Santos T, Dias L, Klein G, et al. Pedobacter lusitanus sp. nov., isolated from sludge of a deactivated uranium mine. International journal of systematic and evolutionary microbiology 2017;67(5):1339–1348.
14. Pyöriä L, Toppinen M, Mäntylä E, Hedman L, Aaltonen LM, Vihinen-Ranta M, et al. Extinct type of human parvovirus B19 persists in tonsillar B cells. Nature communications 2017;8(1):1–9.
15. Toppinen M, Perdomo M, Palo J, Simmonds P, Lycett S, Söderlund-Venermo M, et al. Bones hold the key to DNA virus history and epidemiology. Scientific reports 2015;5:17226.
16. Duggan AT, Perdomo MF, Piombino-Mascali D, Marciniak S, Poinar D, Emery MV, et al. 17th century variola virus reveals the recent history of smallpox. Current Biology 2016;26(24):3407–3412.
17. Teixeira H, Berg T, Uusitalo L, Fürhaupter K, Heiskanen AS, Mazik K, et al. A catalogue of marine biodiversity indicators. Frontiers in Marine Science 2016;3:207.
18. Cowan DA, Ramond JB, Makhalanyane TP, De Maayer P. Metagenomics of extreme environments. Current opinion in microbiology 2015;25:97–102.
19. Rieseberg LH. Chromosomal rearrangements and speciation. Trends in ecology & evolution 2001;16(7):351–358.
20. Roeder GS, Fink GR. DNA rearrangements associated with a transposable element in yeast. Cell 1980;21(1):239–249.
21. Sajantila A, Editors' Pick: Contamination has always been the issue! BioMed Central; 2014.
22. Harris K. Evidence for recent, population-specific evolution of the human mutation rate. Proceedings of the National Academy of Sciences 2015;112(11):3439–3444.
23. Jeong C, Di Rienzo A. Adaptations to local environments in modern human populations. Current opinion in genetics & development 2014;29:1–8.
24. Beres S, Kachroo P, Nasser W, Olsen R, Zhu L, Flores A, et al. Transcriptome remodeling contributes to epidemic disease caused by the human pathogen. Streptococcus pyogenes 2016;p. 00403–16.
25. Fumagalli M, Sironi M. Human genome variability, natural selection and infectious diseases. Current opinion in immunology 2014;30:9–16.
26. Long H, Sung W, Kucukyildirim S, Williams E, Miller SF, Guo W, et al. Evolutionary determinants of genome-wide nucleotide composition. Nature ecology & evolution 2018;p. 1.
27. Golan A. Foundations of Info-Metrics: Modeling and Inference with Imperfect Information. Oxford University Press; 2017.
28. Hernaez M, Pavlichin D, Weissman T, Ochoa I. Genomic Data Compression. Annual Review of Biomedical Data Science 2019;2:19–37.
29. Hosseini M, Pratas D, Pinho AJ. A survey on data compression methods for biological sequences. Information 2016;7(4):56.
30. Grumbach S, Tahi F. Compression of DNA sequences; 1993. p. 340–350.
31. Grumbach S, Tahi F. A new challenge for compression algorithms: genetic sequences 1994;30(6):875–886.
32. Rivals E, Delahaye JP, Dauchet M, Delgrange O. A guaranteed compression scheme for repetitive DNA sequences; 1996. p. 453.
33. Loewenstern D, Yianilos PN. Significantly lower entropy estimates for natural DNA sequences; 1997. p. 151–160.
34. Allison L, Edgoose T, Dix TI. Compression of strings with approximate repeats. In: Proc. of Intelligent Systems in Molecular Biology, ISMB-98 Montreal, Canada; 1998. p. 8–16.
35. Apostolico A, Lonardi S. Compression of biological sequences by greedy off-line textual substitution; 2000. p. 143–152.
36. Chen X, Li M, Ma B, Tromp J. DNACompress: fast and effective DNA sequence compression 2002;18(12):1696–1698.
37. Matsumoto T, Sadakane K, Imai H. Biological sequence compression algorithms. In: Dunker AK, Konagaya A, Miyano S, Takagi T, editors. Genome Informatics 2000: Proc. of the 11th Workshop Tokyo, Japan; 2000. p. 43–52.
38. Tabus I, Korodi G, Rissanen J. DNA sequence compression using the normalized maximum likelihood model for discrete regression; 2003. p. 253–262.
39. Korodi G, Tabus I. An efficient normalized maximum likelihood algorithm for DNA sequence compression 2005 Jan;23(1):3–34.
40. Cherniavsky N, Ladner R. Grammar-based compression of DNA sequences. University of Washington; 2004.
41. Manzini G, Rastero M. A simple and fast DNA compressor

2004;34:1397–1411.
42. A J T Lee CC, Chen C. DNAC: An Efficient Compression Algorithm for DNA Sequences. National Taiwan University, Taipei, Taiwan 10617, ROC 2004;1(1).
43. Behzadi B, Le Fessant F. DNA compression challenge revisited. In: Combinatorial Pattern Matching: Proc. of CPM-2005, vol. 3537 of LNCS Jeju Island, Korea: Springer-Verlag; 2005. p. 190–200.
44. Cao MD, Dix TI, Allison L, Mears C. A simple statistical algorithm for biological sequence compression; 2007. p. 43–52.
45. Vey G. Differential direct coding: a compression algorithm for nucleotide sequence data. Database 2009;2009.
46. Mishra KN, Aaggarwal A, Abdelhadi E, Srivastava D. An efficient horizontal and vertical method for online dna sequence compression. International Journal of Computer Applications 2010;3(1):39–46.
47. Rajeswari PR, Apparao A. GENBIT Compress-Algorithm for repetitive and non repetitive DNA sequences. International Journal of Computer Science and Information Technology 2010;2:25–29.
48. Gupta A, Agarwal S. A novel approach for compressing DNA sequences using semi-statistical compressor. International Journal of Computers and Applications 2011;33(3):245–251.
49. Zhu Z, Zhou J, Ji Z, Shi Y. DNA sequence compression using adaptive particle swarm optimization-based memetic algorithm 2011;15(5):643–658.
50. Pinho AJ, Pratas D, Ferreira PJSG. Bacteria DNA sequence compression using a mixture of finite-context models. In: Proc. of the IEEE Workshop on Statistical Signal Processing Nice, France; 2011. .
51. Pinho AJ, Ferreira PJSG, Neves AJR, Bastos CAC. On the representability of complete genomes by multiple competing finite-context (Markov) models 2011;6(6):e21588.
52. Roy S, Khatua S, Roy S, Bandyopadhyay SK. An efficient biological sequence compression technique using lut and repeat in the sequence. arXiv preprint arXiv:12095905 2012;.
53. Satyanvesh D, Balleda K, Padyana A, Baruah P. GenCodex - A Novel Algorithm for Compressing DNA sequences on Multi-cores and GPUs. In: Proc. IEEE, 19th International Conf. on High Performance Computing (HiPC), Pune, India; 2012. .
54. Bose T, Mohammed MH, Dutta A, Mande SS. BIND–An algorithm for loss-less compression of nucleotide sequence data. Journal of Biosciences 2012;37(4):785–789.
55. Li P, Wang S, Kim J, Xiong H, Ohno-Machado L, Jiang X. DNA-COMPACT: DNA Compression Based on a Pattern-Aware Contextual Modeling Technique 2013;8(11):e80377.
56. Pratas D, Pinho AJ. Exploring deep Markov models in genomic data compression using sequence pre-analysis; 2014. p. 2395–2399.
57. Sardaraz M, Tahir M, Ikram AA, Bajwa H. SeqCompress: An algorithm for biological sequence compression. Genomics 2014;104(4):225–228.
58. Guo H, Chen M, Liu X, Xie M. Genome compression based on Hilbert space filling curve. In: Proceedings of the 3rd International Conference on Management, Education, Information and Control (MEICI 2015), Shenyang, China; 2015. p. 29–31.
59. Xie X, Zhou S, Guan J. CoGI: Towards compressing genomes as an image. IEEE/ACM transactions on computational biology and bioinformatics 2015;12(6):1275–1285.
60. Chen M, Shao J, Jia X. Genome sequence compression based on optimized context weighting. Genetics and molecular research: GMR 2017;16(2).
61. Bakr NS, Sharawi AA. Improve the compression of bacterial DNA sequence. In: 2017 13th International Computer Engineering Conference (ICENCO) IEEE; 2017. p. 286–290.
62. Mansouri D, Yuan X. One-Bit DNA Compression Algorithm. In: International Conference on Neural Information Processing Springer; 2018. p. 378–386.
63. Wang R, Bai Y, Chu YS, Wang Z, Wang Y, Sun M, et al. DeepDNA: a hybrid convolutional and recurrent neural network for compressing human mitochondrial genomes. In: 2018 IEEE International Conference on Bioinformatics and Biomedicine (BIBM) IEEE; 2018. p. 270–274.
64. Wang R, Zang T, Wang Y. Human mitochondrial genome compression using machine learning techniques. Human genomics 2019;13(1):1–8.
65. Pratas D, Hosseini M, Silva JM, Pinho AJ. A Reference-Free Lossless Compression Algorithm for DNA Sequences Using a Competitive Prediction of Two Classes of Weighted Models. Entropy 2019;21(11):1074.
66. Mohammed MH, Dutta A, Bose T, Chadaram S, Mande SS. DELIMINATE—a fast and efficient method for loss-less compression of genomic sequences: sequence analysis. Bioinformatics 2012;28(19):2527–2529.
67. Pinho AJ, Pratas D. MFCompress: a compression tool for FASTA and multi-FASTA data. Bioinformatics 2014;30(1):117–118.
68. Kryukov K, Ueda MT, Nakagawa S, Imanishi T. Nucleotide Archival Format (NAF) enables efficient lossless reference-free compression of DNA sequences. Bioinformatics 2019;35(19):3826–3828.
69. Christley S, Lu Y, Li C, Xie X. Human genomes as email attachments. Bioinformatics 2009;25(2):274–275.
70. Brandon MC, Wallace DC, Baldi P. Data structures and compression algorithms for genomic sequence data. Bioinformatics 2009;25(14):1731–1738.
71. Ochoa I, Hernaez M, Weissman T. iDoComp: a compression scheme for assembled genomes. Bioinformatics 2015;31(5):626–633.
72. Deorowicz S, Danek A, Niemiec M. GDC 2: Compression of large collections of genomes. Scientific reports 2015;5:11565.
73. Kuruppu S, Puglisi SJ, Zobel J. Relative Lempel-Ziv compression of genomes for large-scale storage and retrieval. In: International Symposium on String Processing and Information Retrieval Springer; 2010. p. 201–206.
74. Wang C, Zhang D. A novel compression tool for efficient storage of genome resequencing data. Nucleic acids research 2011;39(7):e45–e45.
75. Kuruppu S, Puglisi SJ, Zobel J. Optimized relative Lempel-Ziv compression of genomes. In: Proceedings of the Thirty-Fourth Australasian Computer Science Conference-Volume 113; 2011. p. 91–98.
76. Deorowicz S, Grabowski S. Robust relative compression of genomes with random access. Bioinformatics 2011;27(21):2979–2986.
77. Pinho AJ, Pratas D, Garcia SP. GReEn: a tool for efficient compression of genome resequencing data. Nucleic acids research 2012;40(4):e27–e27.
78. Wandelt S, Leser U. FRESCO: Referential compression of highly similar sequences. IEEE/ACM Transactions on Computational Biology and Bioinformatics 2013;10(5):1275–1288.
79. Liu Y, Peng H, Wong L, Li J. High-speed and high-ratio referential genome compression. Bioinformatics 2017;33(21):3364–3372.
80. Fan W, Dai W, Li Y, Xiong H. Complementary Contextual Models with FM-Index for DNA Compression. In: 2017 Data Compression Conference (DCC) IEEE; 2017. p. 82–91.
81. Yao H, Ji Y, Li K, Liu S, He J, Wang R. HRCM: An Efficient Hybrid Referential Compression Method for Genomic Big Data. BioMed Research International 2019;2019.

82. Byron K, CMIX;. http://www.byronknoll.com/cmix.html.
83. Goyal M, Tatwawadi K, Chandak S, Ochoa I. DeepZip: Lossless Data Compression using Recurrent Neural Networks. arXiv preprint arXiv:181108162 2018;.
84. Absardi ZN, Javidan R. A Fast Reference-Free Genome Compression Using Deep Neural Networks. In: 2019 Big Data, Knowledge and Control Systems Engineering (BdKCSE) IEEE; 2019. p. 1–7.
85. Robbins H, Monro S. A stochastic approximation method. The annals of mathematical statistics 1951;p. 400–407.
86. Hiransha M, Gopalakrishnan EA, Menon VK, Soman K. NSE stock market prediction using deep-learning models. Procedia computer science 2018;132:1351–1362.
87. Glorot X, Bengio Y. Understanding the difficulty of training deep feedforward neural networks. In: Proceedings of the thirteenth international conference on artificial intelligence and statistics; 2010. p. 249–256.
88. LeCun YA, Bottou L, Orr GB, Müller KR. Efficient backprop. In: Neural networks: Tricks of the trade Springer; 2012.p. 9–48.
89. Pratas D, Pinho AJ. A DNA sequence corpus for compression benchmark. In: International Conference on Practical Applications of Computational Biology & Bioinformatics Springer; 2018. p. 208–215.
90. Kryukov K, Ueda MT, Nakagawa S, Imanishi T. Sequence Compression Benchmark (SCB) database—a comprehensive evaluation of reference-free compressors for FASTA-formatted sequences. bioRxiv 2019;p. 642553.
91. Ijdo J, Baldini A, Ward D, Reeders S, Wells R. Origin of human chromosome 2: an ancestral telomere-telomere fusion. Proceedings of the National Academy of Sciences 1991;88(20):9051–9055.
92. Pratas D, Pinho AJ. On the approximation of the Kolmogorov complexity for DNA sequences. In: Iberian Conference on Pattern Recognition and Image Analysis Springer; 2017. p. 259–266.
93. Hosseini M, Pratas D, Morgenstern B, Pinho AJ. Smash++: an alignment-free and memory-efficient tool to find genomic rearrangements. GigaScience 2020 05;9(5).
94. Cilibrasi R, Vitányi PM. Clustering by compression. IEEE Transactions on Information theory 2005;51(4):1523–1545.
95. Li M, Li X, Ma B, Vitányi P. Normalized information distance and whole mitochondrial genome phylogeny analysis. arXiv preprint cs/0111054 2008;.
96. Kim TY, Oh KJ, Kim C, Do JD. Artificial neural networks for non-stationary time series. Neurocomputing 2004;61:439–447.

Click here to access/download
**Supplementary Material**
Efficient_DNA_sequence_compression_with_Neural_Networks_Supp.pdf

Cover letter
Click here to download Personal Cover GeCo3_Cover.pdf

Aveiro, May 22, 2020

Dear Editor:

We are pleased to submit the manuscript entitled "**Efficient DNA sequence compression with Neural Networks**" for consideration for publication in GiGaScience as a Technical note.

The increasing production of genomic data has led to an intensifying need for models that can cope efficiently with DNA sequences lossless representation both for storage and compression-based data analysis.

Only a few recent articles propose the use of Neural Networks for DNA sequence compression. **However, they fall short** when compared with specific DNA compression tools, such as GeCo2. This limitation is given by the **absence of models specifically designed to DNA sequences**, for example, the high level of substitutions, rearrangements, contamination, and data heterogeneity.

In this work, **for the first time, we combine the power of Neural Networks with specific DNA models**, proposing a new efficient DNA sequence compressor (GeCo3) both for reference-free and reference-based compression.

We benchmark GeCo3 in eight extensive, fair and diverse datasets achieving a **substantial improvement over the state-of-the-art**, specifically of $2.4$%, $6$%, $4$%, and $5.9$% for reference-free, and $12.4$%, $11.7$%, $10.8$% and $10.1$% for reference-based compression. The cost is computational time ($2.4\times$ slower that GeCo2) that is minimized for sequences in the GigaByte or higher scales. The RAM is constant, and the tool scales efficiently, independently from the sequence size.

The tool and results are accompanied by a portable code package, requiring only the probabilities of the models as inputs, providing easy exportation to other data compressors or compression-based data analysis tools.

We confirm that this work is original and has not been published elsewhere, nor is it currently under consideration for publication elsewhere. We have no conflicts of interest to disclose.

Thank you for your consideration of this manuscript.

Sincerely,

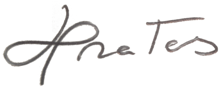

Diogo Pratas, PhD
Department of Electronics,
Telecommunications and Informatics,
University of Aveiro
pratas@ua.pt
+351914670195

Department of Virology
Faculty of Medicine,
University of Helsinki
diogo.pratas@helsinki.fi
