# Supplementary material for: Efficient DNA sequence compression with neural networks
Source: Gigascience. 2020 Nov 11;9(11):giaa119. doi: 10.1093/gigascience/giaa119 (PMC7657843; doi:10.1093/gigascience/giaa119)
Supplement: giaa119_GIGA-D-20-00154_Revision_1 [file giaa119_giga-d-20-00154_revision_1.pdf]

GigaScience

Efficient DNA sequence compression with neural networks

--Manuscript Draft--

**Manuscript Number:**	GIGA-D-20-00154R1
**Full Title:**	Efficient DNA sequence compression with neural networks
**Article Type:**	Technical Note
**Funding Information:**	Fundação para a Ciência e a Tecnologia (PT) (CI-CTTI-94-ARH/2019) — Mr Diogo Pratas
**Abstract:**	Background: The increasing production of genomic data has led to an intensified need for models that can cope efficiently with the lossless compression of DNA sequences. Important applications include long-term storage and compression-based data analysis.<br>In the literature, only a few recent articles propose the use of neural networks for DNA sequence compression. However, they fall short when compared with specific DNA compression tools, such as GeCo2. This limitation is due to the absence of models specifically designed for DNA sequences.<br>In this work, we combine the power of neural networks with specific DNA models. For this purpose, we created GeCo3, a new genomic sequence compressor that uses neural networks for mixing multiple context and substitution tolerant context models.<br><br>Findings: We benchmark GeCo3 as a reference-free DNA compressor in five datasets, including a balanced and comprehensive dataset of DNA sequences, the Y-chromosome and human mitogenome, two compilations of archaeal and virus genomes, four whole genomes, and two collections of FASTQ data of a human virome and ancient DNA. GeCo3 achieves a solid improvement in compression over the previous version (GeCo2) of 2.4%, 7.1%, 6.1%, 5.8% and 6%, respectively.<br>As a reference-based DNA compressor, we benchmark GeCo3 in four datasets constituted by the pairwise compression of the chromosomes of the genomes of several primates. GeCo3 improves the compression in 12.4%, 11.7%, 10.8% and 10.1% over the state-of-the-art. The cost of this compression improvement is some additional computational time (1.7× to 3× slower that GeCo2). The RAM is constant, and the tool scales efficiently, independently from the sequence size. Overall, these values outperform the state-of-the-art.<br><br>Conclusions: GeCo3 is a genomic sequence compressor with a neural network mixing approach, that provides additional gains over top specific genomic compressors. The proposed mixing method is portable, requiring only the probabilities of the models as inputs, providing easy adaptation to other data compressors or compression-based data analysis tools. GeCo3 is released under GPLv3 and is available for free download at https://github.com/cobilab/geco3 .
**Corresponding Author:**	Milton Silva<br>Universidade de Aveiro Departamento de Electronica Telecomunicacoes e Informatica<br>Aveiro, PORTUGAL
**Corresponding Author Secondary Information:**	
**Corresponding Author's Institution:**	Universidade de Aveiro Departamento de Electronica Telecomunicacoes e Informatica
**Corresponding Author's Secondary Institution:**	
**First Author:**	Milton Silva
**First Author Secondary Information:**	
**Order of Authors:**	Milton Silva
	Diogo Pratas
	Armando Pinho

Powered by Editorial Manager® and ProduXion Manager® from Aries Systems Corporation

Order of Authors Secondary Information:	
Response to Reviewers:	Thank you for the opportunity to revise the manuscript.<br>The answers to reviewers are included in the attached file GeCo3_Answers.pdf.<br>As suggested by the editor we have also included the RRID and biotoolsID identifiers in the manuscript.
Additional Information:	
Question	Response
Are you submitting this manuscript to a special series or article collection?	No
**Experimental design and statistics**<br><br>Full details of the experimental design and statistical methods used should be given in the Methods section, as detailed in our Minimum Standards Reporting Checklist. Information essential to interpreting the data presented should be made available in the figure legends.<br><br>Have you included all the information requested in your manuscript?	Yes
**Resources**<br><br>A description of all resources used, including antibodies, cell lines, animals and software tools, with enough information to allow them to be uniquely identified, should be included in the Methods section. Authors are strongly encouraged to cite Research Resource Identifiers (RRIDs) for antibodies, model organisms and tools, where possible.<br><br>Have you included the information requested as detailed in our Minimum Standards Reporting Checklist?	Yes
**Availability of data and materials**<br><br>All datasets and code on which the conclusions of the paper rely must be either included in your submission or deposited in publicly available repositories (where available and ethically	Yes

appropriate), referencing such data using a unique identifier in the references and in the “Availability of Data and Materials” section of your manuscript.<br><br>Have you have met the above requirement as detailed in our Minimum Standards Reporting Checklist?	

Manuscript

Click here to access/download;Manuscript;Efficient_DNA_sequence_compr

OXFORD

(GIGA)^n SCIENCE

GigaScience, 2020, 1–13

doi: xx.xxxx/xxxx
Manuscript in Preparation
Technical Note

TECHNICAL NOTE

Efficient DNA sequence compression with neural networks

Milton Silva[1,2,*], Diogo Pratas[1,2,3,*] and Armando J. Pinho[1,2]

[1]Institute of Electronics and Informatics Engineering of Aveiro, University of Aveiro, Portugal and [2]Department of Electronics Telecommunications and Informatics, University of Aveiro, Portugal and [3]Department of Virology, University of Helsinki, Finland

[*]teixeirasilva@ua.pt; pratas@ua.pt;

Abstract

Background: The increasing production of genomic data has led to an intensified need for models that can cope efficiently with the lossless compression of DNA sequences. Important applications include long-term storage and compression-based data analysis. In the literature, only a few recent articles propose the use of neural networks for DNA sequence compression. However, they fall short when compared with specific DNA compression tools, such as GeCo2. This limitation is due to the absence of models specifically designed for DNA sequences. In this work, we combine the power of neural networks with specific DNA models. For this purpose, we created GeCo3, a new genomic sequence compressor that uses neural networks for mixing multiple context and substitution tolerant context models. **Findings:** We benchmark GeCo3 as a reference-free DNA compressor in five datasets, including a balanced and comprehensive dataset of DNA sequences, the Y-chromosome and human mitogenome, two compilations of archaeal and virus genomes, four whole genomes, and two collections of FASTQ data of a human virome and ancient DNA. GeCo3 achieves a solid improvement in compression over the previous version (GeCo2) of 2.4%, 7.1%, 6.1%, 5.8%, and 6%, respectively. As a reference-based DNA compressor, we benchmark GeCo3 in four datasets constituted by the pairwise compression of the chromosomes of the genomes of several primates. GeCo3 improves the compression in 12.4%, 11.7%, 10.8% and 10.1% over the state-of-the-art. The cost of this compression improvement is some additional computational time (1.7× to 3× slower than GeCo2). The RAM is constant, and the tool scales efficiently, independently from the sequence size. Overall, these values outperform the state-of-the-art. **Conclusions:** GeCo3 is a genomic sequence compressor with a neural network mixing approach, that provides additional gains over top specific genomic compressors. The proposed mixing method is portable, requiring only the probabilities of the models as inputs, providing easy adaptation to other data compressors or compression-based data analysis tools. GeCo3 is released under GPLv3 and is available for free download at https://github.com/cobilab/geco3.

Key words: Lossless data compression, DNA sequence compression, Context mixing, Neural networks, Mixture of experts

Introduction

The DNA sequencing rate is increasing exponentially, stretching the genomics storage requirements to unprecedented dimensions. Several projections show that by the year 2025, between 2 to 40 exabytes of additional storage will be needed per year [1]. Discarding a significant fraction of the data is not a feasible alternative, given its high importance in many contexts, for example, in biomedical (e.g., personalized medicine) and anthropological fields.

The representation of genomic data usually consists of DNA sequences accompanied by additional channels, such as headers, quality-scores, variant positions, among others, that vary from type and purpose. Different file formats store the sequence with subsets of this metadata, but the core remains the DNA sequences. The compression of these sequences has

Compiled on: August 19, 2020.
Draft manuscript prepared by the author.

been widely approached with general- and specific-purpose compressors, where the latter is now significantly started to be used given its substantial compression gains.

Specialized DNA compressors achieve substantially higher compression than general-purpose because most of these compressors use various models that take into account specific properties of DNA, such as inverted repeats and high-level of substitutions [2, 3]. However, the efficient combination of multiple models for DNA sequence compression is not a trivial problem. The complexity associated with the development of improved algorithms to combine those predictions [4] and the specificities of the genomic data, namely, heterogeneity and non-stationarity, delivers a highly demanding task.

In this paper, we address the problem of combining the predictions of different models to produce an improved predictive model and, by consequence, improve the compression of DNA sequences. Accordingly, we take the specific DNA models from GeCo2 [3], namely the context and substitution tolerant context models [5], and implement a mixture of these models with a neural network.

Therefore, instead of combining only the models' predictions with the algebraic combiner of GeCo2, where weights are attributed to each model and updated based on the model performance with a particular forgetting factor, we improve the mixture of experts using ensemble methods [6].

Specifically, we use a stacked generalization approach [7], namely applying a neural network meta-model that takes as inputs the outputs of other models and is trained to learn the mapping between the models' outputs and the actual correct outputs. To implement the stack generalization, we use a multilayer perceptron. This network takes as inputs the probabilities of each model as well as derived features [8] that represent past model performance, while it outputs the probabilities for each symbol, which are redirected to an arithmetic encoder.

For evaluation, we created a new DNA compression tool (GeCo3) and benchmark it both reference-free and referential compression. Nine datasets are employed for reference-free and reference-based compression benchmarks, containing different sequence nature, lengths, and redundancy levels.

The results show a consistent improvement in the compression ratio of GeCo3 over state-of-the-art DNA compressors, both in reference-free and reference-based approaches, enabling the use of GeCo3 as a long-term storage tool.

Although data compression is the natural approach for decreasing the storage of DNA sequences losslessly [9], it can also be efficiently applied to sequence analysis and prediction using special-purpose compressors [10, 11, 12]. Therefore, this improvement also enables increasing the precision of DNA sequence compression-based analysis tools. In order to facilitate the exportation of the mixing method to other data compression or data analysis tools, we provide the reusable and modular mixer code and instructions on how to integrate it easily.

In the following subsection, we provide background on reference-free and reference-based DNA sequence compression. Then, we describe GeCo3 in detail and, finally, we provide the benchmark results and some discussion.

DNA sequence compression

Genomes are found in the most diverse places, for example, in extreme environments as uranium mines [13], in soft and hard tissues [14, 15], ancient cadavers [16], marine environments [17], or deep subterranean habitats [18]. The environment and species interactions are a key for genome adaptation, providing a wide diversity in characteristics, namely high copy number, high heterogeneity, high level of substitution mutations, or multiple rearrangements, such as fissions, fusions, translocations, or inverted repeats [19, 20]. Additionally, since genomic (DNA) sequences are an output of biochemical and computational methods, these sequences may have other alteration sources, for example contamination [21], environmental factors [22, 23], pathogenic species included in the samples [24, 25], and unknown sources [26]. Therefore, representing genomic sequences requires the ability to model heterogeneous, dynamic, incomplete, and imperfect information [27].

The above specific characteristics led to the development of the field which studies and constructs specific genomic data compressors [28, 29]. This field has now 27 years and started with Biocompress [30]. Afterwards, several algorithms emerged, mostly modeling the existence of exact or approximate repeated and inverted repeated regions, through the usage of simple bit encoding, context modeling, or dictionary approaches [31, 32, 33, 34, 35, 36, 37, 38, 39, 40, 41, 42, 43, 44, 45, 46, 47, 48, 49, 50, 51, 52, 53, 54, 53, 54, 55, 56, 57, 58, 59, 2, 60, 61, 62, 3, 63, 64, 65].

The FASTA format development permitted to standardize the co-existence of DNA sequences (in a visible horizontal range) along with annotations (headers). Usually, the DNA sequence is substantially the most abundant part of this data and, hence, multiple tools use specialized DNA compression algorithms combined with simple header coding, namely Delimինate [66], MFCompress [67], and NAF [68]. Notwithstanding, for comparison purposes with DNA sequence compressors, setting a minimal header, asymptotically, increases its irrelevance relative to the DNA sequence according to its size.

From all the previous algorithms, the most efficient according to compression ratio in the wide diversity of DNA sequences are XM [44], GeCo2 [3], and Jarvis [65]. These compressors apply statistical and algorithmic model mixtures combined with arithmetic encoding. Specifically, the XM algorithm [44] combines three types of experts, namely repeat models, a low-order context model, and a short memory context model of 512 bytes. The GeCo2 algorithm [3] uses soft-blending cooperation between context models and substitution tolerant context models [5] with a specific forgetting factor for each model. The Jarvis compressor [65] uses a competitive prediction model to estimate, for each symbol, the best class of models to be used; there are two classes of models: weighted context models and weighted stochastic repeat models, where both classes of models use specific sub-programs to handle inverted repeats efficiently.

Some compressors use a reference genome as an additional input. This approach is called referential compression, and it started to gain momentum in 2009 [69, 70]. Referential compressors attained substantially higher compression ratios compared to reference-free compressors. The resulting compressed lengths can be hundreds or thousands of times smaller than the original file [71, 72]. As an example, an entire human genome of about 3GB can be compressed to 4MB by referential compression; on the other hand, a reference-free compressor minimizes the data to 580MB, approximately. The majority of reference-based compression algorithms use dictionaries, repeats models, or context models [69, 70, 73, 74, 75, 76, 77, 55, 78, 71, 72, 79, 80, 3, 81]. From the previous compressors, the most productive, according to compression ratio, are HiRGC [79], GeCo2 [3], iDoComp [71], GDC2 [72] and HRCM [81]. The HiRGC [79] is based on a 2-bit encoding scheme and an advanced greedy-matching search on a hash table. The GeCo2 [3] is described above. The iDoComp [71] uses a suffix array for loading the reference and later applies a greedy parsing of the target that benefits the substitutional single nucleotide polymorphisms that occur in higher number. The GDC2 [72] performs a Ziv-Lempel factoring combined with a second-level factoring and followed by arithmetic coding. The HRCM [81] explores sequence information extraction, followed by sequence

information matching and further encoding.

The usage of neural networks to compress DNA sequences is seen in DeepDNA [63]. The DeepDNA is a special purpose DNA compressor without specialized models. It uses an hybrid approach with a convolutional layer to capture the genome's local features and a recurrent layer to model long-term dependencies.

In general-purpose sequence compressors, the idea of using neural networks to mix probabilities is seen in [4]. In this case, it is called logistic mixing. Logistic mixing can be viewed as using a neural network without hidden layers and a simpler update rule than backpropagation. Other general-purpose compressors followed the same line, namely Cmix [82] and DeepZip [83]. The Cmix [82] uses recurrent neural networks trained with stochastic gradient descent for context mixing. The DeepZip [83] also uses recurrent neural networks, both as predictors (models) and as mixers.

Although the best general-purpose compressors use complex computational models, namely based on neural networks, it has been shown that they still have lower compression capabilities (5-10%) using substantially higher computational time according to the most efficient specific compressors [83]. The discrepancy of precision is higher when the method is designed for fast computations [84]. The main reason that the best general-purpose algorithms (using neural networks) are not so efficient is that they do not use specific DNA models that take into account the algorithmic nature of genomic sequences, harming the model sensitivity.

In this paper, we combine the sensitivity of specific DNA models, namely the usage of multiple context models combined with DNA specific algorithmic models, with the power of neural networks for context mixing.

Methods

In this section, we present the methods that describe the proposed compressor (GeCo3). GeCo3 uses a combination of multiple context models and substitution tolerant context models of several order-depths. The neural network provides an efficient combination of these models. Therefore, we describe the new method with the main focus on the neural network, including the inputs, updates, outputs, and training process.

Neural network structure

The model mixing is constructed using a feed-forward artificial neural network trained with stochastic gradient descent [85]. This choice is motivated by implementation simplicity and competitive performance compared to more complex neural networks [86]. The activation function for this network is the sigmoid, and the loss function is the mean squared error. The network structure is fully connected with one hidden layer, as seen in Fig. 1b. One bias neuron is used for the input and hidden layer, while the weights respect the Xavier initialization according to [87]. Although we empirically tested different activation functions (ReLu, TanH) and a higher number of hidden layers, the most efficient structure was obtained with the previous description.

We introduced two parameters for the GeCo3 compression tool in order to control the number of nodes of the hidden layer and the learning rate. These parameters are written in the compressed file header to ensure a lossless decompression.

Neural network inputs

The stretched probabilities of each symbol are used as inputs to the network. These are given by

$$p_{i,j} = stretch\left(\frac{1+f_{i,j}}{\sum_{m\in\Theta} 1+f_{i,m}}\right) - stretch\left(mean_p\right), \qquad (1)$$

where $f_{i,j}$ is the frequency of symbol j for model i with Θ as the set of all symbol and $mean_p$ is the mean probability of each symbol.

We stretch the probabilities according to the work of Mahoney [4]. The effects of stretching can be seen in Supplementary Section 1 (Stretching function plot). The inputs are normalized for forcing the average to be close to zero by subtracting the stretched mean probability, which, for the case of DNA, we assume to be 0.25. The normalization and its motivation are explained in [88]. Stretching the probabilities has the effect of scaling them in a non-linear way, which increases the weights of probabilities near zero and one.

The context models, substitution tolerant context models, and the mixed probabilities of GeCo2 are used as input models. This inclusion means that the mixing done in GeCo2 is not discarded, but are used as an additional input to the neural network.

We extract features from the context (the last n symbols) and also calculate model and network performance indicators to improve the network predictions. These are used as inputs to the neural network. Three performance indicators are derived for each mode according to the names *hit*, *best*, and *bits*. These features correspond to three input nodes per model, as seen in Fig. 1b.

To measure how precise model i is voting, we use

$$hit_{i,n} = \begin{cases} hit_{i,n-1}, & \text{if } \forall x,y \in \Theta : p_{i,x} = p_{i,y} \\ hit_{i,n-1} + 0.1, & \text{if } \forall x \in \Theta : p_{i,sym} > p_{i,x} \\ hit_{i,n-1} - 0.1, & \text{otherwise.} \end{cases} \qquad (2)$$

The symbol with the highest probability is considered the vote of the model. Each time the model votes correctly, *hit* is increased. If the model abstains (probabilities of each symbol are equal), then *hit* remains the same; otherwise, it decreases.

For each model, we also measure if it has assigned the highest probability to the correct symbol, compared to all other models. This is given by

$$best_{i,n} = \begin{cases} best_{i,n-1}, & \text{if } \forall x,y \in \Theta : p_{i,x} = p_{i,y} \\ best_{i,n-1} + 0.1, & \text{if } p_{i,sym} \geq p_{k,sym} \\ best_{i,n-1} - 0.1, & \text{otherwise.} \end{cases} \qquad (3)$$

The update rules for *best* are similar to *hit* and both have a domain of $[-1, 1]$.

As an approximation to the average number of bits the model would output, we use an exponential moving average

$$bits_{i,n} = \alpha_1 \cdot (-\log_2(p_{i,sym}) + \log_2(mean_p)) + (1-\alpha_1) \cdot bits_{i,n-1}, \qquad (4)$$

with $\alpha_1 = 0.15$. This input is also normalized such that the average value is close to zero.

In Eqs. (2), (3) and (4), $p_{i,sym}$ is the probability assigned by model i to the actual symbol in the sequence. To reach these features and their constants, we tested each with a couple of files from one dataset and adjusted until finding a value that produced satisfactory results.

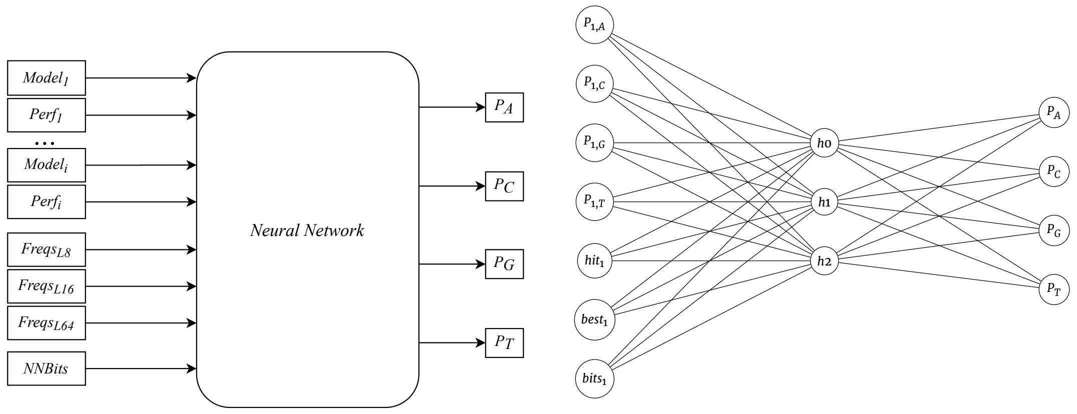

(a) High level architecture **(b)** Neural network detail

Figure 1. Mixer architecture: (a) High level overview of inputs to the neural network (mixer) used in GeCo3. *Model*$_1$ through *Model*$_i$ represent the GeCo2 model outputs (probabilities for A, C, T, G). *Perf* represents the performance metrics (*hit*, *best*, *bits*) for each model. *Freqs* are the frequencies for the last 8, 16, and 64 symbols. *NNBits* is a moving average of the approximate number of bits that the neural network is producing. The network outputs represent the non-normalized probabilities for each DNA symbol. (b) A fully connected neural network with one hidden layer. For illustration purposes, this neural network only has the inputs corresponding to one model and the three features that evaluate the model performance. The frequencies of the last 8, 16, and 64 symbols, as well as the *NNBits* and the bias neurons, are omitted.

The features extracted from the context are the probabilities of each symbol for the last 8, 16, and 64 symbols. These represent a total of twelve input nodes. In Fig. 1a, these nodes are represented by $Freqs_{L8}$, $Freqs_{L16}$ and $Freqs_{L64}$. For example, to obtain the probabilities for the last eight symbols with the sequence ACAGTAAA, the number of A's is divided by the number of total symbols, so the frequency of symbol A is 5/8 and for the other symbols is 1/8. These probabilities are then scaled to fit between -1 and 1.

In Fig. 1a, *NNBits* represents the exponential moving average of the approximate number of bits and is given by

$$nnbits_n = \alpha_2 \cdot (-\log_2(p_{sym}) + \log_2(mean_p)) + (1 - \alpha_2) \cdot nnbits_{n-1}, \quad (5)$$

with p_{sym} as the probability the network assigned to the correct symbol and $\alpha_2 = 0.5$.

Updating model performance features

As an example of how to update the features, consider two symbols and three models, and assume all features start equal to zero. Model 1 assigns the probabilities $[0.5, 0.5]$, meaning that the model abstains and, as such, no change is made to *hit* or *best*. Also, $bits_1$ would be equal to zero. The probabilities for model 2 and 3 are $[0.7, 0.3]$ and $[0.8, 0.2]$, respectively. Assuming the models voted correctly, then *hit* is now $0 + 0.1 = 0.1$ for both. Because model 3 assigned the highest probability to the correct symbol then $best_3$ is now $0+0.1 = 0.1$, and $best_2$ becomes -0.1. Moreover, $bits_2$ would become $bits_2 = 0.15 \cdot (-\log_2(0.7) + log_2(0.5))$ and $bits_3 = 0.15 \cdot (-\log_2(0.8) + log_2(0.5))$.

Neural network outputs and training

One node per symbol is used as output from the network. After the result is transferred to the encoder, the network is trained with the current symbol using the learning rate specified within the program input.

When compared to GeCo2, the results of the new mixing contain two main differences. First, the sum of output nodes is different from one. This outcome is corrected by dividing the node's output by the sum of all nodes. The second difference is that the new approach outputs probabilities in the range $]0, 1[$, while in GeCo2, the mixing always yielded probabilities inside the range of the models.

Results

In this section, we benchmark GeCo3 against state-of-the-art tools in both reference-free and referential compression approaches. In the following subsection, we describe the datasets and materials used for the benchmark, followed by the comparison with GeCo2 using different characteristics, number of models, and data redundancy. Finally, we provide the full benchmark for the nine datasets.

Datasets and materials

The benchmark includes nine datasets. Five datasets are selected for reference-free compression, including

- **DS1**: two compilations of FASTQ data, namely a human virome (Virome) [89] and ancient DNA from a Denisova individual (Denisova) [90];
- **DS2**: four whole genomes: human (HoSaC), chimpanzee (PaTrC), gorilla (GoGoC), and the Norway spruce (PiAbC);
- **DS3**: two compilations of archaeal (Archaea) and viral genomes (Virus);
- **DS4**: highly repetitive DNA with the human Y-chromosome (HoSaY) and a human mitogenome collection (Mito) (proposed in [91]);
- **DS5**: a comprehensive-balanced dataset (proposed in [92]), containing the following sequences:
  - HoSa: chromosome 4 of the reference human genome
  - GaGa: chromosome 2 of *G. gallus*;
  - DaRe: chromosome 3 of *D. rerio*;
  - OrSa: chromosome 1 of *O. sativa Japonica*;
  - DrMe: chromosome 2 of *D. miranda*;
  - EnIn: genome of *E. invadens*;
  - ScPo: genome of *S. pomb*;
  - PlFa: genome of *P. falciparum*;
  - EsCo: genome of *E. coli*;
  - HaHi: genome of *H. hispanica*;
  - AeCa: genome of *A. camini*;

- HePy: genome of *H. pylori*;
- YeMi: genome of *Yellowstone lake* mimivirus;
- AgPh: genome of *Aggregatibacter* phage S1249;
- BuEb: genome of *Bundibugyo ebolavirus*.

On the other hand, to benchmark the reference-based approach, we use the complete genomes of four primates (human, gorilla, chimpanzee, and orangutan) with a pairwise chromosomal compression. Non-human chromosomes are concatenated to match the human chromosomal fusion [93]. For each chromosomal pair, the following compression was performed

- **DSR1**: chimpanzee (PT) using human (HS) as a reference;
- **DSR2**: orangutan (PA) using human (HS) as a reference;
- **DSR3**: gorilla (GG) using human (HS) as a reference;
- **DSR4**: human (HS) using gorilla (GG) as a reference.

All the materials to replicate the results, including the sequence identifiers, URL, filtering applications, and associated commands, can be found at the Supplementary Section 8 (Reproducibility).

Neural network mixing compression

In order to assess the performance of the neural network mixing, we compare GeCo2 with GeCo3. To ensure a fair comparison, the compression modes, including the models and parameters, are kept identical for both programs.

In Table 1, GeCo2 and GeCo3 are compared using the compression modes published in [3]. The overall compression improves by 1.93%, and the average improvement is 1.06%. The larger sequences (larger than ScPo) have average improvements of 2.04%, while the remaining have modest improvements of 0.4%. Only the two smallest sequences show negative improvement, given the absence of enough time to train the network. Additionally, the eight bytes that are used to transmit the two network parameters to the decompressor are a significant percentage of the total size, unlike in larger sequences. Overall, GeCo3 improves the compression of the whole dataset by more than 1.9%.

Neural network mixing computational resources

Regarding computational resources, the mixing modification is 2.7× slower, as shown in Table 1. The computation was performed on an Intel(R) Core(TM) i7-6700 CPU @ 3.40GHz running Linux 5.4.0 with the scaling governor set to performance and 32GB of RAM. The new mixing approach is always slower because GeCo2's mixing is still used, not as a result of the encoder, but rather as an input to the network. The difference in RAM usage of both approaches is less than 1 MB, which corresponds to the size of the neural network and the derived features for each model.

The number of hidden nodes is chosen to fit in the vector registers in order to take full advantage of the vectorized instructions. Accordingly, we set the number of hidden nodes as a multiple of eight, where floating points of four bytes represent the nodes and 32 bytes represent the vector registers.

Effects of the hidden layer size on mixing

Increasing or decreasing the number of hidden nodes affects the number of weights, and it also affects compression, as can be seen in Fig. 2. Increasing the number of nodes increases the compression up to a point. This point varies from sequence to sequence; however, the abruptest gains in compression generally occur until 24 hidden nodes. As expected, increasing the number of hidden nodes leads to an increase in execution time and a progressive decline of compression gain. These results are also consistent in referential compression as seen in Supplementary Section 6 (Referential hidden nodes effect).

The importance of derived features on mixing

We removed the derived features from the inputs to the network to assess its impact on the mixing performance. The results are present in Table 2.

When using just the models' probabilities as inputs, the compression is more efficient than GeCo2 by a small margin (0.18%), while, in the majority of the sequences, there is no improvement. By adding the result of the GeCo2 mixing as an input, the improvement increases to 1.36%. The gain esca-

Table 1. Number of bytes needed to represent each DNA sequence for GeCo2 and GeCo3 compressors. The column mode applies to both compression methods, while the learning rate and the number of hidden nodes only apply to the latter.

ID	GeCo2 bytes	GeCo3 bytes	GeCo2 secs	GeCo3 secs	Mode	L.Rate	H.Nodes
HoSa	38,845,642	37,891,143	223	598	12	0.03	64
GaGa	33,877,671	33,411,628	160	424	11	0.03	64
DaRe	11,488,819	11,189,716	64	189	10	0.03	64
OrSa	8,646,543	8,434,878	44	133	10	0.03	64
DrMe	7,481,093	7,379,992	33	99	10	0.03	64
EnIn	5,170,889	5,066,670	26	75	9	0.05	64
ScPo	2,518,963	**2,511,054**	11	24	8	0.03	40
PlFa	1,925,726	**1,906,919**	10	22	7	0.03	40
EsCo	1,098,552	**1,094,298**	2	8	6	0.03	40
HaHi	902,831	**896,037**	2	6	5	0.04	40
AeCa	380,115	**377,343**	1	2	5	0.04	16
HePy	375,481	**373,583**	1	3	4	0.04	40
YeMi	16,798	**16,793**	0	0	3	0.09	24
AgPh	**10,708**	10,715	0	0	2	0.06	16
BuEb	**4,686**	**4,686**	0	0	1	0.06	8
Total	112,744,517	110,565,455	577	1,583			

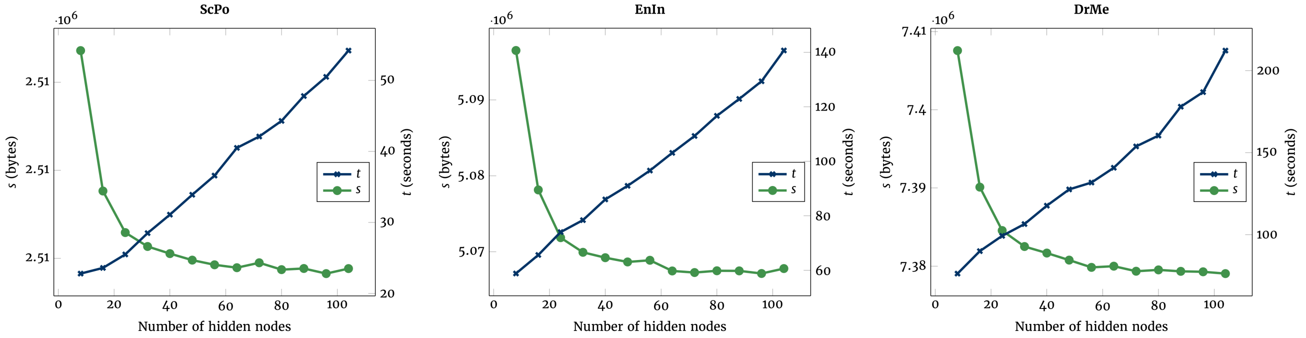

Figure 2. Number of bytes (*s*) and time (*t*) according to the number of hidden nodes for the reference-free compression of ScPo, EnIn, and DrMe sequence genomes.

lates, having an improvement of 1.73%, when using the context models and tolerant context models as inputs and the derived features.

Table 2. Number of bytes needed to represent each DNA sequence using the GeCo3 compressor with specific conditions. For the column named Models, only the context models and tolerant context models of GeCo2 were used as network inputs. For "Models + GeCo2", the result of GeCo2 mixing was also used as input. With "Models + Derived" the inputs for the network were the same as "Models" with the derived features added. The compression modes are the same as in Table 1.

ID	Models	Models + GeCo2	Models + Derived
HoSa	38,556,039	38,153,358	**37,943,933**
GaGa	33,758,606	33,548,929	**33,444,816**
DaRe	11,615,937	11,280,688	**11,251,390**
OrSa	8,694,790	8,517,947	**8,471,715**
DrMe	7,475,341	7,414,919	**7,392,290**
EnIn	5,183,237	5,095,391	**5,087,359**
ScPo	2,524,818	2,514,188	**2,513,085**
PlFa	1,928,282	1,912,745	**1,912,176**
EsCo	1,104,646	1,095,589	**1,096,255**
HaHi	903,019	898,280	**898,145**
AeCa	378,226	377,857	**377,696**
HePy	379,285	**374,364**	374,975
YeMi	16,901	**16,827**	16,882
AgPh	10,744	**10,727**	10,731
BuEb	**4,694**	4,696	4,698
Total	112,534,565	111,216,505	110,796,146

Scaling the number of models

GeCo2 and GeCo3 contain several modes (compression levels), which are parameterized combinations of models with diverse neural network characteristics. To see how the compression of the new approach scales with more models, we introduced mode 16 with a total of 21 models. This new mode was used to compress the sequences of HoSa to HePy (by size order). For the remaining sequences, the same models were used as in Table 1. We used this approach because increasing the number of models was incapable of improving the compression of GeCo3 and GeCo2, given the smaller dimensions of these sequences. The number of hidden nodes was also adjusted until no tangible improvements in compression were observed.

The results in Table 3 show that the distance between the approaches increases from 1.93% to 2.43%. The time difference reduces from 2.7× to 2.0×. This reduction is due to the increased percentage of time spent by the higher-order context models. These results show that neural network mixing can scale with the number of models. The forgetting factors for this new mode were not tuned, due to the use of a large number of models. Therefore, with this tuning, additional gains can be observed. Nevertheless, this shows another advantage of this new mixing, which is that there are only two parameters that need tuning regardless of the number of models. As the sequence size and the number of models increases, there is almost no tuning required, with the optimal values being around 0.03 for the learning rate and 64 hidden nodes.

Compressing highly repetitive and large sequences

In this subsection, we show how the reference-free compression scales with the new mixing using highly repetitive and extensive sequences, namely in the gigabyte scale. Four datasets are selected, and the results shown in Table 3.

According to the results from Table 3, GeCo3 compresses the highly repetitive sequences (DS3 and DS4) with an average of 6.6% compared to GeCo2 using more 1.9× time. For the larger sequences of DS1 and DS2, GeCo3 has an average compression improvement of 3.2% in the primates, 8.2% in the spruce (PiAbC), 11.8% for the Virome and 5.2% for Denisova, with a 2.6× average slower execution time. These results show that the compression of longer repetitive sequences present higher compression gains.

Reference-free sequence compression bechmark

In this subsection, we compare GeCo3 with other specialized reference-free compressors, namely XM (v3.0) [44], GeCo2 (previously compared), Jarvis [65], and NAF [68]. As presented in Table 3, GeCo3 achieves the best total size in three out of five datasets. In DS3 and DS4, GeCo3 was unable to achieve the best compression, delivered by Jarvis. These types of datasets justify this performance. Specifically, DS3 and DS4 contain a high number of identical sequences. These are collection of mitogenomes, archeal and virus where the variability is very low, which gives an advantage to models of extremely repetitive nature. Such models, also known as weighted stochastic repeat models, are present in Jarvis, unlike in GeCo3. The reason why we excluded the inclusion of these models in GeCo is that they fail in scalability because the RAM increases according to the sequence length. For the larger datasets, DS1 and DS2, Jarvis was unable to compress the sequences even with 32 GB of RAM. On the other hand, GeCo3 has constant RAM, which is not affected by the sequence length but rather only by the mode used.

Comparing GeCo3 against the second best compressor for

each dataset, the compression gain is 6% (vs GeCo2), 5.8% (vs GeCo2), −0.8% (vs Jarvis), −3.2% (vs Jarvis) and 1.9% (vs Jarvis), for DS1, DS2, DS3, DS4 and DS5, respectively. For the individual sequences in the datasets, GeCo3 compresses more than the other compressors, except for the AgPh, BuEb, Mito, Virus and Archaea. Tiny sequences compose the AgPh and BuEb dataset, and the neural network does not have enough time to learn, while Mito, Virus and Archaea have already been mentioned above.

Regarding computational time, GeCo3 is faster than XM per dataset, spending on the average only 0.6× the time. Against GeCo2, it is slower 2.1× on average, and compared to Jarvis, it is 1.1× slower. NAF is the fastest compressor in the benchmark. Compared to NAF, GeCo3 is between 12× slower for DS5 and 3× for DS1.

Regarding computational memory, the maximum amount of RAM used for GeCo2 and GeCo3 was 12.6GB, Jarvis peaked at 32GB, XM at 8GB, and NAF used at most 0.7GB. Jarvis could not complete the compression for DS1 and DS2 due to a lack of memory. This issue is a limitation that was mentioned earlier. We also note that the XM is unable to decompress some of the sequences. In these cases, the decompressed file has the correct size, but the sequence does not fully match the original file. NAF, GeCo2, and GeCo3 were the only compressors that have been able to compress all the sequences losslessly, independently from the size. The overall results of these compressors show that GeCo3 provides a total compression improvement of 25% and 6% over NAF and GeCo2, respectively.

Compared with general-purpose compressors that achieve the best compression ratios, such as CMIX and DeepZip, GeCo3 is approximately 100 times faster. GeCo3 also has better total compression ratio compared to CMIX (7.7%). We could not obtain enough results with DeepZip to make a meaningful comparison. The table with the results can be seen in Supplementary Section 3 (Results for general purpose compressors).

Reference-based sequence compression bechmark

In this subsection, we benchmark GeCo3 with state-of-the-art referential compressors. The comparison is done between the genomes of different species and not for re-sequenced genomes. Re-sequencing is applied to the same species and, in a general case, limits the domain of applications; for example, phylogenomic, phylogenetic, or evolutionary analysis.

To run the experiments, we used four complete genomes of closely related species: *Homo sapiens* (HS), *Pan troglodytes* (PT), *Gorilla gorilla* (GG) and *Pongo abelii* (PA). The compression for PT, GG, and PA was done using HS as the reference. HS was compressed using GG as a reference. Each chromosome was paired with the corresponding one of the other species. Due to the unavailability of chromosome Y for GG and PA, comparisons that involved these chromosomes were not made. The compressors used in this benchmark are GeCo3, GeCo2, iDoComp [71], GDC2 [72], and HRCM [81]. The FASTA files were filtered such that the resulting file only contained the symbols $\{A, C, G, T\}$, and a tiny header line. HRCM needs the line size to be limited; therefore, line breaks were added for the files under its compression. However, this approach prevents a direct comparison of total compressed size and time, which we solved using the compression ratio percentage ($output\_size \div input\_size \times 100$) and the speed in kilobytes per seconds ($input\_size \div 1000 \div seconds\_spent$). For GeCo2 and GeCo3, two approaches of referential compression are considered. One approach is based on conditional compression, where a hybrid of both reference and target models are used. The other approach, called relative approach, uses exclusively models loaded from the reference sequence. Both types of compression assume causality, which means that with the respective reference sequence, the decompressor is able to decompress without loss. The reason why we benchmark these two approaches is that there are many sequence analysis applications for both approaches.

The results are presented in Table 4, showing the total compression ratio and speed for the four comparisons. The total compression ratio is the $total\_output\_size \div total\_input\_size \times 100$ and the total speed is $total\_input\_size \div 1000 \div total\_seconds\_spent$. The results show GeCo3 achieving the best compression ratio, both in relative and conditional compression. The latter shows improved compression capabilities, with average improvements of 11%, 35%, 38% and 50% over GeCo2, iDoComp, GDC2 and HRCM, respectively. This comes at a cost of being the slowest. The average increase in time over GeCo2, iDoComp, GDC2 and HRCM is 1.7×, 9.8×, 2.6× and 7.3×, respectively. Compared with GeCo2, the total improvement for PT, PA, GG, and HS is 12.4%, 11.7%, 10.8% and 10.1%. The total improvements are similar to the average improvement per chromosome. The computational RAM of GeCo3 is similar to GeCo2. The complete results per chromosome are shown in Supplementary Section 4 (Complete results for referential compression). These show that in the majority of pairs GeCo3 offers better compression.

In Table S7 of Supplementary Section 4, we show the results for compression of a re-sequenced genome. In this dataset HRCM achieves the best results, with GeCo3 trailing both in speed (42×) and ratio (−363%). While these results show that GeCo2 and GeCo3 are not suitable for compressing this type of dataset, the substantial improvement over GeCo2 (20%), hint at the possibility that the new mixer might be useful when integrated into a different type of compressor.

Estimating the cost for long term storage

To estimate the cost of long term storage, we developed a model with the following simplifying assumptions:

- two or more copies are stored;
- compression is done once and the result is copied to the different backup media;
- one CPU core is at 100% utilization during compression;
- the cooling and transfer costs are ignored;
- the computing platform is idle when not compressing;
- no human operator is waiting for the operations to terminate.

Given the assumptions we now show the cost model:

$$Total_{cost} = Processing_{cost} + Storage_{cost}$$
$$Processing_{cost} = Processing_{time} \times Power \times Energy_{price}$$
$$Storage_{cost} = N_{copies} \times Size \times Size_{price},$$

where $Processing_{time}$ is the total time to compress and decompress the sequence.

From [94], we use the single thread load subtracted by the idle value to calculate the power (watts) a system uses during processing. The average result for all systems is 34 watts. The average cost of electricity in the world is 12 cents per kWh, according to [95]. The average storage costs per GB for HDDs is 4 cents [96] and for SSDs is 13 cents [97].

Assuming 13 cents per GB and three copies, the costs for DS1 are 11.86€, 9.54€ and 9.5€ for NAF, GeCo2, and GeCo3, respectively. Using 4 cents per GB and three copies, GeCo2 is more cost effective at 3.12€, followed by GeCo3 (3.46€) and NAF (3.74€). In Fig. 3, we show the costs of storing each sequence in DS1 and DS2 with GeCo3 relative to NAF and GeCo2.

Table 3. Size and time needed to represent a DNA sequence for NAF, XM, Jarvis, GeCo2, and GeCo3. For DS5, Jarvis uses the same configuration as in [65], for DS4 and DS3 it uses level 7. XM uses the default configuration. NAF uses the highest compression level (22). GeCo2 and GeCo3 use mode 16 for DS5, except for BuEb, AgPh and YeMi which use the configurations of Table 1. For DS4 and DS3 the models are *"-tm 3:1:1:1:0.8/0:0:0 -tm 6:1:1:1:0.85/0:0:0 -tm 9:1:1:1:0.85/0:0:0 -tm 12:10:0:1:0.85/0:0:0 -tm 15:200:1:10:0.85/2:1:0.85 -tm 17:200:1:10:0.85/2:1:0.85 -tm 20:500:1:40:0.85/5:20:0.85"*, DS2 uses *"-tm 3:1:1:1:0.70/0:0:0 -tm 8:1:1:1:0.85/0:0:0 -tm 13:10:0:1:0.85/0:0:0 -tm 19:500:1:40:0.85/5:20:0.85"*, and Virome uses *"-tm 7:1:1:1:0.8/0:0:0 -tm 13:10:0:1:0.95/0:0:0 -tm 19:500:1:40:0.95/5:20:0.95"*. Denisova uses the same models as Virome but with inversions turned off. GeCo3 uses a learning rate of 0.03 and 64 hidden nodes for all sequences. The character '*' indicates the sequence was not compressed due to an error and '/' due to out of memory. Results where the decompression produces different results than the input file are appended by the character '?'.

		NAF		XM		Jarvis		GeCo2		GeCo3	
DS	ID	size	time	size	time	size	time	size	time	size	time
1	Denisova	25.36 GB	25h22m	*	*	/	/	20.61 GB	23h18m	**19.55 GB**	71h19m
	Virome	4.72 GB	6h01m	*	*	/	/	3.17 GB	8h45m	**2.79 GB**	24h32m
	Total	30.08 GB	31h23m	*	*	/	/	23.78 GB	32h04m	**22.34 GB**	95h51m
2	PiAbC	2.29 GB	2h45m	*	*	/	/	1.86 GB	4h02m	**1.71 GB**	9h21m
	HoSaC	634.07 MB	38m	*	*	/	/	579.66 MB	53m12s	**560.88 MB**	2h14m
	PaTrC	619.48 MB	37m	*	*	/	/	569.40 MB	51m40s	**551.54 MB**	2h08m
	GoGoC	603.39 MB	36m	*	*	/	/	556.54 MB	49m57s	**539.30 MB**	2h04m
	Total	4.15 GB	4h36m	*	*	/	/	3.57 GB	6h37m	**3.36 GB**	15h49m
3	Archaea	128.09 MB	7m	103.01 MB?	1h41m	**96.66 MB**	57m	103.70 MB	30m	97.87 MB	55m
	Virus	85.51 MB	6m	63.93 MB?	1h35m	**61.19 MB**	1h35m	65.63 MB	29m	61.19 MB	55m
	Total	213.60 MB	14m	166.93 MB?	3h16m	**157.84 MB**	2h32m	169.34 MB	1h00m	159.07 MB	1h51m
4	Mito	35.93 MB	2m32s	28.12 MB?	47m11s	**27.11 MB**	16m1s	30.40 MB	11m26s	28.17 MB	21m31s
	HoSaY	5.17 MB	11s	3.88 MB?	3m25s	3.93 MB	1m45s	4.08 MB	1m15s	**3.85 MB**	2m21s
	Total	41.10 MB	2m43s	32.01 MB?	50m36s	**31.04 MB**	17m46s	34.48 MB	12m41s	32.03 MB	23m52s
5	HoSa	41.73 MB	2m06s	38.66 MB?	29m26s	38.66 MB	4m33s	38.79 MB	11m17s	**37.56 MB**	22m39s
	GaGa	35.57 MB	1m38s	33.83 MB?	22m20s	33.70 MB	2m38s	33.75 MB	8m43s	**33.26 MB**	17m38s
	DaRe	12.83 MB	32s	11.17 MB?	8m59s	11.17 MB	1m32s	11.44 MB	3m40s	**10.97 MB**	7m32s
	OrSa	9.53 MB	21s	8.48 MB?	6m39s	8.45 MB	1m14s	8.60 MB	2m37s	**8.34 MB**	5m17s
	DrMe	7.85 MB	15s	7.53 MB?	5m01s	7.49 MB	22s	7.47 MB	1m57s	**7.36 MB**	3m50s
	EnIn	5.87 MB	12s	5.12 MB?	3m19s	5.09 MB	36s	5.14 MB	1m37s	**5.02 MB**	3m12s
	ScPo	2.59 MB	4s	2.53 MB	55s	2.52 MB	11s	2.52 MB	44s	**2.51 MB**	1m21s
	PlFa	2.02 MB	4s	1.92 MB	59s	1.92 MB	10s	1.93 MB	37s	**1.90 MB**	1m09s
	EsCo	1.15 MB	2s	1.11 MB	13s	1.10 MB	4s	1.10 MB	24s	**1.09 MB**	39s
	HaHi	948.69 KB	2s	914.87 KB	16s	899.47 KB	2s	899.17 KB	21s	**889.51 KB**	34s
	AeCa	396.82 KB	1s	387.00 KB	3s	380.51 KB	1s	381.29 KB	13s	**376.97 KB**	18s
	HePy	404.55 KB	1s	384.30 KB	4s	374.37 KB	1s	375.66 KB	13s	**371.62 KB**	19s
	YeMi	17.35 KB	1s	16.84 KB	0s	16.87 KB	0s	16.80 KB	0s	**16.79 KB**	0s
	AgPh	11.02 KB	1s	10.71 KB	0s	10.75 KB	0s	**10.71 KB**	0s	10.72 KB	0s
	BuEb	4.81 KB	1s	**4.64 KB**	0s	4.70 KB	0s	4.69 KB	0s	4.69 KB	0s
	Total	120.94 MB	5m22s	112.07 MB	1h18m14s	111.79 MB	11m24s	112.42 MB	32m23s	**109.68 MB**	1h04m28s

Table 4. Total referential compression ratio and speed in kB/s. GeCo3 uses 64 hidden nodes and has 0.03 learning rate. The configuration for GeCo2-r and GeCo3-r (relative approach) is *"-rm 20:500:1:35:0.95/3:100:0.95 -rm 13:200:1:1:0.95/0:0:0 -rm 10:10:0:0:0.95/0:0:0"*. For GeCo2-h and GeCo3-h (conditional approach) the following models where added *"-tm 4:1:0:1:0.9/0:0:0 -tm 17:100:1:10:0.95/2:20:0.95"*. iDoComp, GDC2 and HRCM use the default configuration.

		HRCM		GDC2		iDoComp		GeCo2-r		GeCo3-r		GeCo2-h		GeCo3-h	
DSR	ID	ratio	speed	ratio	speed	ratio	speed	ratio	speed	ratio	speed	ratio	speed	ratio	speed
1	HSxPT	6.29	2,006	5.01	841	4.78	2,430	4.16	527	3.65	296	4.02	374	**3.52**	224
2	HSxPA	15.27	1,260	12.24	382	11.31	1,891	7.51	513	6,57	294	7.26	367	**6.41**	222
3	HSxGG	8.80	1,691	7.06	588	6.70	2,201	5.58	516	4.96	293	5.43	369	**4.84**	222
4	GGxHS	9.48	1,773	8.11	712	7.80	2,332	6.43	558	5.81	301	5.77	389	**5.19**	230
	Total	9.96	1,635	8.11	580	7.66	2,195	5.92	529	5.26	296	5.62	375	**4.99**	225

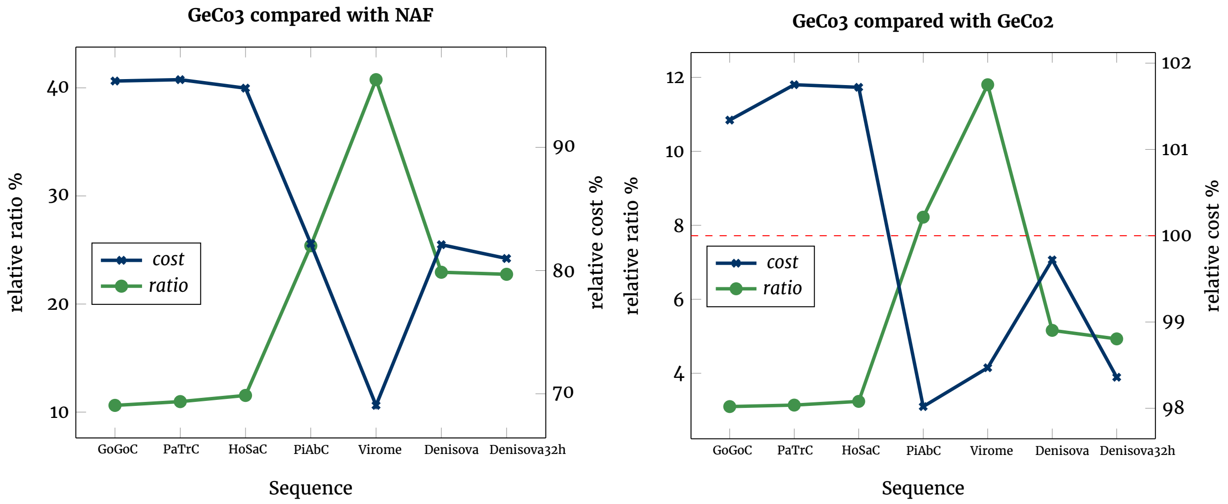

Figure 3. Relative ratio and cost of GeCo3 compared with NAF and GeCo2 for sequences in DS1 and DS2. Higher relative ratios represent greater compression improvements by GeCo3. The cost is calculated assuming 13 cents per GB and the storage of three copies. The red dashed line shows the cost threshold. Cost points above the line indicate that GeCo3 is more expensive. Denisova32h represents the results of running the Denisova sequence with 32 instead of 64 hidden nodes.

As hinted by Fig. 2, we also show that the cost of compressing the Denisova sequence is improved when using 32 instead of 64 hidden nodes. The reduction of hidden nodes leads to a negligible drop in compression ratio (5.2% to 4.9% vs GeCo2), but a substantial time decrease (3.1× to 2.4× vs GeCo2).

These results use the average costs, though given the variability of electricity prices, CPU power efficiency and storage costs, the analysis would need to be done for each specific case.

Discussion

In essence, this article considers the GeCo2 as a base, collecting its specific DNA models, and augments the mixture of models by using a neural network. The primary outcome is a new efficient tool, GeCo3. The results show a compression improvement at the cost of longer execution times and equivalent RAM.

For the evaluated datasets, this approach delivers the best results for the most significant and the highest repetitive sequences. One of the reasons for this is that for small sequences, the network spends a significant percentage of time adjusting. Moreover, we show the importance of selecting and deriving the appropriate network inputs as well as the influence of the number of hidden nodes. These can be used to increase compression at the cost of higher execution times.

Compared to other state-of-the-art compressors, this approach is typically slower, but achieves better compression ratios both in reference-free and referential compression. Nevertheless, the compression times can be reduced by decreasing the number of hidden nodes while still improving the ratio.

The GeCo3 reference-free results, show an improvement of 25%, and 6% over NAF and GeCo2, respectively. In reference-based compression, GeCo3 is able to provide compression gains of 11%, 35%, 38%, and 50% over GeCo2, iDoComp, GDC2, and HRCM, respectively.

The time trade-off and the symmetry of compression-decompression establish GeCo3 as a non-appropriate tool for on-the-fly decompression. Tools such as NAF [68] are efficient for this purpose, namely because the computational decompression speed is very high, which for industrial usage is mandatory. The purposes of tools such as GeCo3 are in another domain, namely long-term storage and data analysis.

In particular, the results suggest that long-term storage of extensive databases, for example, as proposed in [98], would be a good fit for GeCo3.

The steady rise of analysis tools based on DNA sequence compression is showing its potential, with increasing applications and surprising results. Some of the applications are the estimation of the Kolmogorov complexity of genomes [99], rearrangements detection [100], sequence clustering [101], measurement of distances and phylogenetic trees computation [102], and metagenomics [12].

The main advantage of using efficient (lossless) compression-based data analysis is non-overestimation. Many analysis algorithms include multiple thresholds that use a consensus value for what is considered balanced and consistent, leaving space for overestimation. The problem is that using a consensus or average parameter for a specific analysis may overtake the limit of the estimation balance. Since data compression needs the appropriate decompressor to ensure the full recovery of the data, the compressor acts under a boundary that ensures that the limit is never overpassed (Kolmogorov complexity). This property is critical in data analysis because the data in use may be vital and sensitive, mainly when multiple models are used. Without a channel information limit and an efficient mixing model, the information that is embedded in the probabilities estimation of each model transits to the model choice.

The mixing method used to achieve these results assumes only that probabilities for the symbols are available. Because of this, it permits to be easily exported to other compressors or compressed-based data analysis tools that use multiple models. GeCo3 shows what compression improvements and execution times can be expected when using neural networks for the mixture of experts in DNA sequence compression.

This paper highlights the importance of expert mixing. Mixing has applications in all areas where there is the uncertainty of outcomes, and many expert opinions are available. This ranges from compression to climate modeling and, in the future, possibly the creation of legislation. While more traditional methods, such as weighted majority voting, are more efficient and can achieve accurate results, neural networks show promising results. With the development of specialized hardware instructions and data-types to be included in general-purpose CPUs [103, 104], neural networks should become an even more attractive option for expert mixing.

One of the possible reasons this approach has higher compression than GeCo2 is due to the mixing output not being constrained by the inputs. By comparing the histograms in

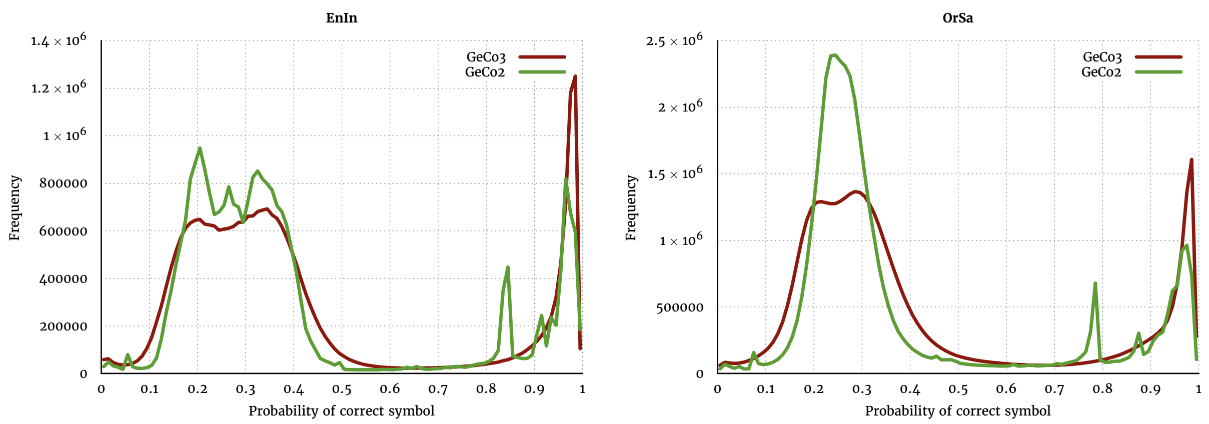

Figure 4. Comparison of histograms using the EnIn (*Entamoeba invadens*) and OrSa (*Oryza sativa*) genome sequences and the GeCo2 and GeCo3 as data compressors.

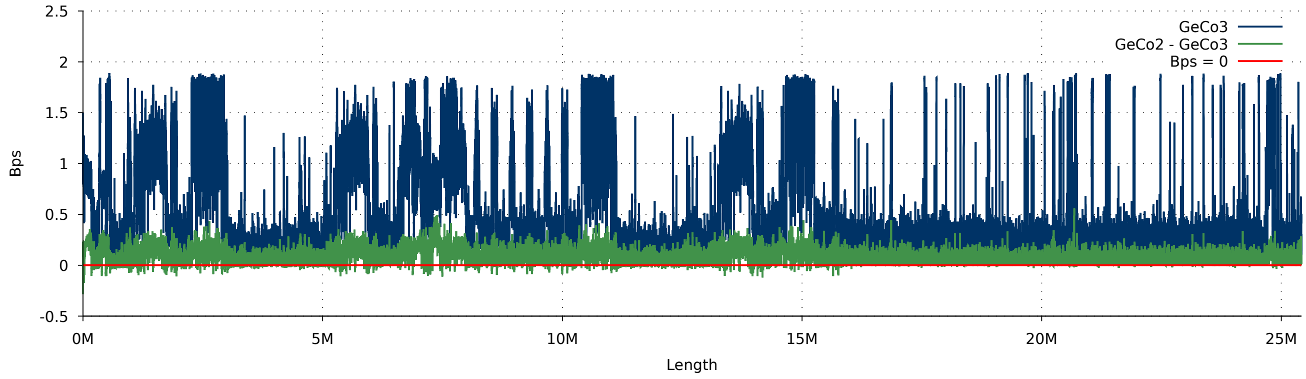

Figure 5. Complexity profile using the smoothed number of bits per symbol (Bps) of GeCo2 subtracted by GeCo3 Bps. The Bps were obtained by referential compression of PT_Y (Chromosome Y from *Pan troglodytes*) with the corresponding *Homo sapiens* chromosome, with the same parameters as in Table 4. Regions where the line rises above zero indicate that GeCo3 compresses more than GeCo2.

Fig. 4 for the sequence EnIn and OrSa (two of the sequences with higher gains), we can verify that GeCo3 appears to correct the models' probabilities greater than 0.8 to probabilities closer to 0.99. Therefore, in some way, it is betting more if at least four in five chances are accomplished. Referential histograms are presented in Supplementary Section 7 (Referential histograms); these are similar to the ones presented here.

Another improvement is due to the higher percentage of symbols inferred correctly. For dataset five (DS5), GeCo3 has an average improvement of 1.5% in the number of symbols inferred correctly, where only the smallest sequence has a lower hit rate than GeCo2. Supplementary Section 2 (Percentage of symbols guessed correctly), presents the table of hit rate per sequence.

For referential compression, we show a complexity profile in Fig. 5. This profile reveals that GeCo3 consistently outputs a lower number of bits per symbol. The gains appear to be larger in places of higher sequence complexity, namely in the higher Bps regions. These regions are typically where rapid switching between smaller models should occur, suggesting that the neural network mixer can adapt faster than the approach used in GeCo2. Supplementary Section 5 (Referential complexity profiles), presents two additional complexity profiles with similar nature.

Finally, the training is maintained during the entire sequence. Because, we found that doing early stopping leads to worse outcomes. This characteristic might be due to the advantages of over-fitting for non-stationary time series reported in [105].

Additional improvements on the compression of large FASTQ data, for example, from the Virome and Denisova datasets can be achieved with complementary techniques based on reordering or metagenomic composition identification. Specifically, the reads of these datasets can be splited according to their composition using fast assembly-free and alignment-free methods, namely extensions of Read-SpaM [106], in order to take advantage of the similarity read proximity to improve substantially the compression.

Whichever the technology and application, the core method that we provide here, namely for combining the specific DNA models with neural networks, enables a substantial improvement in the precision of DNA sequence compression-based data analysis tools and provides a significant reduction of storage associated with DNA sequences.

Availability of source code and requirements

- Project name: GeCo3
- Project home page: http://github.com/cobilab/geco3
- RRID: SCR_018877
- biotools: geco3
- Operating system(s): Platform independent
- Programming language: C
- Other requirements: C compiler(e.g. gcc)
- License: GNU GPL

Availability of supporting data and materials

Supplementary material includes all the information to install the benchmark compressors, download and compress the data.

Competing Interests

The author(s) declare that they have no competing interests

Funding

This work is partially funded by the national funds through the FCT in the context of the project UIDB/00127/2020. D.P. is funded by national funds through FCT - Fundação para a Ciência e a Tecnologia, I.P., under the Scientific Employment Stimulus - Institutional Call - CI-CTTI-94-ARH/2019.

Author's Contributions

M.S., D.P., A.J.P. conceived and designed the experiments; M.S. implemented the algorithm; M.S. performed the experiments; M.S., D.P., A.J.P. analyzed the data; M.S., D.P., A.J.P. wrote the paper.

References

1. Stephens ZD, Lee SY, Faghri F, Campbell RH, Zhai C, Efron MJ, et al. Big data: astronomical or genomical? PLoS biology 2015;13(7):e1002195.
2. Pratas D, Pinho AJ, Ferreira PJ. Efficient compression of genomic sequences. In: 2016 Data Compression Conference (DCC) IEEE; 2016. p. 231–240.
3. Pratas D, Hosseini M, Pinho AJ. GeCo2: An Optimized Tool for Lossless Compression and Analysis of DNA Sequences. In: International Conference on Practical Applications of Computational Biology & Bioinformatics Springer; 2019. p. 137–145.
4. Mahoney M, Data Compression Explained;. http://mattmahoney.net/dc/dce.html.
5. Pratas D, Hosseini M, Pinho AJ. Substitutional tolerant Markov models for relative compression of DNA sequences. In: International Conference on Practical Applications of Computational Biology & Bioinformatics Springer; 2017. p. 265–272.
6. Polikar R. Ensemble based systems in decision making. IEEE Circuits and systems magazine 2006;6(3):21–45.
7. Wolpert DH. Stacked generalization. Neural networks 1992;5(2):241–259.
8. Khalid S, Khalil T, Nasreen S. A survey of feature selection and feature extraction techniques in machine learning. In: 2014 Science and Information Conference IEEE; 2014. p. 372–378.
9. Fritz MHY, Leinonen R, Cochrane G, Birney E. Efficient storage of high throughput DNA sequencing data using reference-based compression. Genome research 2011;21(5):734–740.
10. Giancarlo R, Scaturro D, Utro F. Textual data compression in computational biology: a synopsis. Bioinformatics 2009;25(13):1575–1586.
11. Pratas D, Silva RM, Pinho AJ, Ferreira PJ. An alignment-free method to find and visualise rearrangements between pairs of DNA sequences. Scientific reports 2015;5(1):1–9.
12. Pratas D, Pinho AJ. Metagenomic composition analysis of sedimentary ancient DNA from the Isle of Wight. In: 2018 26th European Signal Processing Conference (EUSIPCO) IEEE; 2018. p. 1177–1181.
13. Covas C, Caetano T, Cruz A, Santos T, Dias L, Klein G, et al. Pedobacter lusitanus sp. nov., isolated from sludge of a deactivated uranium mine. International journal of systematic and evolutionary microbiology 2017;67(5):1339–1348.
14. Pyöriä L, Jokinen M, Toppinen M, Salminen H, Vuorinen T, Hukkanen V, et al. HERQ-9 Is a New Multiplex PCR for Differentiation and Quantification of All Nine Human Herpesviruses. Msphere 2020;5(3).
15. Toppinen M, Pratas D, Väisänen E, Söderlund-Venermo M, Hedman K, Perdomo MF, et al. The landscape of persistent human DNA viruses in femoral bone. Forensic Science International: Genetics 2020;p. 102353.
16. Duggan AT, Perdomo MF, Piombino-Mascali D, Marciniak S, Poinar D, Emery MV, et al. 17th century variola virus reveals the recent history of smallpox. Current Biology 2016;26(24):3407–3412.
17. Teixeira H, Berg T, Uusitalo L, Fürhaupter K, Heiskanen AS, Mazik K, et al. A catalogue of marine biodiversity indicators. Frontiers in Marine Science 2016;3:207.
18. Cowan DA, Ramond JB, Makhalanyane TP, De Maayer P. Metagenomics of extreme environments. Current opinion in microbiology 2015;25:97–102.
19. Rieseberg LH. Chromosomal rearrangements and speciation. Trends in ecology & evolution 2001;16(7):351–358.
20. Roeder GS, Fink GR. DNA rearrangements associated with a transposable element in yeast. Cell 1980;21(1):239–249.
21. Sajantila A, Editors' Pick: Contamination has always been the issue! BioMed Central; 2014.
22. Harris K. Evidence for recent, population-specific evolution of the human mutation rate. Proceedings of the National Academy of Sciences 2015;112(11):3439–3444.
23. Jeong C, Di Rienzo A. Adaptations to local environments in modern human populations. Current opinion in genetics & development 2014;29:1–8.
24. Beres S, Kachroo P, Nasser W, Olsen R, Zhu L, Flores A, et al. Transcriptome remodeling contributes to epidemic disease caused by the human pathogen. Streptococcus pyogenes 2016;p. 00403–16.
25. Fumagalli M, Sironi M. Human genome variability, natural selection and infectious diseases. Current opinion in immunology 2014;30:9–16.
26. Long H, Sung W, Kucukyildirim S, Williams E, Miller SF, Guo W, et al. Evolutionary determinants of genome-wide nucleotide composition. Nature ecology & evolution 2018;p. 1.
27. Golan A. Foundations of Info-Metrics: Modeling and Inference with Imperfect Information. Oxford University Press; 2017.
28. Hernaez M, Pavlichin D, Weissman T, Ochoa I. Genomic Data Compression. Annual Review of Biomedical Data Science 2019;2:19–37.
29. Hosseini M, Pratas D, Pinho AJ. A survey on data compression methods for biological sequences. Information 2016;7(4):56.
30. Grumbach S, Tahi F. Compression of DNA sequences; 1993. p. 340–350.
31. Grumbach S, Tahi F. A new challenge for compression algorithms: genetic sequences 1994;30(6):875–886.
32. Rivals E, Delahaye JP, Dauchet M, Delgrange O. A guaranteed compression scheme for repetitive DNA sequences; 1996. p. 453.
33. Loewenstern D, Yianilos PN. Significantly lower entropy estimates for natural DNA sequences; 1997. p. 151–160.
34. Allison L, Edgoose T, Dix TI. Compression of strings with approximate repeats. In: Proc. of Intelligent Systems in Molecular Biology, ISMB-98 Montreal, Canada; 1998. p. 8–16.
35. Apostolico A, Lonardi S. Compression of biological sequences by greedy off-line textual substitution; 2000. p. 143–152.

36. Chen X, Li M, Ma B, Tromp J. DNACompress: fast and effective DNA sequence compression 2002;18(12):1696–1698.
37. Matsumoto T, Sadakane K, Imai H. Biological sequence compression algorithms. In: Dunker AK, Konagaya A, Miyano S, Takagi T, editors. Genome Informatics 2000: Proc. of the 11th Workshop Tokyo, Japan; 2000. p. 43–52.
38. Tabus I, Korodi G, Rissanen J. DNA sequence compression using the normalized maximum likelihood model for discrete regression; 2003. p. 253–262.
39. Korodi G, Tabus I. An efficient normalized maximum likelihood algorithm for DNA sequence compression 2005 Jan;23(1):3–34.
40. Cherniavsky N, Ladner R. Grammar-based compression of DNA sequences. University of Washington; 2004.
41. Manzini G, Rastero M. A simple and fast DNA compressor 2004;34:1397–1411.
42. A J T Lee CC, Chen C. DNAC: An Efficient Compression Algorithm for DNA Sequences. National Taiwan University, Taipei, Taiwan 10617, ROC 2004;1(1).
43. Behzadi B, Le Fessant F. DNA compression challenge revisited. In: Combinatorial Pattern Matching: Proc. of CPM-2005, vol. 3537 of LNCS Jeju Island, Korea: Springer-Verlag; 2005. p. 190–200.
44. Cao MD, Dix TI, Allison L, Mears C. A simple statistical algorithm for biological sequence compression; 2007. p. 43–52.
45. Vey G. Differential direct coding: a compression algorithm for nucleotide sequence data. Database 2009;2009.
46. Mishra KN, Aaggarwal A, Abdelhadi E, Srivastava D. An efficient horizontal and vertical method for online dna sequence compression. International Journal of Computer Applications 2010;3(1):39–46.
47. Rajeswari PR, Apparao A. GENBIT Compress-Algorithm for repetitive and non repetitive DNA sequences. International Journal of Computer Science and Information Technology 2010;2:25–29.
48. Gupta A, Agarwal S. A novel approach for compressing DNA sequences using semi-statistical compressor. International Journal of Computers and Applications 2011;33(3):245–251.
49. Zhu Z, Zhou J, Ji Z, Shi Y. DNA sequence compression using adaptive particle swarm optimization-based memetic algorithm 2011;15(5):643–658.
50. Pinho AJ, Pratas D, Ferreira PJSG. Bacteria DNA sequence compression using a mixture of finite-context models. In: Proc. of the IEEE Workshop on Statistical Signal Processing Nice, France; 2011. .
51. Pinho AJ, Ferreira PJSG, Neves AJR, Bastos CAC. On the representability of complete genomes by multiple competing finite-context (Markov) models 2011;6(6):e21588.
52. Roy S, Khatua S, Roy S, Bandyopadhyay SK. An efficient biological sequence compression technique using lut and repeat in the sequence. arXiv preprint arXiv:12095905 2012;.
53. Satyanvesh D, Balleda K, Padyana A, Baruah P. GenCodex - A Novel Algorithm for Compressing DNA sequences on Multi-cores and GPUs. In: Proc. IEEE, 19th International Conf. on High Performance Computing (HiPC), Pune, India; 2012. .
54. Bose T, Mohammed MH, Dutta A, Mande SS. BIND–An algorithm for loss-less compression of nucleotide sequence data. Journal of Biosciences 2012;37(4):785–789.
55. Li P, Wang S, Kim J, Xiong H, Ohno-Machado L, Jiang X. DNA-COMPACT: DNA Compression Based on a Pattern-Aware Contextual Modeling Technique 2013;8(11):e80377.
56. Pratas D, Pinho AJ. Exploring deep Markov models in genomic data compression using sequence pre-analysis; 2014. p. 2395–2399.
57. Sardaraz M, Tahir M, Ikram AA, Bajwa H. SeqCompress: An algorithm for biological sequence compression. Genomics 2014;104(4):225–228.
58. Guo H, Chen M, Liu X, Xie M. Genome compression based on Hilbert space filling curve. In: Proceedings of the 3rd International Conference on Management, Education, Information and Control (MEICI 2015), Shenyang, China; 2015. p. 29–31.
59. Xie X, Zhou S, Guan J. CoGI: Towards compressing genomes as an image. IEEE/ACM transactions on computational biology and bioinformatics 2015;12(6):1275–1285.
60. Chen M, Shao J, Jia X. Genome sequence compression based on optimized context weighting. Genetics and molecular research: GMR 2017;16(2).
61. Bakr NS, Sharawi AA. Improve the compression of bacterial DNA sequence. In: 2017 13th International Computer Engineering Conference (ICENCO) IEEE; 2017. p. 286–290.
62. Mansouri D, Yuan X. One-Bit DNA Compression Algorithm. In: International Conference on Neural Information Processing Springer; 2018. p. 378–386.
63. Wang R, Bai Y, Chu YS, Wang Z, Wang Y, Sun M, et al. DeepDNA: a hybrid convolutional and recurrent neural network for compressing human mitochondrial genomes. In: 2018 IEEE International Conference on Bioinformatics and Biomedicine (BIBM) IEEE; 2018. p. 270–274.
64. Wang R, Zang T, Wang Y. Human mitochondrial genome compression using machine learning techniques. Human genomics 2019;13(1):1–8.
65. Pratas D, Hosseini M, Silva JM, Pinho AJ. A Reference-Free Lossless Compression Algorithm for DNA Sequences Using a Competitive Prediction of Two Classes of Weighted Models. Entropy 2019;21(11):1074.
66. Mohammed MH, Dutta A, Bose T, Chadaram S, Mande SS. DELIMINATE—a fast and efficient method for loss-less compression of genomic sequences: sequence analysis. Bioinformatics 2012;28(19):2527–2529.
67. Pinho AJ, Pratas D. MFCompress: a compression tool for FASTA and multi-FASTA data. Bioinformatics 2014;30(1):117–118.
68. Kryukov K, Ueda MT, Nakagawa S, Imanishi T. Nucleotide Archival Format (NAF) enables efficient lossless reference-free compression of DNA sequences. Bioinformatics 2019;35(19):3826–3828.
69. Christley S, Lu Y, Li C, Xie X. Human genomes as email attachments. Bioinformatics 2009;25(2):274–275.
70. Brandon MC, Wallace DC, Baldi P. Data structures and compression algorithms for genomic sequence data. Bioinformatics 2009;25(14):1731–1738.
71. Ochoa I, Hernaez M, Weissman T. iDoComp: a compression scheme for assembled genomes. Bioinformatics 2015;31(5):626–633.
72. Deorowicz S, Danek A, Niemiec M. GDC 2: Compression of large collections of genomes. Scientific reports 2015;5:11565.
73. Kuruppu S, Puglisi SJ, Zobel J. Relative Lempel-Ziv compression of genomes for large-scale storage and retrieval. In: International Symposium on String Processing and Information Retrieval Springer; 2010. p. 201–206.
74. Wang C, Zhang D. A novel compression tool for efficient storage of genome resequencing data. Nucleic acids research 2011;39(7):e45–e45.
75. Kuruppu S, Puglisi SJ, Zobel J. Optimized relative Lempel-Ziv compression of genomes. In: Proceedings of the Thirty-Fourth Australasian Computer Science Conference-Volume 113; 2011. p. 91–98.
76. Deorowicz S, Grabowski S. Robust relative compres-

sion of genomes with random access. Bioinformatics 2011;27(21):2979–2986.
77. Pinho AJ, Pratas D, Garcia SP. GReEn: a tool for efficient compression of genome resequencing data. Nucleic acids research 2012;40(4):e27–e27.
78. Wandelt S, Leser U. FRESCO: Referential compression of highly similar sequences. IEEE/ACM Transactions on Computational Biology and Bioinformatics 2013;10(5):1275–1288.
79. Liu Y, Peng H, Wong L, Li J. High-speed and high-ratio referential genome compression. Bioinformatics 2017;33(21):3364–3372.
80. Fan W, Dai W, Li Y, Xiong H. Complementary Contextual Models with FM-Index for DNA Compression. In: 2017 Data Compression Conference (DCC) IEEE; 2017. p. 82–91.
81. Yao H, Ji Y, Li K, Liu S, He J, Wang R. HRCM: An Efficient Hybrid Referential Compression Method for Genomic Big Data. BioMed Research International 2019;2019.
82. Byron K, CMIX;. http://www.byronknoll.com/cmix.html.
83. Goyal M, Tatwawadi K, Chandak S, Ochoa I. DeepZip: Lossless Data Compression using Recurrent Neural Networks. arXiv preprint arXiv:181108162 2018;.
84. Absardi ZN, Javidan R. A Fast Reference-Free Genome Compression Using Deep Neural Networks. In: 2019 Big Data, Knowledge and Control Systems Engineering (BdKCSE) IEEE; 2019. p. 1–7.
85. Robbins H, Monro S. A stochastic approximation method. The annals of mathematical statistics 1951;p. 400–407.
86. Hiransha M, Gopalakrishnan EA, Menon VK, Soman K. NSE stock market prediction using deep-learning models. Procedia computer science 2018;132:1351–1362.
87. Glorot X, Bengio Y. Understanding the difficulty of training deep feedforward neural networks. In: Proceedings of the thirteenth international conference on artificial intelligence and statistics; 2010. p. 249–256.
88. LeCun YA, Bottou L, Orr GB, Müller KR. Efficient backprop. In: Neural networks: Tricks of the trade Springer; 2012.p. 9–48.
89. Pratas D, Toppinen M, Pyöriä L, Hedman K, Sajantila A, Perdomo MF. A hybrid pipeline for reconstruction and analysis of viral genomes at multi-organ level. GigaScience 2020;9:giaa086.
90. Meyer M, Kircher M, Gansauge MT, Li H, Racimo F, Mallick S, et al. A high-coverage genome sequence from an archaic Denisovan individual. Science 2012;338(6104):222–226.
91. Kryukov K, Ueda MT, Nakagawa S, Imanishi T. Sequence Compression Benchmark (SCB) database—a comprehensive evaluation of reference-free compressors for FASTA-formatted sequences. bioRxiv 2019;p. 642553.
92. Pratas D, Pinho AJ. A DNA sequence corpus for compression benchmark. In: International Conference on Practical Applications of Computational Biology & Bioinformatics Springer; 2018. p. 208–215.
93. Ijdo J, Baldini A, Ward D, Reeders S, Wells R. Origin of human chromosome 2: an ancestral telomere-telomere fusion. Proceedings of the National Academy of Sciences 1991;88(20):9051–9055.
94. Hagedoorn H, AMD Ryzen 5 3600 review - Power Consumption and temperatures;. https://www.guru3d.com/articles-pages/amd-ryzen-5-3600-review,7.html.
95. Electricity Prices;. https://www.globalpetrolprices.com/electricity_prices/.
96. Amazon.de HDD prices;. https://diskprices.com/?locale=us&condition=new&units=gb&disk_types=internal_hdd25,internal_sshd,internal_sas.
97. Amazon.de SSD prices;. https://diskprices.com/?locale=us&condition=new&units=gb&disk_types=internal_ssd,m2_ssd,m2_nvme.
98. Lewin HA, Robinson GE, Kress WJ, Baker WJ, Coddington J, Crandall KA, et al. Earth BioGenome Project: Sequencing life for the future of life. Proceedings of the National Academy of Sciences 2018;115(17):4325–4333.
99. Pratas D, Pinho AJ. On the approximation of the Kolmogorov complexity for DNA sequences. In: Iberian Conference on Pattern Recognition and Image Analysis Springer; 2017. p. 259–266.
100. Hosseini M, Pratas D, Morgenstern B, Pinho AJ. Smash++: an alignment-free and memory-efficient tool to find genomic rearrangements. GigaScience 2020 05;9(5).
101. Cilibrasi R, Vitányi PM. Clustering by compression. IEEE Transactions on Information theory 2005;51(4):1523–1545.
102. Li M, Li X, Ma B, Vitányi P. Normalized information distance and whole mitochondrial genome phylogeny analysis. arXiv preprint cs/0111054 2008;.
103. BFLOAT16 – Hardware Numerics Definition;. https://software.intel.com/sites/default/files/managed/40/8b/bf16-hardware-numerics-definition-white-paper.pdf.
104. IBM Reveals Next-Generation IBM POWER10 Processor;. https://newsroom.ibm.com/2020-08-17-IBM-Reveals-Next-Generation-IBM-POWER10-Processor.
105. Kim TY, Oh KJ, Kim C, Do JD. Artificial neural networks for non-stationary time series. Neurocomputing 2004;61:439–447.
106. Lau AK, Dörrer S, Leimeister CA, Bleidorn C, Morgenstern B. Read-SpaM: assembly-free and alignment-free comparison of bacterial genomes with low sequencing coverage. BMC bioinformatics 2019;20(20):638.

Click here to access/download
Supplementary Material
Supplementary Material_r01.pdf

Answers to reviewers

Click here to access/download
Supplementary Material
GeCo3_Answers.pdf

Cover letter
Click here to access/download;Personal Cover;GeCo3_Cover.pdf

Aveiro, May 22, 2020

Dear Editor:

We are pleased to submit the manuscript entitled "**Efficient DNA sequence compression with Neural Networks**" for consideration for publication in GiGaScience as a Technical note.

The increasing production of genomic data has led to an intensifying need for models that can cope efficiently with DNA sequences lossless representation both for storage and compression-based data analysis.

Only a few recent articles propose the use of Neural Networks for DNA sequence compression. **However, they fall short** when compared with specific DNA compression tools, such as GeCo2. This limitation is given by the **absence of models specifically designed to DNA sequences**, for example, the high level of substitutions, rearrangements, contamination, and data heterogeneity.

In this work, **for the first time, we combine the power of Neural Networks with specific DNA models**, proposing a new efficient DNA sequence compressor (GeCo3) both for reference-free and reference-based compression.

We benchmark GeCo3 in eight extensive, fair and diverse datasets achieving a **substantial improvement over the state-of-the-art**, specifically of 2.4%, 6%, 4%, and 5.9% for reference-free, and 12.4%, 11.7%, 10.8% and 10.1% for reference-based compression. The cost is computational time ($2.4\times$ slower that GeCo2) that is minimized for sequences in the GigaByte or higher scales. The RAM is constant, and the tool scales efficiently, independently from the sequence size.

The tool and results are accompanied by a portable code package, requiring only the probabilities of the models as inputs, providing easy exportation to other data compressors or compression-based data analysis tools.

We confirm that this work is original and has not been published elsewhere, nor is it currently under consideration for publication elsewhere. We have no conflicts of interest to disclose.

Thank you for your consideration of this manuscript.

Sincerely,

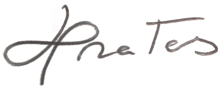

Diogo Pratas, PhD
Department of Electronics,
Telecommunications and Informatics,
University of Aveiro
pratas@ua.pt
+351914670195

Department of Virology
Faculty of Medicine,
University of Helsinki
diogo.pratas@helsinki.fi
