# Supplementary material for: Efficient DNA sequence compression with neural networks
Source: Gigascience. 2020 Nov 11;9(11):giaa119. doi: 10.1093/gigascience/giaa119 (PMC7657843; doi:10.1093/gigascience/giaa119)
Supplement: giaa119_Supplemental_File [file giaa119_supplemental_file.pdf]

Supplementary Material of
"Efficient DNA sequence compression with Neural Networks"

M. Silva, D. Pratas, A. J. Pinho

Contents

1 Stretching function plot **2**

2 Percentage of symbols guessed correctly **2**

3 Results for general purpose compressors **3**

4 Complete results for referential compression **4**

5 Referential complexity profiles **8**

6 Referential hidden nodes effect **9**

7 Referential histograms **10**

8 Reproducibility **10**

8.1 Input data 10
8.1.1 Downloading the genomes 11
8.1.2 Genome identifiers 11
8.1.3 Virome 11
8.1.4 Picea abies 11
8.1.5 Homo sapiens 12
8.1.6 Pan troglodytes 12
8.1.7 Pongo abelii 12
8.1.8 Gorilla gorilla 12
8.2 Compressors 12
8.2.1 Downloading the compressors 12
8.2.2 Running the compressors 13

1 Stretching function plot

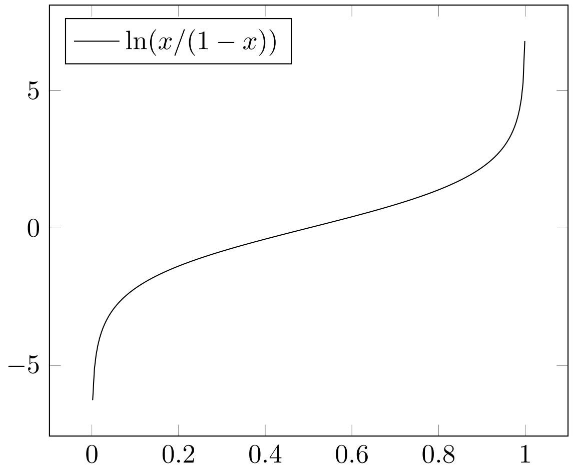

Figure S1: Stretching function applied to the models' probabilities.

2 Percentage of symbols guessed correctly

Table S1: Percentage of symbols guessed correctly by GeCo2 and GeCo3 for all sequences in dataset four (DS4). The improvement percentage of GeCo3 over GeCo2 is the diff.

ID	GeCo2	GeCo3	diff
HoSa	47.1	48.5	2.9
GaGa	38.9	40.0	2.8
DaRe	54.9	55.9	1.8
OrSa	48.2	49.7	3.0
DrMe	38.5	39.7	3.0
EnIn	50.3	51.2	1.8
ScPo	35.8	36.2	1.1
PlFa	44.4	45.3	2.0
EsCo	35.9	36.5	1.6
HaHi	40.0	40.4	1.0
AeCa	36.7	37.3	1.6
HePy	42.2	42.7	1.2
YeMi	41.7	42.1	1.0
AgPh	35.1	35.2	0.3
BuEb	33.2	32.4	-2.5

3 Results for general purpose compressors

Table S2: Number of bytes and time needed to represent a DNA sequence for CMIX, DeepZip and ZPAQ. CMIX and DeepZip were run with the default configuration and ZPAQ was run with level 5. Some tests were not run (NR) due to time constraints and DeepZip forced the computer to reboot (SF) with some sequences.

		CMIX		DeepZip		ZPAQ	
DS	ID	size	time	size	time	size	time
2	PiAbC	NR	NR	NR	NR	2.75 GB	2h01m
	HoSaC	NR	NR	NR	NR	629.76 MB	29m
	PaTrC	NR	NR	NR	NR	614.37 MB	29m
	GoGoC	NR	NR	NR	NR	597.90 MB	28m
	Total	NR	NR	NR	NR	NR	NR
	Archaea	NR	NR	NR	NR	138.05 MB	8m09s
3	Virus	NR	NR	NR	NR	106.40 MB	8m12s
	Total	NR	NR	NR	NR	244.45 MB	16m21s
4	Mito	NR	NR	NR	NR	44.85 MB	2m59s
	HoSaY	4.80 MB	5h17m	SF	SF	5.28 MB	1m07s
	Total	NR	NR	NR	NR	50.13 MB	4m07s
5	HoSa	NR	NR	NR	NR	41.49 MB	2m56s
	GaGa	NR	NR	NR	NR	34.69 MB	2m54s
	DaRe	12.19 MB	12h20m	NR	NR	13.18 MB	2m34s
	OrSa	9.04 MB	8h55m	SF	SF	9.64 MB	1m48s
	DrMe	7.46 MB	6h41m	SF	SF	7.61 MB	1m20s
	EnIn	5.58 MB	5h21m	SF	SF	6.13 MB	1m05s
	ScPo	2.54 MB	2h08m	SF	SF	2.58 MB	25s
	PlFa	1.93 MB	1h48m	SF	SF	1.99 MB	21s
	EsCo	1.09 MB	56m37s	SF	SF	1.11 MB	11s
	HaHi	882.71 KB	46m57s	883.07 KB	1h19m	902.85 KB	9s
	AeCa	370.61 KB	19m01s	371.88 KB	32m41s	380.13 KB	3s
	HePy	376.61 KB	20m03s	377.53 KB	34m49s	384.42 KB	3s
	YeMi	16.68 KB	58s	19.53 KB	1m33s	17.83 KB	0s
	AgPh	10.70 KB	36s	12.24 KB	59s	11.77 KB	0s
	BuEb	4.68 KB	17s	6.23 KB	31s	5.77 KB	0s
	Total	NR	NR	NR	NR	120.13 MB	13m53s

4 Complete results for referential compression

Table S3: Pairwise referential compression ratio and speed in kB/s for PT sequence using HS as reference. GeCo3 uses 64 hidden nodes and has 0.03 learning rate. The configuration for GeCo2-r and GeCo3-r is "-rm 20:500:1:35:0.95/3:100:0.95 -rm 13:200:1:1:0.95/0:0:0 -rm 10:10:0:0:0.95/0:0:0". For GeCo2-h and GeCo3-h the following models where added "-tm 4:1:0:1:0.9/0:0:0 -tm 17:100:1:10:0.95/2:20:0.95". iDoComp, GDC2 and HRCM use the default configuration.

	HRCM		GDC2		iDoComp		GeCo2-r		GeCo3-r		GeCo2-h		GeCo3-h	
ID	ratio	speed	ratio	speed	ratio	speed	ratio	speed	ratio	speed	ratio	speed	ratio	speed
PT_1	4.33	1,260	3.16	889	**2.89**	2,531	4.12	525	3.60	297	4.03	378	3.51	223
PT_2	4.95	1,602	3.68	1,010	**3.36**	2,473	4.09	523	3.57	298	4.01	377	3.49	223
PT_3	4.22	2,067	3.02	1,425	**2.72**	2,630	4.08	528	3.54	301	3.98	381	3.46	225
PT_4	9.47	2,027	7.93	777	7.58	2,154	4.31	539	3.75	297	4.16	376	**3.62**	223
PT_5	11.84	1,745	10.17	523	9.82	1,853	4.03	494	3.54	288	3.95	355	**3.47**	218
PT_6	4.61	2,245	3.40	1,482	**3.13**	2,574	4.04	539	3.51	292	3.95	379	3.45	224
PT_7	5.40	1,743	4.13	887	3.87	2,519	4.27	531	3.75	298	4.11	376	**3.61**	227
PT_8	4.58	2,693	3.27	1,756	**2.96**	2,634	4.19	534	3.67	298	4.07	378	3.56	229
PT_9	7.93	2,121	6.51	702	6.28	2,219	3.97	515	3.50	293	3.90	365	**3.42**	222
PT_10	4.36	2,310	3.16	1,493	**2.92**	2,639	4.00	531	3.52	290	3.91	374	3.43	226
PT_11	4.21	2,735	3.04	1,465	**2.80**	2,648	4.01	534	3.52	300	3.91	375	3.44	226
PT_12	11.70	1,540	10.14	391	9.94	2,044	4.07	536	3.57	299	3.97	379	**3.48**	225
PT_13	4.97	4,093	3.75	2,149	3.54	2,645	3.89	522	3.45	298	3.81	371	**3.35**	227
PT_14	3.97	3,348	2.88	2,069	**2.71**	2,776	3.92	524	3.45	296	3.84	378	3.36	224
PT_15	5.99	2,679	4.75	1,056	4.60	2,515	3.97	530	3.53	292	3.88	373	**3.43**	224
PT_16	6.57	1,904	5.17	680	5.01	2,459	4.48	525	3.98	293	4.34	366	**3.84**	224
PT_17	12.70	1,096	11.27	193	11.29	2,000	4.16	525	3.72	295	4.02	378	**3.56**	223
PT_18	8.41	3,805	7.13	417	6.98	2,306	3.78	520	3.35	297	3.70	374	**3.27**	228
PT_19	5.38	1,258	4.14	630	**4.10**	2,874	5.20	554	4.61	302	4.98	388	4.38	227
PT_20	4.88	3,791	3.76	1,456	**3.65**	2,833	4.53	537	4.08	302	4.32	378	3.84	227
PT_21	4.12	6,096	3.05	2,799	**3.00**	2,704	3.97	490	3.53	281	3.87	350	3.43	213
PT_22	6.51	3,002	5.37	929	5.40	2,583	4.16	497	3.75	286	4.04	358	**3.63**	218
PT_X	3.65	2,833	2.78	1,512	**2.63**	2,670	3.95	540	3.41	297	3.84	373	3.30	224
PT_Y	15.99	4,340	14.92	1,128	15.35	2,082	9.71	534	8.37	296	5.23	368	**4.58**	218
Total	6.29	2,006	5.01	841	4.78	2,430	4.16	527	3.65	296	4.02	374	**3.52**	224

Table S4: Pairwise referential compression ratio and speed in kB/s for PA sequence using HS as reference. Same configurations as in Table 3.

	HRCM		GDC2		iDoComp		GeCo2-r		GeCo3-r		GeCo2-h		GeCo3-h	
ID	ratio	speed	ratio	speed	ratio	speed	ratio	speed	ratio	speed	ratio	speed	ratio	speed
PA_1	26.84	624	24.26	131	23.88	1,340	7.49	528	6.54	297	7.22	370	**6.37**	225
PA_2	18.54	936	15.54	295	14.54	1,670	7.53	511	6.59	296	7.31	370	**6.45**	224
PA_3	20.34	1,116	17.42	299	16.57	1,580	7.34	518	6.40	294	7.13	367	**6.30**	223
PA_4	13.25	1,565	9.94	743	8.59	2,025	7.66	518	6.67	297	7.42	371	**6.53**	225
PA_5	11.47	1,510	8.13	813	6.77	2,178	7.47	516	6.51	297	7.22	369	**6.37**	222
PA_6	10.99	1,465	7.68	906	6.39	2,209	7.36	517	6.42	296	7.13	367	**6.30**	223
PA_7	19.33	1,054	16.42	241	15.59	1,604	7.51	492	6.57	289	7.27	357	**6.41**	217
PA_8	11.98	1,749	8.58	898	7.24	2,170	7.62	514	6.66	295	7.37	367	**6.52**	221
PA_9	17.00	1,423	14.18	352	13.42	1,760	7.19	501	6.31	291	6.97	360	**6.16**	223
PA_10	16.10	1,334	12.99	423	12.02	1,874	7.66	523	6.72	294	7.37	369	**6.54**	223
PA_11	18.51	1,468	15.65	300	14.87	1,697	7.37	506	6.46	293	7.15	366	**6.32**	222
PA_12	10.95	1,299	7.74	685	6.64	2,265	7.46	519	6.53	295	7.24	375	**6.39**	224
PA_13	11.06	2,755	7.82	1,463	6.71	2,286	7.45	524	6.54	296	7.20	373	**6.38**	222
PA_14	10.59	2,073	7.47	1,108	6.52	2,326	7.34	512	6.41	295	7.09	373	**6.26**	221
PA_15	12.45	1,764	9.48	643	8.71	2,142	7.47	512	6.56	294	7.19	373	**6.37**	222
PA_16	13.06	1,276	9.88	411	9.03	1,965	8.00	471	7.04	280	7.68	343	**6.82**	214
PA_17	14.34	899	11.49	228	10.96	1,973	7.53	502	6.65	291	7.30	361	**6.48**	219
PA_18	13.43	2,914	10.44	344	9.59	2,067	7.33	506	6.43	292	7.10	366	**6.31**	221
PA_19	11.96	778	9.11	332	8.77	2,381	8.74	542	7.66	298	8.37	383	**7.39**	223
PA_20	12.00	2,312	9.07	675	8.39	2,215	7.38	506	6.49	290	7.09	367	**6.29**	219
PA_21	11.00	4,233	8.08	1,400	7.52	2,299	8.02	488	7.05	273	7.70	350	**6.85**	212
PA_22	12.91	2,115	10.29	570	10.00	2,232	8.35	506	7.41	288	7.90	359	**7.06**	218
PA_X	9.77	1,734	6.93	914	**6.00**	2,279	7.25	531	6.21	295	6.96	368	6.04	219
Total	6.29	2,006	5.01	841	4.78	2,430	4.16	527	3.65	296	4.02	374	**3.52**	224

Table S5: Pairwise referential compression ratio and speed in kB/s for GG sequence using HS as reference. Same configurations as in Table 3.

	HRCM		GDC2		iDoComp		GeCo2-r		GeCo3-r		GeCo2-h		GeCo3-h	
ID	ratio	speed	ratio	speed	ratio	speed	ratio	speed	ratio	speed	ratio	speed	ratio	speed
GG_1	6.27	1,064	4.58	687	4.15	2,326	4.78	517	4.17	294	4.68	371	**4.08**	223
GG_2	7.12	1,345	5.35	781	4.85	2,312	4.82	526	4.21	296	4.73	374	**4.14**	226
GG_3	5.54	1,766	3.88	1,241	**3.43**	2,489	4.65	530	4.05	297	4.57	375	4.00	220
GG_4	7.66	2,080	5.87	1,096	5.38	2,308	4.86	526	4.25	298	4.75	375	**4.13**	225
GG_5	13.35	1,180	11.48	411	11.17	1,781	10.63	502	9.89	285	10.26	361	**9.59**	220
GG_6	5.50	1,995	3.88	1,460	**3.46**	2,525	4.71	528	4.09	298	4.59	375	4.02	224
GG_7	9.54	1,429	7.71	499	7.31	2,107	4.87	510	4.26	286	4.75	369	**4.16**	220
GG_8	13.67	1,835	11.58	555	11.15	1,905	4.79	526	4.22	295	4.69	371	**4.12**	227
GG_9	12.86	1,761	11.01	426	10.69	1,864	4.62	496	4.05	288	4.52	358	**3.97**	220
GG_10	13.23	1,551	11.26	440	10.91	1,920	4.64	520	4.10	295	4.54	369	**4.00**	221
GG_11	5.95	2,273	4.29	1,111	**3.89**	2,331	4.74	495	4.14	287	4.61	358	4.04	220
GG_12	12.12	1,457	10.26	389	9.97	2,001	4.69	526	4.11	295	4.60	371	**4.02**	224
GG_13	5.24	3,792	3.68	2,403	**3.34**	2,575	4.53	513	4.01	295	4.44	368	3.90	223
GG_14	11.38	2,348	9.66	644	9.42	2,095	4.65	527	4.11	294	4.52	373	**3.99**	221
GG_15	5.24	2,539	3.70	1,537	**3.44**	2,539	4.56	504	4.05	283	4.47	365	3.95	220
GG_16	6.72	1,726	4.94	788	4.65	2,380	5.16	496	4.59	279	5.03	359	**4.45**	219
GG_17	23.13	1,352	21.15	100	20.98	1,618	19.00	588	17.97	314	18.16	404	**17.43**	235
GG_18	9.24	3,549	7.62	399	7.39	2,220	4.50	514	3.99	293	4.39	368	**3.88**	222
GG_19	6.69	1,100	5.08	485	4.98	2,275	5.75	461	5.06	268	5.53	338	**4.86**	209
GG_20	5.66	3,307	4.14	1,425	**3.92**	2,633	4.91	509	4.39	292	4.77	367	4.24	221
GG_21	5.24	5,531	3.77	2,500	**3.62**	2,572	4.66	469	4.15	277	4.56	338	4.04	213
GG_22	5.50	2,922	4.06	1,510	**3.98**	2,576	4.86	468	4.33	278	4.66	343	4.17	211
GG_X	4.89	2,395	3.50	1,427	**3.20**	2,569	4.79	517	4.12	296	4.67	370	4.02	222
Total	8.80	1,691	7.06	588	6.70	2,201	5.58	516	4.96	293	5.43	369	**4.84**	222

Table S6: Pairwise referential compression ratio and speed in kB/s for HS sequence using GG as reference. Same configurations as in Table 3.

ID	HRCM		GDC2		iDoComp		GeCo2-r		GeCo3-r		GeCo2-h		GeCo3-h	
	ratio	speed	ratio	speed	ratio	speed	ratio	speed	ratio	speed	ratio	speed	ratio	speed
HS_1	7.13	1,081	5.69	621	5.27	2,482	5.53	559	4.84	305	5.03	390	**4.44**	230
HS_2	7.48	1,373	5.86	827	5.39	2,362	5.06	543	4.42	301	4.78	376	**4.19**	229
HS_3	5.83	1,801	4.43	1,251	**4.00**	2,534	4.97	544	4.35	301	4.69	380	4.13	227
HS_4	7.82	2,090	6.25	1,159	5.78	2,341	5.19	534	4.68	299	4.81	376	**4.21**	227
HS_5	14.85	1,387	13.20	299	12.83	1,988	12.00	587	11.15	312	11.23	405	**10.59**	236
HS_6	5.70	2,002	4.18	1,438	**3.78**	2,559	4.90	537	4.28	298	4.70	377	4.13	226
HS_7	10.28	1,508	8.71	635	8.39	2,283	5.70	568	5.02	308	5.21	394	**4.63**	233
HS_8	13.79	1,897	12.00	627	11.59	1,974	5.28	539	4.65	299	4.83	382	**4.26**	228
HS_9	14.13	1,900	12.70	541	12.44	2,061	6.68	579	6.12	311	5.08	403	**4.54**	235
HS_10	13.52	1,630	11.77	490	11.40	2,009	5.21	549	4.60	305	4.86	385	**4.31**	228
HS_11	8.08	2,442	6.70	1,184	6.31	2,614	6.66	584	5.88	311	6.11	399	**5.52**	232
HS_12	12.35	1,512	10.82	455	10.61	2,081	5.32	557	4.70	305	4.89	386	**4.32**	228
HS_13	6.16	3,921	4.91	2,248	4.59	2,689	5.53	548	4.97	303	4.96	388	**4.44**	227
HS_14	11.73	2,412	10.33	726	10.14	2,182	5.31	546	4.71	295	4.80	384	**4.24**	229
HS_15	6.56	2,694	5.71	1,304	5.52	2,785	6.06	584	5.79	309	4.88	402	**4.37**	235
HS_16	8.56	1,920	7.22	805	7.01	2,723	6.78	599	6.03	313	5.92	411	**5.35**	235
HS_17	21.51	1,294	20.50	347	20.74	1,512	18.02	514	16.92	295	16.15	373	**15.43**	221
HS_18	9.25	3,850	8.98	1,166	8.89	2,390	6.09	564	7.08	209	4.44	400	**3.96**	233
HS_19	10.50	1,331	9.41	401	9.59	2,924	9.09	668	8.13	329	8.20	446	**7.51**	242
HS_20	6.50	3,494	5.65	1,583	5.53	2,827	5.98	573	5.37	307	5.17	399	**4.64**	230
HS_21	8.20	5,874	7.75	2,022	7.71	2,996	7.96	597	7.28	318	5.72	415	**5.17**	236
HS_22	8.80	3,280	8.06	899	8.21	3,056	7.99	616	7.10	320	6.40	424	**5.82**	239
HS_X	5.33	2,443	4.46	1,503	4.23	2,687	5.43	548	4.83	305	4.79	383	**4.14**	230
Total	9.48	1,773	8.11	712	7.80	2,332	6.43	558	5.81	301	5.77	389	**5.19**	230

Table S7: Total referential compression ratio and speed in kB/s for a re-sequenced Korean human genome. GeCo3 uses 64 hidden nodes and has 0.03 learning rate. The configuration for GeCo2-r and GeCo3-r (relative approach) is "-rm 20:500:1:35:0.95/3:100:0.95 -rm 13:200:1:1:0.95/0:0:0 -rm 10:10:0:0:0.95/0:0:0". For GeCo2-h and GeCo3-h (conditional approach) the following models where added "-tm 4:1:0:1:0.9/0:0:0 -tm 17:100:1:10:0.95/2:20:0.95". iDoComp, GDC2 and HRCM use the default configuration.

HRCM		GDC2		iDoComp		GeCo2-r		GeCo3-r		GeCo2-h		GeCo3-h	
ratio	speed	ratio	speed	ratio	speed	ratio	speed	ratio	speed	ratio	speed	ratio	speed
**0.27**	<u>9,552</u>	0.29	2,620	0.27	3,228	1.55	547	1.30	306	1.55	384	1.24	229

5 Referential complexity profiles

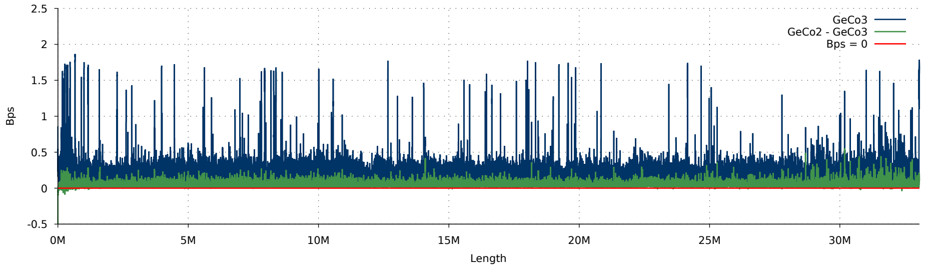

(a) PT_21 - Chromosome 21 from *Pan troglodytes* compressed with the corresponding *Homo sapiens* chromosome.

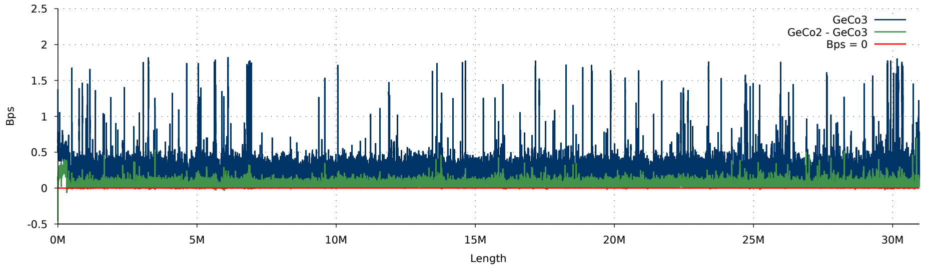

(b) GG_22 - Chromosome 22 from *Gorilla gorilla* compressed with the corresponding *Homo sapiens* chromosome.

Figure S2: Smoothed number of bits per symbol (Bps) of GeCo2 subtracted by GeCo3 Bps. The Bps were obtained by referential compression of PT_21 and GG_22, with the same parameters as in Table 3. Places where the line rises above zero indicate that GeCo3 has better compression than GeCo2.

6 Referential hidden nodes effect

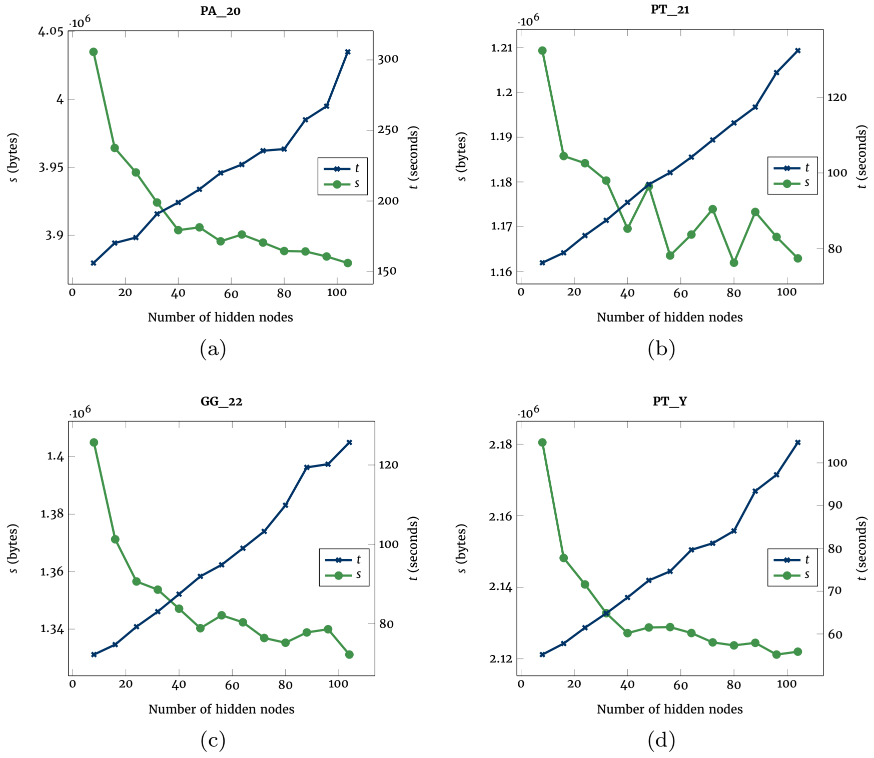

Figure S3: Effect of the number of hidden nodes in reference compressed sequence size and time.

7 Referential histograms

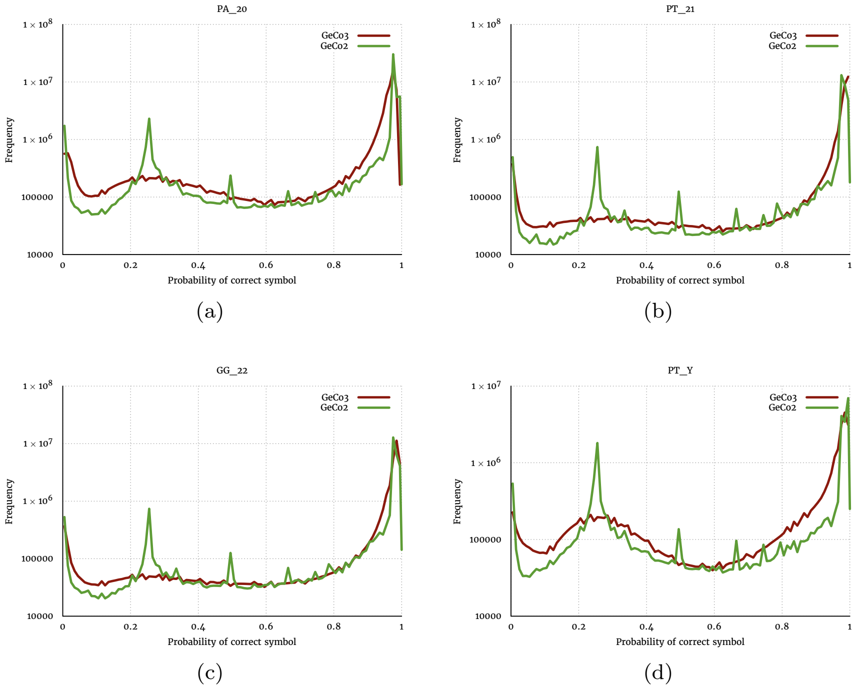

Figure S4: Histograms for GeCo2 and GeCo3 with the vertical axis in a log 10 scale.

8 Reproducibility

8.1 Input data

The input data is constituted by FASTA or FASTQ pre-processed with the following commands:

```
# Convert FASTQ to sequence.
# gto tools can be installed with bioconda
gto_fastq_to_fasta < $name.fastq | gto_fasta_to_seq > $name.seq

# Convert FASTA to sequence.
# Remove all characters that are not ACGT
grep -v ">" < $FASTAFILE | tr "acgt" "ACGT" | tr -d -c "ACGT" > $SEQ

# In the case that the compressor needs the file to be FASTA then the
    following was run:
echo ">" > $SEQF && cat $SEQ >> $SEQF

# For HRCM the sequence could not be all in the same line so the
    following was run:
```

```
fold -w80 $SEQF > $SEQF
```

8.1.1 Downloading the genomes

The genomes were obtained from:

- Denisova: http://cdna.eva.mpg.de/denisova/raw_reads/SL3004_SR.txt.gz and http://cdna.eva.mpg.de/denisova/raw_reads/B1130_SR.txt.gz
- Virome: using fastq-dump https://ncbi.github.io/sra-tools/fastq-dump.html
- PiAbC: ftp://ftp.ncbi.nlm.nih.gov/genomes/all/GCA/900/067/695/GCA_900067695.1_Pabies01/GCA_900067695.1_Pabies01_genomic.fna.gz
- HoSaC and HoSaY: https://www.ncbi.nlm.nih.gov/genome/?term=homo+sapiens
- PaTrC: https://www.ncbi.nlm.nih.gov/genome/?term=pan-troglodytes
- GoGoC: https://www.ncbi.nlm.nih.gov/genome/?term=gorilla+gorilla[orgn]
- Archaea and Virus: using the script from: https://github.com/cobilab/gto/blob/master/scripts/gto_build_dbs.sh. The date of sequence download: 24-04-2020. Alternatively from [Silva et al.(2020)Silva, Pratas, and Pinho].
- Mito: ftp://ftp.ncbi.nlm.nih.gov/refseq/release/mitochondrion/mitochondrion.1.1.genomic.fna.gz
  and
  ftp://ftp.ncbi.nlm.nih.gov/refseq/release/mitochondrion/mitochondrion.2.1.genomic.fna.gz
- All files in dataset 4 available from the supporting data [Silva et al.(2020)Silva, Pratas, and Pinho].

For reference compression:

- HS: https://www.ncbi.nlm.nih.gov/genome/?term=homo+sapiens
- PT: https://www.ncbi.nlm.nih.gov/genome/?term=pan-troglodytes
- GG: https://www.ncbi.nlm.nih.gov/genome/?term=gorilla+gorilla[orgn]
- PA: https://www.ncbi.nlm.nih.gov/genome/?term=Pongo+abelii+genome

8.1.2 Genome identifiers

8.1.3 Virome

```
SRR12175231, SRR12175232, SRR12175233, SRR12175234, SRR12175235,
    SRR12175236, SRR12175237, SRR12175238, SRR12175239, SRR12175240
```

8.1.4 Picea abies

```
GCA_900067695.1
```

8.1.5 Homo sapiens

```
NC_000001.11, NC_000002.12, NC_000003.12, NC_000004.12, NC_000005.10,
NC_000006.12, NC_000007.14, NC_000008.11, NC_000009.12, NC_000010.11
NC_000011.10, NC_000012.12, NC_000013.11, NC_000014.9, NC_000015.10,
NC_000016.10, NC_000017.11, NC_000018.10, NC_000019.10, NC_000020.11,
NC_000021.9, NC_000022.11, NC_000023.11, NC_000024.10, NC_012920.1
```

8.1.6 Pan troglodytes

```
NC_001643.1, NC_006492.4, NC_036879.1, NC_036880.1, NC_036881.1,
NC_036882.1, NC_036883.1, NC_036884.1, NC_036885.1, NC_036886.1,
NC_036887.1, NC_036888.1, NC_036889.1, NC_036890.1, NC_036891.1,
NC_036892.1, NC_036893.1, NC_036894.1, NC_036895.1, NC_036896.1,
NC_036897.1, NC_036898.1, NC_036899.1, NC_036900.1, NC_036901.1,
NC_036902.1
```

8.1.7 Pongo abelii

```
NC_002083.1, NC_036903.1, NC_036904.1, NC_036905.1, NC_036906.1,
NC_036907.1, NC_036908.1, NC_036909.1, NC_036910.1, NC_036911.1,
NC_036912.1, NC_036913.1, NC_036914.1, NC_036915.1, NC_036916.1,
NC_036917.1, NC_036918.1, NC_036919.1, NC_036920.1, NC_036921.1,
NC_036922.1, NC_036923.1, NC_036924.1, NC_036925.1, NC_036926.1
```

8.1.8 Gorilla gorilla

```
NC_011120.1, NC_044602.1, NC_044603.1, NC_044604.1, NC_044605.1,
NC_044606.1, NC_044607.1, NC_044608.1, NC_044609.1, NC_044610.1,
NC_044611.1, NC_044612.1, NC_044613.1, NC_044614.1, NC_044615.1,
NC_044616.1, NC_044617.1, NC_044618.1, NC_044619.1, NC_044620.1,
NC_044621.1, NC_044622.1, NC_044623.1, NC_044624.1, NC_044625.1
```

8.2 Compressors

8.2.1 Downloading the compressors

The compressors where obtained from:

- GeCo2: `https://github.com/cobilab/geco2`
- Jarvis: `https://github.com/cobilab/jarvis`
- XM: `https://github.com/mdcao/japsa/`
- NAF: `https://github.com/KirillKryukov/naf`
- iDoComp: `https://github.com/mikelhernaez/iDoComp`
- GDC2: `https://github.com/refresh-bio/GDC2`

- HRCM: https://github.com/haicy/HRCM

8.2.2 Running the compressors

To run GeCo3:

```
./GeCo3 $OPTIONS $SEQ

# Example of reference free compression, using level 1, BuEb sequence,
    learning rate of 0.06 and 8 hidden nodes.
./GeCo3 -l 1 -lr 0.06 -hs 8 DNACorpus/BuEb

# Example of referential compression, using human chromosome 4 as
    reference and the corresponding gorilla chromosome as target. The
    learning rate is 0.03 and 64 hidden nodes
./GeCo3 -rm 20:500:1:35:0.95/3:100:0.95 -rm 13:200:1:1:0.95/0:0:0 -rm
    10:10:0:0:0.95/0:0:0 -lr 0.03 -hs 64 -r PT_C4 GG_C4
```

To run GeCo2:

```
./GeCo2 $OPTIONS $SEQ

# Example of reference free compression, using level 1 and BuEb sequence
./GeCo2 -l 1 BuEb

# Example of referential compression, using human chromosome 4 as
    reference and the corresponding gorilla chromosome as target
./GeCo2 -rm 20:500:1:35:0.95/3:100:0.95 -rm 13:200:1:1:0.95/0:0:0 -rm
    10:10:0:0:0.95/0:0:0 -r PT_C4 GG_C4
```

To run Jarvis:

```
time ./JARVIS $OPTIONS $SEQ

# Example of reference free compression, using level 1 and BuEb sequence
time ./JARVIS -l 1 BuEb
```

To run XM:

```
jsa.xm.compress $OPTIONS $SEQF

# Example of reference free compression, using BuEb sequence
jsa.xm.compress --real=BuEb.xm BuEb.fasta
```

To run NAF:

```
time ./ennaf $OPTIONS $SEQF

# Example of reference free compression, using BuEb sequence
time ./ennaf --temp-dir /tmp -22 BuEb.fasta
```

To run iDoComp:

```
# Example of referential compression, using human chromosome 4 as
    reference and the corresponding gorilla chromosome as target
REFSEQ=HS_C4
SEQ=GG_C4

cd idocomp
cd sais-lite-2.4.1/
mkdir sa ref tar;
cp $REFSEQ ref/$REFSEQ.fa
./generateSA.sh ref sa
cp $SEQ tar/$SEQ.fa
echo "ref/$REFSEQ.fa tar/$SEQ.fa sa/$REFSEQ.sa" > f.txt;
cp ../simulations/iDoComp.run .
./iDoComp.run c f.txt OUT
```

To run GDC2:

```
time ./GDC2 c xxx $REFSEQ $SEQ

# Example of referential compression, using human chromosome 4 as
    reference and the corresponding gorilla chromosome as target

time ./GDC2 c xxx HS_C4 GG_C4
```

To run HRCM:

```
time ./hrcm compress -r $REFSEQ.fasta -t $SEQ.fasta

# Example of referential compression, using human chromosome 4 as
    reference and the corresponding gorilla chromosome as target

time ./hrcm compress -r HS_C4.fasta -t GG_C4.fasta
```

References

[Silva et al.(2020)Silva, Pratas, and Pinho] Milton Silva, Diogo Pratas, and Armando J Pinho. Supporting data for "Efficient dna sequence compression with neural networks", 2020. URL http://gigadb.org/dataset/100808.
