# Supplementary material for: Efficient DNA sequence compression with neural networks
Source: Gigascience. 2020 Nov 11;9(11):giaa119. doi: 10.1093/gigascience/giaa119 (PMC7657843; doi:10.1093/gigascience/giaa119)
Supplement: giaa119_Response_to_Reviewer_Comments_Original_Submission [file giaa119_response_to_reviewer_comments_original_submission.pdf]

Author's Response To Reviewer Comments

Close

Thank you for the opportunity to revise the manuscript.
The answers to reviewers are included in the attached file GeCo3_Answers.pdf.
As suggested by the editor we have also included the RRID and biotoolsID identifiers in the manuscript.

Close
