# Supplementary material for: Efficient DNA sequence compression with neural networks
Source: Gigascience. 2020 Nov 11;9(11):giaa119. doi: 10.1093/gigascience/giaa119 (PMC7657843; doi:10.1093/gigascience/giaa119)
Supplement: giaa119_Response_to_Reviewer_Comments_Revision_1 [file giaa119_response_to_reviewer_comments_revision_1.pdf]

Author's Response To Reviewer Comments

Close

Thank you so much for the opportunity to submit a revised manuscript. We would also like to thank the reviewers. Their comments where very helpful in improving the manuscript.

We have corrected the reference mentioned by reviewer #1.

We have added the ORCIDs of the corresponding authors. We have not included the ORCID of Armando J Pinho https://orcid.org/0000-0002-9164-0016 in the manuscript because we were unsure where to put it. However feel free to include it in the publication.

We have updated the Availability of Supporting data section with the corresponding DOI.

The RRID for geco3 is in the main manuscript and the accessions are in the supplementary material, as well as the download locations.

We have added two of the datasets used in the manuscript to the Supporting data to make it easier to reproduce the results. We have also mentioned this in the supplementary material.

If there is anything that we missed please let us know.

Close
