# Supplementary material for: Efficient DNA sequence compression with neural networks
Source: Gigascience. 2020 Nov 11;9(11):giaa119. doi: 10.1093/gigascience/giaa119 (PMC7657843; doi:10.1093/gigascience/giaa119)
Supplement: giaa119_Reviewer_1_Report_Original_Submission — Kirill Kryukov, Ph.D. -- 6/13/2020 Reviewed [file giaa119_reviewer_1_report_original_submission.pdf]

Reviewer Report

Title: Efficient DNA sequence compression with neural networks

Version: Original Submission **Date:** 6/13/2020

Reviewer name: Kirill Kryukov, Ph.D.

Reviewer Comments to Author:

Authors describe GeCo3 - a new compressor for DNA data.
GeCo3 uses neural network to mix several context models of the input sequence.
The models themselves are mostly from GeCo2.
I downloaded GeCo3 and tested it on my data. It generally performed as described in the paper.
I noticed and reported a data corruption issue on some data.
The authors quickly fixed the problem, which allowed me to complete the testing.
The paper is well organized and has good attention to detail. The background section is comprehensive.
The method is described and benchmarked in sufficient detail.
The method seems to be an improvement compared to previous work.
Therefore I think it can be published, after addressing the comments below.
Major comments:
1. GeCo3 makes heavy use of a floating point math. It is known to have potential for producing different results based on factors such as CPU and compiler (and compiler version).
In the hypothetical scenario of using GeCo3 for long term data storage (as suggested in the paper), data compressed on one machine should be possible to decompress on another, possibly quite different, machine.
I am currently not convinced that GeCo3 can be trusted for such application.
I think such scenario should be tested or otherwise addressed.
2. In the paper GeCo3 is compared with other specialized compressors, namely GeCo2, Jarvis, XM and NAF (for reference-free compression).
I think it would makes sense to include state-of-the-art general-purpose compressors in comparison, such as "zpaq -5" and cmix.
(cmix is extremely slow, so possibly it can be used on smaller test datasets).
Including such compressors may be informative for exploring the limits of compression strength available today.
In my experience cmix often outperforms specialized compressors in terms of compression strength.
Minor comments:
1. In the output of "GeCo3 -h", the description of "-l" and "--level" arguments should mention the acceptable range of levels.
2. Manuscript uses too much unnatural and confusing phrasing, and should be edited for clarity.
3. "Other general-purpose followed the same line, namely Cmix [82], DeepZip [83], and DeepDNA [63]."
- I don't think DeepDNA can be called general-purpose?

Level of Interest

Please indicate how interesting you found the manuscript: Choose an item.

Quality of Written English

Please indicate the quality of language in the manuscript: Choose an item.

Declaration of Competing Interests

Please complete a declaration of competing interests, considering the following questions:

- Have you in the past five years received reimbursements, fees, funding, or salary from an organisation that may in any way gain or lose financially from the publication of this manuscript, either now or in the future?
- Do you hold any stocks or shares in an organisation that may in any way gain or lose financially from the publication of this manuscript, either now or in the future?
- Do you hold or are you currently applying for any patents relating to the content of the manuscript?
- Have you received reimbursements, fees, funding, or salary from an organization that holds or has applied for patents relating to the content of the manuscript?
- Do you have any other financial competing interests?
- Do you have any non-financial competing interests in relation to this paper?

If you can answer no to all of the above, write 'I declare that I have no competing interests' below. If your reply is yes to any, please give details below.

Potential non-financial competing interest: I have previously developed a compressor for DNA sequences (NAF). It could possibly be considered a competitor to the compressor described in this paper.

I agree to the open peer review policy of the journal. I understand that my name will be included on my report to the authors and, if the manuscript is accepted for publication, my named report including any attachments I upload will be posted on the website along with the authors' responses. I agree for my report to be made available under an Open Access Creative Commons CC-BY license (http://creativecommons.org/licenses/by/4.0/). I understand that any comments which I do not wish to be included in my named report can be included as confidential comments to the editors, which will not be published.

Choose an item.

To further support our reviewers, we have joined with Publons, where you can gain additional credit to further highlight your hard work (see: https://publons.com/journal/530/gigascience). On publication of this paper, your review will be automatically added to Publons, you can then choose whether or not to claim your Publons credit. I understand this statement.

Yes Choose an item.
