# Supplementary material for: Efficient DNA sequence compression with neural networks
Source: Gigascience. 2020 Nov 11;9(11):giaa119. doi: 10.1093/gigascience/giaa119 (PMC7657843; doi:10.1093/gigascience/giaa119)
Supplement: giaa119_Reviewer_2_Report_Original_Submission — Mikel Hernaez -- 7/3/2020 Reviewed [file giaa119_reviewer_2_report_original_submission.pdf]

Reviewer Report

Title: Efficient DNA sequence compression with neural networks

Version: Original Submission **Date:** 7/3/2020

Reviewer name: Mikel Hernaez

Reviewer Comments to Author:

The authors proposed a compression method for compressing a set of DNA sequences (in FASTA format), that improve upon their previously proposed method GeCo2.
Overall, the paper is very well written, the proposed new technology is concisely explained and a good set of benchmarks have been done to assess the performance of the propose method.
However I have several concerns:
1) My main concern is that the compression gain obtained with the proposed method is not very significant if you take into account the overhead in computation needed to achieve those gains. It would be interesting to do some rough numbers justifying the improvement (for example how much would it cost to compress/decompress in resources, vs. how much one would save on storage). This could make a stronger argument on why these "small" improvements are needed.
2) While I agree with the reasoning on why the problem of compressing FASTA files composed of set of DNA sequences is important, I would encourage the authors to show how this methodology would work on NGS data in form of FASTQ files (assuming that QVs are compressed independently). Also, I believe that mentioning very large Genome projects, such the Earth BioGenome Project (https://www.earthbiogenome.org/) whose goal is to generate the genome of every species in the world, is important to make a case on how those databases would benefit from the proposed technology.
3) A comparison should be done with general-purpose compressors based on Neural networks, such as DeepZip. I would also include another comparison showing the performance of using DeepZip to compress the information from GeCo2 that is fed into the neural network. This would allow the reader to understand the true gain of the core technology proposed in this work. I would also recommend testing generalize compressors based on mixture of experts such as PAQ8, as the final compressor rather than a neural network.
4) I would also recommend including re-sequenced-based compression to the comparative. I understand that the method is design for more divergent genomes (i.e., different species), but adding the same-species-reference compression would complete nicely the assessment
Minor comments:
1) The ID of the sequences used, such as ScPo, are mentioned in the text without prior notice. I would recommend clarifying this before mentioning them, such as in page 5 with the mention of ScPo.
2) Page 5 Additional --> Additionally

Level of Interest

Please indicate how interesting you found the manuscript: Choose an item.

Quality of Written English

Please indicate the quality of language in the manuscript: Choose an item.

Declaration of Competing Interests

Please complete a declaration of competing interests, considering the following questions:

- Have you in the past five years received reimbursements, fees, funding, or salary from an organisation that may in any way gain or lose financially from the publication of this manuscript, either now or in the future?
- Do you hold any stocks or shares in an organisation that may in any way gain or lose financially from the publication of this manuscript, either now or in the future?
- Do you hold or are you currently applying for any patents relating to the content of the manuscript?
- Have you received reimbursements, fees, funding, or salary from an organization that holds or has applied for patents relating to the content of the manuscript?
- Do you have any other financial competing interests?
- Do you have any non-financial competing interests in relation to this paper?

If you can answer no to all of the above, write 'I declare that I have no competing interests' below. If your reply is yes to any, please give details below.

I declare that I have no competing interest

I agree to the open peer review policy of the journal. I understand that my name will be included on my report to the authors and, if the manuscript is accepted for publication, my named report including any attachments I upload will be posted on the website along with the authors' responses. I agree for my report to be made available under an Open Access Creative Commons CC-BY license (http://creativecommons.org/licenses/by/4.0/). I understand that any comments which I do not wish to be included in my named report can be included as confidential comments to the editors, which will not be published.

Choose an item.

To further support our reviewers, we have joined with Publons, where you can gain additional credit to further highlight your hard work (see: https://publons.com/journal/530/gigascience). On publication of this paper, your review will be automatically added to Publons, you can then choose whether or not to claim your Publons credit. I understand this statement.

Yes Choose an item.
